# Telomere-to-telomere genome assembly of *Electrophorus electricus* provides insights into the evolution of electric eels

**DOI:** 10.1093/gigascience/giaf024

**Published:** 2025-04-01

**Authors:** Zan Qi, Qun Liu, Haorong Li, Yaolei Zhang, Ziwei Yu, Wenkai Luo, Kun Wang, Yuxin Zhang, Shoupeng Pan, Chao Wang, Hui Jiang, Qiang Qiu, Wen Wang, Guangyi Fan, Yongxin Li

**Affiliations:** School of Ecology and Environment, Northwestern Polytechnical University, Xi'an 710072, China; BGI-Qingdao, BGI-Shenzhen, Qingdao 266555, China; School of Ecology and Environment, Northwestern Polytechnical University, Xi'an 710072, China; BGI-Qingdao, BGI-Shenzhen, Qingdao 266555, China; School of Ecology and Environment, Northwestern Polytechnical University, Xi'an 710072, China; School of Ecology and Environment, Northwestern Polytechnical University, Xi'an 710072, China; School of Ecology and Environment, Northwestern Polytechnical University, Xi'an 710072, China; School of Ecology and Environment, Northwestern Polytechnical University, Xi'an 710072, China; School of Ecology and Environment, Northwestern Polytechnical University, Xi'an 710072, China; School of Ecology and Environment, Northwestern Polytechnical University, Xi'an 710072, China; College of Life Sciences, Hainan Normal University, Haikou 571158, China; School of Ecology and Environment, Northwestern Polytechnical University, Xi'an 710072, China; School of Ecology and Environment, Northwestern Polytechnical University, Xi'an 710072, China; BGI-Qingdao, BGI-Shenzhen, Qingdao 266555, China; School of Ecology and Environment, Northwestern Polytechnical University, Xi'an 710072, China

**Keywords:** electric eel, *Electrophorus electricus*, telomere-to-telomere, genome assembly, genome annotation, evolution

## Abstract

**Background:**

Electric eels evolved remarkable electric organs that enable them to instantaneously discharge hundreds of volts for predation, defense, and communication. However, the absence of a high-quality reference genome has extremely constrained the studies of electric eels in various aspects.

**Results:**

Using high-depth, multiplatform sequencing data, we successfully assembled the first telomere-to-telomere high-quality reference genome of *Electrophorus electricus*, which has a genome size of 833.43 Mb and comprises 26 chromosomes. Multiple evaluations, including N50 statistics (30.38 Mb), BUSCO scores (97.30%), and mapping ratio of short-insert sequencing data (99.91%), demonstrate the high contiguity and completeness of the electric eel genome assembly we obtained. Genome annotation predicted 396.63 Mb repetitive sequences and 20,992 protein-coding genes. Furthermore, evolutionary analyses indicate that Gymnotiformes, which the electric eel belongs to, has a closer relationship with Characiformes than Siluriformes and diverged from Characiformes 95.00 million years ago. Pairwise sequentially Markovian coalescent analysis found a sharply decreased trend of the population size of *E. electricus* over the past few hundred thousand years. Furthermore, many regulatory factors related to neurotransmitters and classical signaling pathways during embryonic development were significantly expanded, potentially contributing to the generation of high-voltage electricity.

**Conclusions:**

This study not only provided the first high-quality telomere-to-telomere reference genome of *E. electricus* but also greatly enhanced our understanding of electric eels.

## Introduction

The natural principles of “Law of the Jungle” and “Survival of the Fittest” underscore the paramount importance of animals’ abilities in predation and defense. As one of the oldest vertebrate groups, fishes, despite their rich biodiversity, largely adopt extremely conservative feeding and defense strategies that rely primarily on biting. However, after a long evolutionary process, strong electric fishes have abandoned traditional biting methods and instead use high-voltage electric shocks as their predation and defense strategy [1, 2]. Nowadays, there are at least 3 main living groups of strongly electric fishes on Earth, including electric eel, electric catfish, and electric ray [1]. Among them, electric eels are the ones that boast the strongest electric shock capacity, mainly distributed in the Amazon River basin [2]. Previous studies have indicated that the ability of strongly electric fishes to instantly release high-voltage electricity is mainly attributed to their evolved new organ: electric organs [1]. Interestingly, besides the one strong electric organ that is shared by all 3 groups (known as the main electric organ in electric eels; Main EO), electric eels have also evolved 2 additional weak electric organs: Hunter’s electric organ (Hunter’s EO) and Sach’s electric organ (Sach’s EO), which are mainly used to sense the surrounding environment and for communication [3]. Anatomical and electrophysiological studies have revealed that discharge cells, specifically known as electrocytes, constitute a substantial part of the bodies of strongly electric fishes [1, 4]. Their unique serial battery-like arrangement of electrocytes within their electric organs (EOs) is the underlying factor enabling these strongly electric fish to instantaneously discharge hundreds of volts of electricity [1]. For that reason, the body length of strongly electric fishes determines the voltage they can generate [4]. According to previous records, the adult electric eel can instantaneously discharge approximately 600–800 volts of high-voltage electricity [2]. Therefore, it becomes particularly crucial to conduct comprehensive and systematic studies on the remarkable innovative characteristics of electric eels, especially exploring the composition of their electric organs and the mechanisms of their high-voltage discharge.

In recent years, the rapid development of genome sequencing technology has significantly expedited the study progress across various life science disciplines. Excitingly, the emergence of long-read sequencing technologies, notably Oxford Nanopore Technologies (ONT) and Pacific Biosciences (PacBio), has presented an opportunity to assemble genomes up to the telomere-to-telomere level. Still now, several important species have achieved telomere-to-telomere level assembly, and many complex questions have been solved with the help of the high-quality reference genomes [5]. However, only a limited number of animal species, such as human, rodent, chicken and zigzag eel [6–9], have been reported to have achieved telomere-to-telomere level assembly. During its long evolutionary process, the electric eel has developed numerous fascinating and unique biological traits [1, 3, 4]. However, despite the availability of publicly accessible electric eel genome assemblies on NCBI (e.g., GCA_013358815.1), the contiguity and completeness of these genomes are often insufficient to meet the standards of high-precision and systematic comparative genomics research.

In this study, combining multiple sequencing data, we successfully assembled the first telomere-to-telomere high-quality reference genome of the electric eel, *Electrophorus electricus* (NCBI:txid8005), with a genome size of 833.43 Mb and comprising 26 chromosomes. Multiple evaluations, including N50 statistics, BUSCO scores, and the mapping ratio of short-insert sequencing data, indicate the high contiguity and completeness of the electric eel genome assembly we obtained. Genome annotation identified 396.63 Mb repetitive sequences and 20,992 protein-coding genes. Evolutionary analyses indicate that Gymnotiformes, which the electric eel belongs to, has a closer relationship with Characiformes than Siluriformes and diverged from Characiformes 95.00 million years ago. Pairwise sequentially Markovian coalescent analysis found a sharply decreased trend of the population size of *E. electricus* over the past few hundred thousand years. Furthermore, many regulatory factors related to neurotransmitters and classical signaling pathways during embryonic development were significantly expanded, potentially contributing to the generation of high-voltage electricity. This study presents the first high-quality telomere-to-telomere reference genome of the electric eel, marking a significant milestone and opening up valuable opportunities for future comprehensive studies of the exceptional characteristics of strongly electric fishes.

## Methods

### Sampling, library construction, and sequencing

An individual electric eel (*E. electricus*) used in this study was procured from the aquatic pet market in China. Fresh tissues were dissected and subsequently sent to the biological company of Benagen and Novogene for a range of genomic analyses. These included DNA extraction, library construction, and whole-genome sequencing, utilizing various sequencing technologies. Specifically, ultra-long genome sequencing was conducted using the Oxford Nanopore Technologies (ONT) platform, while HiFi sequencing employed the Pacific Biosciences (PacBio) platform. Additionally, Hi-C sequencing and short-insert sequencing were performed on the Illumina platform. All experimental operations with animals adhered to relevant standards of animal ethics and welfare of Northwestern Polytechnical University.

### Quality control of raw sequencing data

For the short-insert reads generated from the Illumina platform, all low-quality reads/bases, duplicated reads, and adapter sequences were filtered out using Perl scripts. For Nanopore long reads, we calculated the mean quality score for each read, retaining only those that met the criteria of having a mean quality score of ≥7 and a length of ≥1 Kb. For PacBio long reads, CCS (v6.0.0) was used to remove low-quality reads, applying the parameters of “-min-passes 3 -min-length 10 -min-rq 0.99.”

### Estimation of genome size

To investigate the genome characteristics of *E. electricus*, a *k-*mer–based approach was implemented utilizing the cleaned short-insert sequencing reads obtained from the Illumina platform. The genome size (G) can be estimated using the following formula: G = TN_17-mer_/PFD_17-mer_, where TN_17-mer_ denotes the total number of 17-mers and PFD_17-mer_ represents the peak frequency depth of the 17-mers. Specifically, the 17-mers were counted using KmerFreq (v1.0) with the parameters “-k 17,” and then these data were used to estimate the genome size by running the GenomeAnalysis.pl script. In addition, the genome size has also been evaluated by GCE (RRID:SCR_017332) (v1.0.2) [10] using 17-mers.

### Genome assembly

To achieve a high-quality genome assembly of *E. electricus*, a multistep assembly strategy was employed. (i) The contig-level genome was assembled using Hifiasm (RRID:SCR_021069) (v0.19.5-r592) [11] based on both HiFi reads and ultra-long sequencing reads, with the default parameters except for setting the “-D” option to 10. (ii) Potential base errors generated during the sequencing process in the contig-level genome were corrected using Pilon (RRID:SCR_014731) (v1.22) [12] with default parameters, based on the short-insert sequencing reads. (iii) The contigs of the corrected genome assembly were extended using Lrscaf (v1.1.10) [13] with default parameters except “-t mm,” based on the ultra-long sequencing reads. (iv) The extended contig-level genome was anchored into chromosomes based on the analysis of Hi-C sequencing data using Juicer (RRID:SCR_017226) (v1.6) [14] and 3-dimensional *de novo* assembly (v170123) [15] workflow with the parameters of “-m haploid -i 15000 -r 2.” (v) The ultra-long sequencing reads generated from the ONT platform were assembled into a contig-level genome using NextDenovo (RRID:SCR_025033) (v2.5.2) [16], with the parameters of “read_type = ont, seed_cutoff = 109,337, read_cutoff = 1k, minimap2_options_cns = -x ava-ont -t 15 -k17 -w17.” (vi) Potential base errors generated during the sequencing process in the contig-level genome were corrected using NextPolish (RRID:SCR_025232) (v1.4.1) [17], based on HiFi reads and clean short reads, with the parameters of “sgs options=-max_depth 100, HiFi options=-max_depth 150, HiFi minimap2 options=-x map PB.” (vii) The gaps in the chromosome-level genome assembly were filled using TGS-gapcloser (v1.2.1) [18], based on the corrected ultra-long contig-level assembly, with the parameters of “–min_nread 1 –min_match 2000 –minmap_arg ‘-x asm5’.” (viii) Potential base errors in the gap-closed genome assembly were further corrected using Pilon (v1.22) [12] with default parameters, based on the short-insert sequencing reads.

### Quality evaluation of genome assembly

Multiple strategies were employed to evaluate the quality of the genome assembly. (i) The completeness of conserved core genes in the actinopterygii database was analyzed for the genome using BUSCO (RRID:SCR_015008) (v5.4.5) [19]. (ii) The cleaned short-insert sequencing reads generated on the Illumina platform were aligned to the genome with BWA (v0.7.17) [20] using the parameters of “bwa mem -M,” and the proportion of properly mapped reads was determined using the *flagstat* function of SAMTools (RRID:SCR_002105) (v1.9) [21]. (iii) The contiguity of the genome was evaluated using the N50 score, which was calculated with a custom Perl script.

### Annotation of repetitive sequences

To identify the repetitive sequences in the *E. electricus* genome, including tandem repeats and transposable elements (TEs), we integrated a homology-based prediction using the Repbase library and a *de novo* prediction based on self-sequence alignment and repetitive sequence features. First, tandem repeats were annotated using Tandem Repeat Finder (RRID:SCR_022193) (v4.10) [22] with the parameters of “Match = 2, Mismatch = 7, Delta = 7, PM = 80, PI = 10, Minscore = 50, MaxPeriod = 2000 -d -h.” Second, TEs were predicted on both DNA and protein levels. On the DNA level, RepeatModeler software (RRID:SCR_015027) (v2.0.1) was used to construct the *de novo* repeat library. RepeatMasker (RRID:SCR_012954) (v4.0.5) [23] was then run separately against the *de novo* library and the repbase library to identify repetitive sequences with parameters of “-nolow -no_is -norna.” Third, on the protein level, RepeatProteinMask (v1.36) was used to search TEs in its protein database with the parameters of “-noLowSimple -pvalue 0.0001.” Finally, the annotation results generated from different annotation strategies were integrated to produce the final annotation of repetitive sequences. The telomere and centromere regions were predicted according to the quarTeT (RRID:SCR_025258) (v1.1.8) [24].

### Annotation of protein-coding genes

Multiple strategies, including the *de novo*–based prediction, homology-based prediction, and transcript-based prediction, were employed for annotating the protein-coding genes of the *E. electricus* genome. (i) For *de novo*–based prediction, BRAKER3 [25] was employed with default parameters based on the assembled transcripts. (ii) For homology-based prediction, protein sequences from 10 species, including *Homo sapiens* (GCF_000001405.40), *Clarias gariepinus* (GCF_024256425.1), *Hemibagrus wyckioides* (GCF_019097595.1), *Ictalurus punctatus* (GCF_001660625.3), *Mus musculus* (GCF_000001635.27), *Pangasianodon hypophthalmus* (GCF_027358585.1), *Silurus meridionalis* (GCF_014805685.1), *Tachysurus fulvidraco* (GCF_022655615.1), *Tachysurus vachellii* (GCF_030014155.1), and *Danio rerio* (GCF_000002035.6), were downloaded from NCBI database. All downloaded genes were aligned to the genome using BLAST (v2.6.0) [26] with the parameters of “e-value 1e-5 -p tblastn -m 8.” Genewise (RRID:SCR_015054) (v2.2.0) [27] was used to identify the longest coding regions and/or highest score in each gene locus to support the presence of a homologous gene with the parameters of “-tfor -pseudo -pretty -sum -gff -genesf.” (iii) For transcript-based prediction, the coding regions were first *de novo* assembled utilizing the Hisat2 (RRID:SCR_015530) (v2.2.1) and StringTie (RRID:SCR_016323) (v2.1.4) workflow [28, 29], both of which were employed with default parameters using our previous RNA sequencing (RNA-seq) data (PRJNA592729). Subsequently, TransDecoder (RRID:SCR_017647) (v5.5.0) was employed to predict transcripts. These transcripts were then mapped onto the reference genomes using BLAT (RRID:SCR_011919) (v36) [30], and the gene structure was predicted by GeneWise (v2.2.0) [27] with default parameters. Finally, the results generated from these 3 strategies were integrated into a final gene set using EvidenceModeler (RRID:SCR_014659) (v.1.1.1) [31] with the parameters of “–segmentSize 5000000 –overlapSize 50000.”

### Functional annotation of protein-coding genes

To enhance the understanding of the predicted genes, all protein-coding genes were aligned against multiple databases for functional annotation. These databases include Gene Ontology (RRID:SCR_002811) (GO), the Integrated Resource of Protein Domains and Functional Sites (InterPro) (RRID:SCR_006695), the Kyoto Encyclopedia of Genes and Genomes (KEGG), SwissProt, TrEMBL, and the nonredundant protein database (NR). The alignment to the InterPro database was performed using InterProScan (RRID:SCR_005829) (v5.45–80.0) [32] with the parameters “-dp -f tsv -iprlookup -goterms.” For the other annotation processes, BLAST (v2.6.0) [26] was utilized with the parameters “-b 100 -v 100 -p blastp -e 1e-05 -F F.” For each gene, the annotation term with the highest score was retained as the final functional annotation.

### Identification of orthologous genes

Orthologous genes among 7 species, including *D. rerio* (GCF_000002035.6), *Ictalurus punctatus* (GCF_001660625.3), *Pygocentrus nattereri* (GCF_015220715.1), *Tachysurus fulvidraco* (GCF_022655615.1), *Astyanax mexicanus* (GCF_023375975.1), *Trichomycterus rosablanca* (GCF_030014385.1), and *E. electricus*, were identified for the comparative genomic analyses. First, the longest transcript for each gene was solely retained among these species with a custom Perl script. Second, the reciprocal best BLAST hit of each gene pairs was employed using BLAST [26] with the parameters of “-evalue 1e-5 -outfmt 6.” Third, pairwise orthologous relationships were identified among these species using OrthoMCL (RRID:SCR_007839) (v2.0.9) [33] with the default parameters.

### Inference of phylogenetic relationships

The protein sequences of the single-copy orthologous genes, identified among the 7 species, were aligned using MUSCLE (RRID:SCR_011812) (v3.8.31) [34] with default parameters. Then, using *D. rerio* as the outgroup species, we constructed phylogenetic trees for each gene using RAxML (RRID:SCR_006086) (v8.2.10) [35] with the parameters of “-f a -m PROTGAMMAAUTO -p 12345 -T 30 -x 12345 -N 100” and IQ-TREE (RRID:SCR_017254) (v2.2.0) [36] with the parameters of “-m JTT+C60+F -msub nuclear -B 1000 -alrt 1000 –seqtype AA,” respectively. Finally, the species tree was inferred by ASTRAL (RRID:SCR_001886) (v5.7.1) [37] with default parameters based on the constructed gene trees.

### Inference of divergence time

To accurately estimate the divergence times among species, we employed an approach that integrated the phylogenetic tree, 4dTVs (4-fold degenerate synonymous sites) data extracted from identified single-copy orthologous genes, and fossil-calibrated information sourced from the TIMETREE database. This multifaceted dataset was then utilized within the MCMCtree model, implemented in PAML (RRID:SCR_014932) (v4.4) [38], to infer the divergence times.

### Relative evolutionary rate of species

To compare the relative evolutionary rates between *E. electricus* and other fish species, we first concatenated the sequences of single-copy orthologous genes into a supergene for each species. Subsequently, we performed multiple sequence alignment of these supergenes using MUSCLE (v3.8.31) [34] with default parameters. Finally, we analyzed the relative evolutionary rates of these species using the LINTRE program (v1.1) [39], designating *E. electricus* as the reference species and *D. rerio* as the outgroup species.

### Dynamic change of population history

To obtain a comprehensive understanding of the population status of *E. electricus*, we conducted an analysis of the dynamic changes in its population history spanning the recent past. First, the short-insert sequencing data were mapped to the reference genome using BWA (v0.7.17) [20] with the command “bwa mem -M.” Second, SAMtools (v1.13) [21] converted the aligned results to *bam* format with “view -bS,” sorted the resulting *bam* file using “samtools sort,” and created an index for the sorted file with “samtools index.” Third, single-nucleotide polymorphisms were detected using BCFtools (RRID:SCR_005227) (v1.16) [40] through the sequential commands “bcftools mpileup -d 150 -q 20 -Q 20” for pileup generation and “bcftools call -c” for variant calling. Following detection, the format of the resulting *VCF* file was refined using the *vcfutils.pl* script from BCFtools (v1.16) [40]. Based on these results, a final PSMC (RRID:SCR_017229) (v0.6.5-r67) analysis [41] was conducted with 100 bootstrap replicates, utilizing the parameters “-N 25 -r 5 -p ‘4+25*2+4+6’.” The nucleotide substitution rate for this species, measured as substitutions per site per million years, was estimated using 4-fold degenerate sites and fossil information via the r8s software (RRID:SCR_021161) (v1.2) [42]. The per-generation mutation rate was then estimated by multiplying the per-nucleotide substitution rate by the generation time. Finally, these results were scaled to absolute time and population size using the generation time and estimated per-generation mutation rate. This was accomplished by first running *psmc2history.pl* with the default parameters and then using the acquired values for the *-g* (generation time) and *-μ* (mutation rate per generation) parameters with *history2ms.pl* to perform the scaling.

### Expansion and contraction of gene family

Based on the results of gene families identified by OrthoMCL (v2.0.9) [33] and the divergence-timed phylogenetic tree derived from PAML (v4.4) [38], we employed the random birth-and-death model in CAFE (RRID:SCR_005983) (v4.2.1) [43] to investigate the dynamics of gene family expansions and contractions. If the copy number of the gene family in the detected branch lineage was higher/lower than that of its most recent common ancestral branch, then the gene family was defined as being substantially expanded/contracted in the detected lineage.

### Functional enrichment

To carry out functional enrichment analysis, such as the GO enrichment, we adopted a workflow that integrated EggNOG mapper software (RRID:SCR_005983) (v2.1.12) [44] and clusterProfiler (RRID:SCR_016884) (v4.6.2) [45]. Functional annotation of the whole gene set of *E. electricus* was conducted using the EggNOG mapper software (v2.1.12), leveraging the EggNOG database (v5.0). From these annotations, unique identifier numbers for each gene in the GO database were extracted. Subsequently, 2 files were prepared: an interest gene list and a total gene list, both adhering to the specified format. The interest gene list contained the gene IDs of genes of interest, while the total gene list included all gene IDs along with their corresponding GO IDs. Functional enrichment analysis was then performed using the enrichGO functions within the clusterProfiler R package (v4.6.2) [45].

## Results

### High-quality reference genome assembly of the electric eel

**Figure 1:**
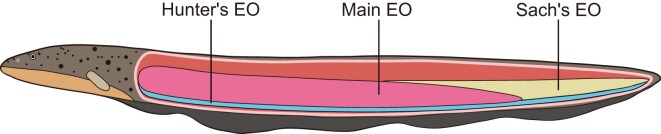
Schematic diagram of the electric eel (*E. electricus*). Only the 3 electric organs are marked.

The evolution of electric organs in electric eels, featuring a main electric organ (Main EO) and 2 additional weak electric organs (Hunter’s EO and Sach’s EO), represents one of their most innovative and distinctive characteristics (Fig. 1). Therefore, a genome of the electric eel with higher contiguity and completeness is urgently needed. To investigate the genomic characteristics of *E. electricus* (electric eel), we generated a substantial amount of short-insert sequencing reads (97.17 Gb) using the Illumina platform (Supplementary Table S1). Our *k*-mer analysis identified a prominent main peak (homozygous peak) at a *k*-mer depth of 83, and we calculated the genome size of *E. electricus* to be close to 800 Mb (Supplementary Table S2 and Supplementary Fig. S1). To facilitate the assembly of a high-quality reference genome of *E. electricus*, we further generated diverse sequencing data from multiple platforms, including Oxford Nanopore ultra-long reads (46.31 Gb), PacBio HiFi reads (59.65 Gb), and Illumina Hi-C reads (225.59 Gb) (Supplementary Table S1). Considering the respective advantages of these 2 types of long-read sequencing data, we assembled the contig-level genome using Hifiasm [11] by simultaneously utilizing both types of data (HiFi reads and ultra-long reads), resulting in an 817.93 Mb genome assembly with an N50 length of 21.44 Mb and average contig length of 1.56 Mb (Supplementary Table S3). Furthermore, we refined the assembly by correcting potential base errors using short-insert sequencing data and further extended the sequence length with ultra-long reads, which remarkably improved the contiguity of the *E. electricus* genome (N50 length: 21.44 Mb; average contig length: 1.72 Mb; Supplementary Table S3). To further achieve a chromosome-level genome assembly, we anchored the extended contig-level genome into chromosomes using 3D-DNA software (v170123) [15], based on high-depth Hi-C sequencing reads (Supplementary Table S1). This resulted in a genome assembly of 823.20 Mb, with 26 chromosomes successfully assembled (Supplementary Tables S3 and S4). Subsequently, we filled the gaps in the chromosome-level genome assembly by utilizing the polished contig-level assembly and further examined the results through the mapping of ultra-long reads (Supplementary Fig. S2). Finally, to remove potential base sequencing errors, we further polished the genome assembly after closing the gaps, based on Illumina short reads, resulting in a final 833.43 Mb genome of *E. electricus* (Supplementary Table S4). To comprehensively evaluate the quality of the *E. electricus* genome, we employed multiple evaluation strategies, including N50 length (30.38 Mb; Table 1), BUSCO scores (97.30%; Table 2), and the mapping ratio of short-insert sequencing reads (99.91%; Supplementary Table S5). These results indicate that we successfully obtained a high-contiguity and high-integrity genome assembly for the electric eel (Table 1).

**Table 1:** Statistics of the *E. electricus* genome

Term	Size/number
Genome size (bp)	833,427,914
Number of chromosomes	26
Number of contigs	479
Number of scaffolds	460
Contig N50 (bp)	21,439,367
Scaffold N50 (bp)	30,383,234
Number of telomeres	46
GC percent (%)	41.5

**Table 2:** BUSCO assessment of the *E. electricus* genome

Term	Number	Percentage (%)
Complete BUSCOs (C)	3,541	97.3
Complete and single-copy BUSCOs (S)	3,448	94.7
Complete and duplicated BUSCOs (D)	93	2.6
Fragmented BUSCOs (F)	34	0.9
Missing BUSCOs (M)	65	1.8
Total BUSCO groups searched	3,640	100

### Genome annotation of the electric eel

**Figure 2:**
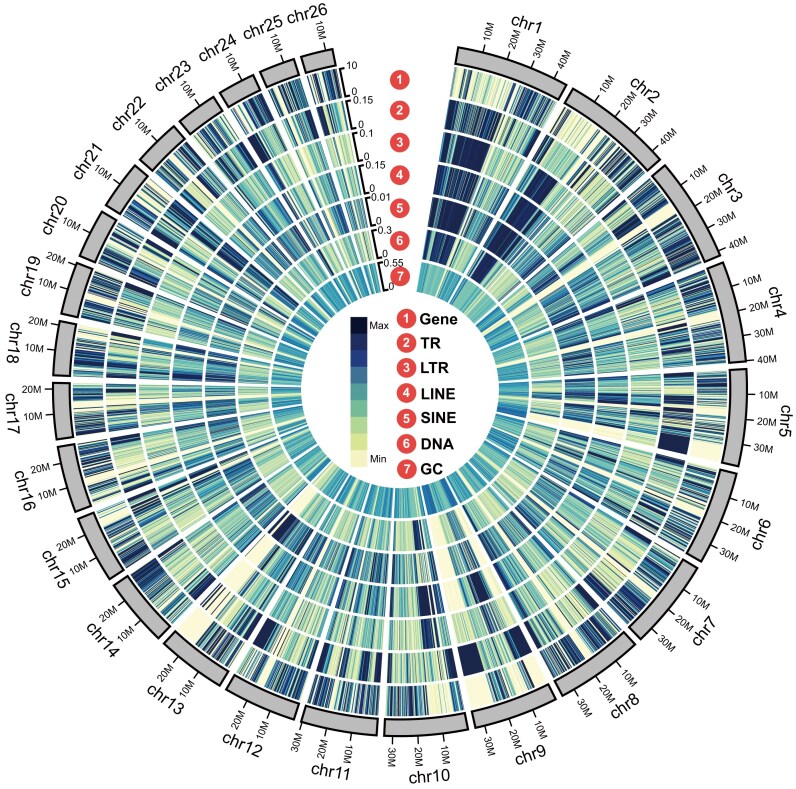
Distributions of the genomic elements in *E. electricus*. In the circos plot, the outermost layer displays the distribution of protein-coding genes, followed by tandem repeats (TRs), long tandem repeats (LTRs), long/short interspersed nuclear elements (LINEs/SINEs), DNA elements, and, finally, the GC content at the innermost layer. The color bar indicates the number/percent of each genomic element within the plot. As the color darkens, it signifies an increase in the percentage or number of that particular genomic element.

**Figure 3:**
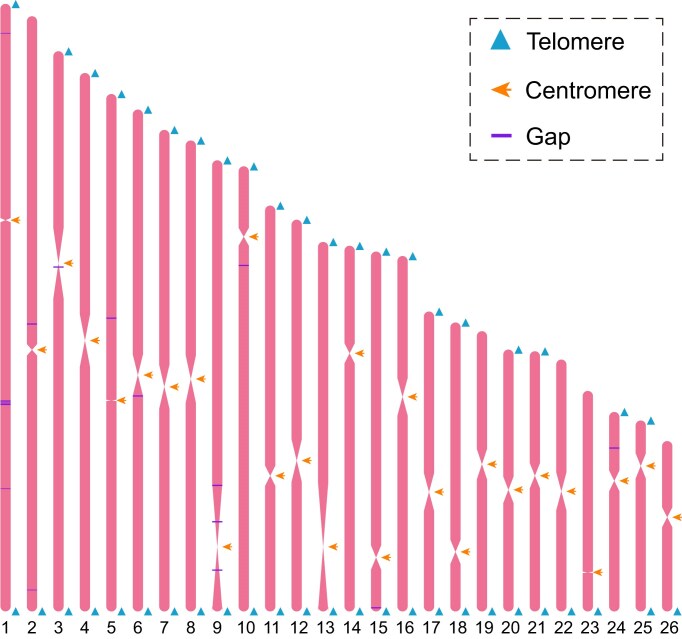
Distributions of telomeres, centromeres, and gaps in the genome of *E. electricus*. The specific meaning of each symbol is indicated in the figure.

To comprehensively understand the genome composition, such as repetitive sequences and protein-coding genes, we performed the genome annotation based on multiple strategies. After that, a total of 396.63 Mb of repetitive sequences were predicted, accounting for 47.59% of the *E. electricus* genome (Supplementary Table S6). Specifically, TE statistics reveal that DNA transposons are the most abundant type, accounting for 12.03% of the genome assembly with a total size of 100.26 Mb. Subsequently, long interspersed nuclear elements (LINEs) comprise 11.67% of the genome, totaling 97.26 Mb. Long terminal repeats (LTRs) occupy 4.65% of the genome, with a size of 38.76 Mb, while short interspersed nuclear elements (SINEs) constitute only 0.99%, amounting to 8.22 Mb in total (Supplementary Table S7). Furthermore, using 3 different annotation strategies, we successfully predicted 20,992 protein-coding genes, and 97.19% of the predicted genes were successfully annotated in public databases (Supplementary Table S8). Besides, the quality of the predicted genes is comparable to that of the model animal zebrafish in various aspects, including CDS length, exon length, and intron length (Supplementary Fig. S3), indicating that a high-quality protein-coding gene set has been obtained. The distributions of the genomic elements, including the protein-coding genes, tandem repeats (TRs), LTRs, LINEs, SINEs, DNA elements, and GC content, are shown in the circos plot (Fig. 2). Finally, we identified 46 telomeres and 26 centromeres in the chromosome-level genome, indicating that we have successfully obtained a high-quality reference genome for *E. electricus*, with many chromosomes achieving the telomere-to-telomere assembly (Fig. 3).

### Reconstruction the evolutionary histories of the electric eel

**Figure 4:**
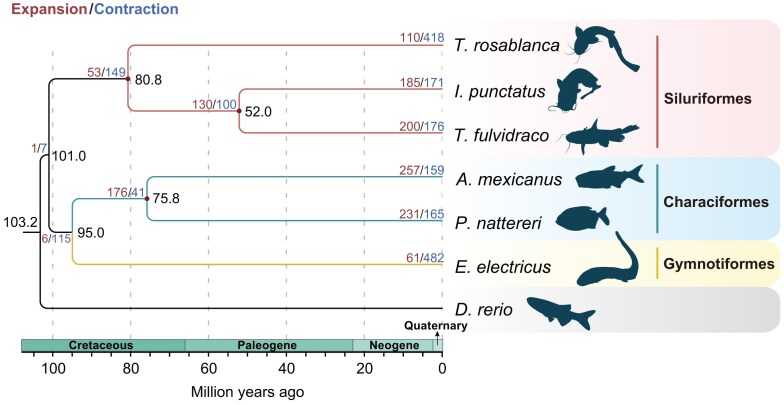
Phylogenetic relationship and divergence time among the 3 orders. The red dots at the nodes represent where fossil records were used for the calibration of divergence time. The black number at each node represents the divergence time between the 2 branches (Mya). The red and blue numbers at each node/species represent the number of expanded and contracted gene families, respectively. The coordinate axis below the phylogenetic tree shows the divergence time scale.

**Figure 5:**
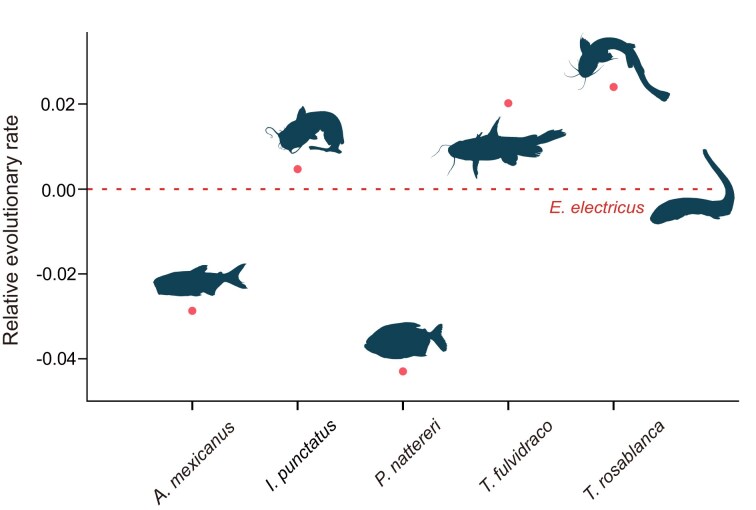
Relative evolutionary rates of species. The analysis was performed using the single-copy orthologous genes with *E. electricus* as the reference species and zebrafish as the outgroup species. The y-axis shows the relative evolutionary rates of the species, and the red dots represent the specific relative evolutionary rates for each species.

**Figure 6:**
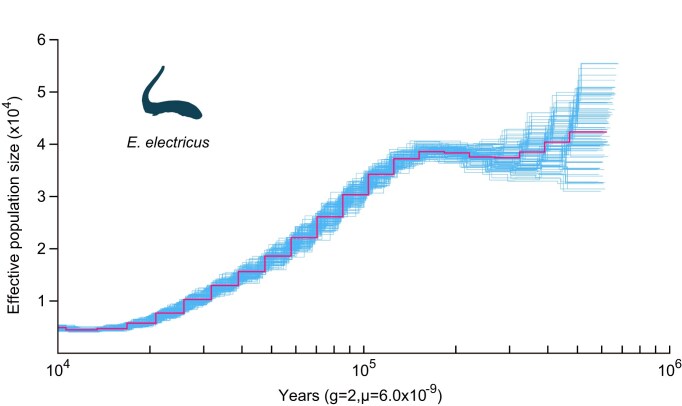
Population history of *E. electricus*. The x-axis represents past years, and the y-axis represents the effective population size of the species; “g” is the generation time, and “μ” is the mutation rate of species.

To enhance our understanding of electric eels, we first conducted a reciprocal BLAST hit analysis utilizing OrthoMCL (v2.0.9) [33], resulting in the identification of 3,548 single-copy orthologous genes shared between the electric eel and 6 other closely related fish species (*D. rerio, I. punctatus, P. nattereri, T. fulvidraco, A. mexicanus*, and *T. rosablanca*), 5 of which belong to the superorder Characiphysae, similar to the electric eel (Supplementary Fig. S4). Previous studies had suggested that Gymnotiformes has a closer relationship with Siluriformes than with Characiformes [1]. However, it remained unclear whether this phylogenetic relationship could be confirmed on a whole-genomic scale analysis. To address this question, we analyzed the phylogenetic relationships among the 7 species, with zebrafish as the outgroup species, using multiple methods, such as the species trees constructed based on different models (homogeneous model, nonhomogeneous model). All results showed that the electric eel was clustered with *A. mexicanus* and *P. nattereri* in 1 branch (Fig. 4; Supplementary Figs. S5 and S6), indicating that Gymnotiformes has a closer relationship with Characiformes than with Siluriformes. Moreover, using the extracted 4-fold degenerate sites from the single-copy orthologous genes, we employed the divergence time analysis and the result showed that the electric eel diverged from the ancestor of *A. mexicanus* and *P. nattereri* approximately 95.00 million years ago (Mya), which falls within the Upper Epoch of Cretaceous period (Fig. 4). Relative evolutionary rate analysis of species showed that the electric eel has a faster evolutionary rate than the 2 Characiformes species (*A. mexicanus* and *P. nattereri*) but slower than the 3 Siluriformes species (*T. rosablanca, T. fulvidraco*, and *I. punctatus*), suggesting that the electric eel faced a relatively strong adaptive pressure compared with the 2 Characiformes species (Fig. 5). Interestingly, we also inferred the effective population size of *E. electricus* and found a sharply decreased trend over the past few hundred thousand years (Fig. 6), which is consistent with the results acquired from 2 additional individuals (Supplementary Fig. S7).

### Gene family expansion contributed to the unique traits of the electric eel

To uncover the potential genetic basis for the unique traits of the electric eel, particularly the evolution of its electric organs, we conducted the gene family analysis. Our analysis identified 61 gene families that have undergone significant expansion in the genome of the electric eel (Fig. 4). Furthermore, we conducted functional enrichment analysis on these expanded gene families, and the results showed that the expanded gene families were primarily involved in the regulations of many core signaling pathways, such as Wnt (positive regulation of canonical Wnt signaling pathway, *P* = 2.96 × 10^−42^; positive regulation of Wnt signaling pathway, *P* = 1.23 × 10^−38^; regulation of canonical Wnt signaling pathway, *P* = 2.15 × 10^−26^), SMO (positive regulation of smoothened signaling pathway, *P* = 1.37 × 10^−63^; smoothened signaling pathway, *P* = 1.59 × 10^−51^; regulation of smoothened signaling pathway, *P* = 5.46 × 10^−46^), and Notch signaling (positive regulation of Notch signaling pathway, *P* = 1.48 × 10^−52^; regulation of Notch signaling pathway, *P* = 2.14 × 10^−32^) (Supplementary Table S9). All these signalings are critical pathways during embryonic/organ development [46–48]. The expansion of regulatory/core genes in these pathways may provide more possibilities to evolve the new organs, especially the 3 electric organs. Interestingly, genes involved in the neurotransmitter catabolic process are also expanded in the *E. electricus* genome (*P* = 1.56 × 10^−2^) (Supplementary Table S9). Previous studies have suggested that when the electric eel is stimulated by neurotransmitters, it will simultaneously open a large number of ion channels, leading to the formation of membrane potential difference and the release of high-voltage electricity [49, 50]. The expansion of regulatory factors for neurotransmitters may significantly contribute to the electric eels’ ability to rapidly respond and discharge high-voltage electricity when they need to attack or defend instantly. Taken together, our results provide insights into the potential genetic underpinnings of the exceptional ability of electric eels to discharge high-voltage electricity.

## Discussion

Electric eels evolved substantial electric organs, enabling them to instantaneously discharge high-voltage electricity for predation, defense, and communication [1, 51]. This study combined PacBio HiFi reads, Nanopore ultra-long reads, Illumina Hi-C reads, and Illumina short-insert sequencing reads to assemble the telomere-to-telomere genome of the electric eel. Compared to the existing electric eel genome on NCBI (GCA_013358815.1), the genome assembly generated in this study has a longer contig N50 and higher BUSCO score than the NCBI version (Supplementary Table S10), indicating that our genome has higher contiguity and completeness. Furthermore, we revealed that the electric eel clusters with *A. mexicanus* and *P. nattereri*, suggesting a closer evolutionary relationship between Gymnotiformes and Characiformes than with Siluriformes, whereas the electric eel diverged from the common ancestor of Characiformes approximately 95.00 Mya. However, the *E. electricus* exhibits a faster evolutionary rate compared to 2 Characiformes species, yet lags behind 3 Siluriformes species, suggesting that the electric eel has faced stronger adaptive pressures than the 2 Characiformes species. Previous studies have rarely focused on the current status of electric eel populations, which is why our knowledge about the size and other information of electric eel populations is very limited. We analyzed the population history of *E. electricus* using the PSMC strategy and found a sharply decreased trend over the past few hundred thousand years, suggesting that the population has been subjected to significant impacts from potential factors in recent years, such as habitat destruction and human interference. Therefore, we hope for the immediate implementation of practical conservation measures. This includes conducting thorough research to understand the causes of the decline in electric eel populations, implementing protection strategies to safeguard their habitats, and raising public awareness about the crucial importance of preserving electric eel populations for biodiversity and ecosystem health. Besides, it is noteworthy that several studies have shown that the expansion of certain key gene families or the increase in gene copy number can significantly enhance specific functions or traits of species [52–54]. The remarkable long-distance electric discharge attack ability evolved by electric eels provides them with an absolute advantage for survival. To investigate whether the copy number of coding genes of electric eels underwent remarkable changes, we analyzed the gene families and found many regulatory factors related to neurotransmitters and classical signaling pathways during embryonic development were significantly expanded, which may contribute to the electric eels’ ability to rapidly respond and discharge high-voltage electricity when they need to attack or defend instantly. Taken together, our study not only produced the first high-quality telomere-to-telomere reference genome for *E. electricus* but also greatly enhanced our understanding of electric eels.

## Supplementary Material

giaf024_Supplemental_File

giaf024_GIGA-D-24-00300_Original_Submission

giaf024_GIGA-D-24-00300_Revision_1

giaf024_GIGA-D-24-00300_Revision_2

giaf024_Response_to_Reviewer_Comments_Original_Submission

giaf024_Response_to_Reviewer_Comments_Revision_1

giaf024_Reviewer_1_Report_Original_Submission Luohao Xu -- 9/7/2024

giaf024_Reviewer_1_Report_Revision_1 Luohao Xu -- 11/29/2024

giaf024_Reviewer_2_Report_Original_Submission Luis Filipe Castro -- 9/12/2024

## Data Availability

Genome assembly and sequencing data (including ONT long reads, HiFi long reads, Hi-C reads, and short-insert reads) have been uploaded to the China National GeneBank database (CNP0005951) and NCBI BioProject under accession number PRJNA1141219. The genome annotation file and additional supporting data are available in the *GigaScience* database, GigaDB [55].

## Additional Files

**Supplementary Table S1**. Statistics of the sequencing data.

**Supplementary Table S2**. Estimation of genome size using GCE software.

**Supplementary Table S3**. Statistics of the intermediate results of genome assemblies.

**Supplementary Table S4**. Final assembly of the *E. electricus* genome.

**Supplementary Table S5**. Mapping ratio of the short reads to the genome assembly.

**Supplementary Table S6**. Statistics of the predicted repetitive sequences in the *E. electricus* genome.

**Supplementary Table S7**. Statistics of transposable elements of the *E. electricus* genome.

**Supplementary Table S8**. Functional annotation of the protein-coding genes in the *E. electricus* genome.

**Supplementary Table S9**. GO enrichment analysis of the expanded gene families.

**Supplementary Table S10**. Comparison of the quality of the 2 genomes.

**Supplementary Fig. S1**. Genomic characteristics of *Electrophorus electricus*. The x-axis shows the *k*-mer depth, and the y-axis shows the frequency of the *k*-mer for a given depth.

**Supplementary Fig. S2**. Sequence mapping during genome gap filling. IGV software was used to display the 10-Kb upstream and downstream regions of the gap in the ONT alignment data. The red triangles represent the position information of the gap.

**Supplementary Fig. S3**. Comparative analysis of CDS, exon, and intron length distributions across species.

**Supplementary Fig. S4**. Identification of orthologous genes among the species.

**Supplementary Fig. S5**. Species tree construction with ASTRAL using RAxML-derived gene trees. The number at each node indicates the local posterior probability.

**Supplementary Fig. S6**. Species tree construction with ASTRAL using IQ-TREE–derived gene trees. The number at each node indicates the local posterior probability.

**Supplementary Fig. S7**. Population history was analyzed using 2 additional individuals of *E. electricus*. The x-axis represents past years, and the y-axis represents the effective population size of the species; “g” is the generation time, and “μ” is the mutation rate of species. (a) The population history was calculated by mapping the sequencing data (SRR1302052) to their corresponding genome assembly. (b) The population history was calculated by mapping the sequencing data (SRR10752306; SRR10752307) to their corresponding genome assembly.

## Abbreviations

BLAST: Basic Local Alignment Search Tool; BUSCO: Benchmarking Universal Single-Copy Orthologs; CDS: coding sequence; EO: electric organ; Gb: gigabase; LINE: long interspersed nuclear element; LTR: long terminal repeat; Mb: megabase; Mya: million years ago; NCBI: National Center for Biotechnology Information; RNA-seq: RNA sequencing; SINE: short interspersed nuclear element; TE: transposable element; TR: tandem repeat.
