# Supplementary material for: Telomere-to-telomere genome assembly of Electrophorus electricus provides insights into the evolution of electric eels
Source: Gigascience. 2025 Apr 1;14:giaf024. doi: 10.1093/gigascience/giaf024 (PMC11959694; doi:10.1093/gigascience/giaf024)
Supplement: giaf024_Supplemental_File [file giaf024_supplemental_file.docx]

**Supplementary Table S1. Statistics of the sequencing data.**

| **Data Type** | **Read Number** | **Base Number** |
| --- | --- | --- |
| Short-insert reads | 323,911,970 | 97,173,591,000 |
| Hi-C reads | 751,954,635 | 225,586,390,500 |
| HiFi reads | 3,512,455 | 59,649,409,097 |
| ONT reads | 582,539 | 46,309 824,226 |

**Supplementary Table S2. Estimation of genome size using GCE software.**

| **K-mer value** | **Raw peak** | **Amount of k-mer used** | **Effective_kmer_individuals** | **Coverage_depth** | **Genome size (Mb)** |
| --- | --- | --- | --- | --- | --- |
| 17 | 83 | 67,805,021,312 | 65,486,236,593 | 83.9 | 780.50 |

**Supplementary Table S3. Statistics of the intermediate results of genome assemblies..**

| **Term** | **HiFiasm** | **Extension** | **Hi-C** |
| --- | --- | --- | --- |
| N50 (bp) | 21,443,492 | 21,439,367 | 29,315,010 |
| N90 (bp) | 2,567,940 | 2,653,473 | 3,000,454 |
| Number of contig | 526 | 479 | 460 |
| Genome size (bp) | 817,931,689 | 823,106,067 | 823,198,452 |
| Max contig length (bp) | 38,564,203 | 40,310,465 | 43,935,756 |
| Average contig length (bp) | 1,555,003 | 1,718,384 | 1,789,561 |

**Supplementary Table S4. Final assembly of the *E. electricus* genome.**

| **Sequence ID** | **Sequence Length (bp)** | **Sequence ID** | **Sequence Length (bp)** |
| --- | --- | --- | --- |
| Chr1 | 45,481,281 | Chr14 | 27,374,293 |
| Chr2 | 44,559,100 | Chr15 | 26,939,823 |
| Chr3 | 41,940,225 | Chr16 | 26,606,700 |
| Chr4 | 40,310,652 | Chr17 | 22,449,376 |
| Chr5 | 38,738,289 | Chr18 | 21,631,257 |
| Chr6 | 37,574,400 | Chr19 | 20,978,735 |
| Chr7 | 36,050,225 | Chr20 | 19,603,054 |
| Chr8 | 35,256,671 | Chr21 | 19,475,296 |
| Chr9 | 33,773,235 | Chr22 | 18,851,105 |
| Chr10 | 33,323,679 | Chr23 | 16,503,649 |
| Chr11 | 30,383,234 | Chr24 | 14,930,753 |
| Chr12 | 29,323,969 | Chr25 | 14,291,220 |
| Chr13 | 27,662,013 | Chr26 | 12,752,543 |
| Total contig length at chromosomes (bp) | | 736,764,777 | |
| Total contig length (bp) | | 833,427,914 | |

**Supplementary Table S5. Mapping ratio of the short reads to the genome assembly.**

| **Total reads** | **Mapped reads** | **Mapping rate (%)** |
| --- | --- | --- |
| 633,474,486 | 632,905,343 | 99.91 |

**Supplementary Table S6. Statistics of the predicted repetitive sequences in *E.electricus* genome.**

| **Method** | **Repeat Size (bp)** | **% of genome** |
| --- | --- | --- |
| Trf | 179,435,911 | 21.53 |
| Repeatmasker | 87,872,071 | 10.54 |
| Proteinmask | 40,672,331 | 4.88 |
| *De novo* | 349,802,642 | 41.91 |
| Total | 396,631,641 | 47.59 |

**Supplementary Table S7. Statistics of transposable elements of *E.electricus* genome.**

| Type | **Combined TEs** | |
| --- | --- | --- |
|  | Length (bp) | % in genome |
| DNA | 100,259,137 | 12.03 |
| LINE | 97,259,132 | 11.67 |
| SINE | 8,218,840 | 0.99 |
| LTR | 38,762,467 | 4.65 |
| Other | 11,521 | 0.00138 |
| Unknown | 192,782,566 | 23.13 |
| Total | 351,796,365 | 42.21 |

**Supplementary Table S8. Functional annotation of the protein-coding genes in *E.electricus* genome.**

| **Term** | **Number** | **Percent(%)** |
| --- | --- | --- |
| InterPro | 19,214 | 91.53 |
| GO | 14,036 | 66.86 |
| KEGG | 15,905 | 75.77 |
| Swissprot | 19,534 | 93.05 |
| TrEMBL | 20,292 | 96.67 |
| Annotated | 20,404 | 97.19 |
| Unanotated | 588 | 2.80 |
| Total | 20,992 | 100% |

**Supplementary Table S9. GO enrichment analysis of the expanded gene families.**

| GO ID | Description | *P*-value |
| --- | --- | --- |
| GO:0048136 | male germ-line cyst formation | 1.43e-131 |
| GO:0016281 | eukaryotic translation initiation factor 4F complex | 5.14e-125 |
| GO:0008190 | eukaryotic initiation factor 4E binding | 6.99e-124 |
| GO:0000340 | RNA 7-methylguanosine cap binding | 4.61e-122 |
| GO:0060903 | positive regulation of meiosis I | 7.02e-120 |
| GO:0000339 | RNA cap binding | 1.75e-116 |
| GO:0060631 | regulation of meiosis I | 1.06e-113 |
| GO:0003938 | IMP dehydrogenase activity | 4.00e-112 |
| GO:1904813 | ficolin-1-rich granule lumen | 2.24e-111 |
| GO:0048137 | spermatocyte division | 5.99e-110 |
| GO:0060041 | retina development in camera-type eye | 1.25e-108 |
| GO:0010803 | regulation of tumor necrosis factor-mediated signaling pathway | 1.37e-106 |
| GO:0045836 | positive regulation of meiotic nuclear division | 6.56e-105 |
| GO:0006183 | GTP biosynthetic process | 8.27e-105 |
| GO:0048134 | germ-line cyst formation | 1.64e-104 |
| GO:0003743 | translation initiation factor activity | 2.03e-103 |
| GO:1901070 | guanosine-containing compound biosynthetic process | 1.23e-102 |
| GO:0031369 | translation initiation factor binding | 3.62e-101 |
| GO:0101002 | ficolin-1-rich granule | 3.16e-100 |
| GO:0051446 | positive regulation of meiotic cell cycle | 6.64e-95 |
| GO:0042451 | purine nucleoside biosynthetic process | 3.36e-94 |
| GO:0046129 | purine ribonucleoside biosynthetic process | 3.36e-94 |
| GO:0042332 | gravitaxis | 4.56e-93 |
| GO:0072577 | endothelial cell apoptotic process | 4.08e-91 |
| GO:0060235 | lens induction in camera-type eye | 2.17e-89 |
| GO:0040020 | regulation of meiotic nuclear division | 2.31e-89 |
| GO:0072331 | signal transduction by p53 class mediator | 7.16e-89 |
| GO:0061072 | iris morphogenesis | 1.95e-88 |
| GO:0006446 | regulation of translational initiation | 9.58e-88 |
| GO:0008135 | translation factor activity, RNA binding | 3.08e-87 |
| GO:0046039 | GTP metabolic process | 7.77e-87 |
| GO:0050821 | protein stabilization | 1.80e-86 |
| GO:0009163 | nucleoside biosynthetic process | 2.99e-86 |
| GO:1901659 | glycosyl compound biosynthetic process | 9.85e-85 |
| GO:0009629 | response to gravity | 9.65e-84 |
| GO:0060059 | embryonic retina morphogenesis in camera-type eye | 8.47e-83 |
| GO:0042771 | intrinsic apoptotic signaling pathway in response to DNA damage by p53 class mediator | 1.49e-82 |
| GO:0046651 | lymphocyte proliferation | 4.85e-80 |
| GO:0001959 | regulation of cytokine-mediated signaling pathway | 7.56e-80 |
| GO:0032943 | mononuclear cell proliferation | 4.90e-79 |
| GO:0006413 | translational initiation | 2.57e-78 |
| GO:0007140 | male meiotic nuclear division | 2.69e-78 |
| GO:0042455 | ribonucleoside biosynthetic process | 3.66e-78 |
| GO:0060759 | regulation of response to cytokine stimulus | 1.47e-77 |
| GO:0035331 | negative regulation of hippo signaling | 1.64e-77 |
| GO:0070661 | leukocyte proliferation | 4.03e-77 |
| GO:0051445 | regulation of meiotic cell cycle | 1.71e-76 |
| GO:0010842 | retina layer formation | 1.45e-74 |
| GO:1901068 | guanosine-containing compound metabolic process | 1.81e-73 |
| GO:0002089 | lens morphogenesis in camera-type eye | 4.04e-73 |
| GO:1904019 | epithelial cell apoptotic process | 9.94e-73 |
| GO:0051785 | positive regulation of nuclear division | 1.63e-72 |
| GO:0072332 | intrinsic apoptotic signaling pathway by p53 class mediator | 5.89e-72 |
| GO:0009408 | response to heat | 6.38e-72 |
| GO:0035578 | azurophil granule lumen | 1.15e-70 |
| GO:0009205 | purine ribonucleoside triphosphate metabolic process | 5.01e-69 |
| GO:0051783 | regulation of nuclear division | 7.03e-69 |
| GO:0009199 | ribonucleoside triphosphate metabolic process | 1.31e-68 |
| GO:0060216 | definitive hemopoiesis | 2.48e-68 |
| GO:0009144 | purine nucleoside triphosphate metabolic process | 6.35e-68 |
| GO:0031128 | developmental induction | 1.11e-67 |
| GO:0035330 | regulation of hippo signaling | 6.05e-67 |
| GO:0060205 | cytoplasmic vesicle lumen | 1.04e-66 |
| GO:0009409 | response to cold | 2.76e-66 |
| GO:0034774 | secretory granule lumen | 3.28e-66 |
| GO:0031983 | vesicle lumen | 6.77e-66 |
| GO:0009167 | purine ribonucleoside monophosphate metabolic process | 8.25e-66 |
| GO:0009119 | ribonucleoside metabolic process | 8.56e-66 |
| GO:0016616 | oxidoreductase activity, acting on the CH-OH group of donors, NAD or NADP as acceptor | 1.43e-65 |
| GO:0009126 | purine nucleoside monophosphate metabolic process | 1.49e-65 |
| GO:0045727 | positive regulation of translation | 1.89e-65 |
| GO:0009141 | nucleoside triphosphate metabolic process | 1.99e-65 |
| GO:0046128 | purine ribonucleoside metabolic process | 1.18e-64 |
| GO:0000045 | autophagosome assembly | 3.62e-64 |
| GO:0016614 | oxidoreductase activity, acting on CH-OH group of donors | 4.43e-64 |
| GO:0042278 | purine nucleoside metabolic process | 6.24e-64 |
| GO:0009161 | ribonucleoside monophosphate metabolic process | 8.26e-64 |
| GO:0045880 | positive regulation of smoothened signaling pathway | 1.37e-63 |
| GO:0009206 | purine ribonucleoside triphosphate biosynthetic process | 3.17e-63 |
| GO:0008630 | intrinsic apoptotic signaling pathway in response to DNA damage | 8.26e-63 |
| GO:0009145 | purine nucleoside triphosphate biosynthetic process | 9.18e-63 |
| GO:0048596 | embryonic camera-type eye morphogenesis | 1.35e-62 |
| GO:0009201 | ribonucleoside triphosphate biosynthetic process | 1.55e-62 |
| GO:1905037 | autophagosome organization | 1.81e-62 |
| GO:0009116 | nucleoside metabolic process | 8.56e-62 |
| GO:0009123 | nucleoside monophosphate metabolic process | 2.45e-61 |
| GO:0043388 | positive regulation of DNA binding | 5.28e-61 |
| GO:0009142 | nucleoside triphosphate biosynthetic process | 1.59e-59 |
| GO:0009127 | purine nucleoside monophosphate biosynthetic process | 4.05e-59 |
| GO:0009168 | purine ribonucleoside monophosphate biosynthetic process | 4.05e-59 |
| GO:2000243 | positive regulation of reproductive process | 4.11e-59 |
| GO:0046622 | positive regulation of organ growth | 4.26e-59 |
| GO:0016605 | PML body | 6.08e-59 |
| GO:0034250 | positive regulation of cellular amide metabolic process | 2.00e-58 |
| GO:0005775 | vacuolar lumen | 2.42e-58 |
| GO:0034404 | nucleobase-containing small molecule biosynthetic process | 3.45e-58 |
| GO:0031076 | embryonic camera-type eye development | 4.11e-58 |
| GO:0048048 | embryonic eye morphogenesis | 7.41e-58 |
| GO:1901657 | glycosyl compound metabolic process | 8.81e-58 |
| GO:0003714 | transcription corepressor activity | 9.72e-58 |
| GO:0008586 | imaginal disc-derived wing vein morphogenesis | 1.72e-57 |
| GO:0034333 | adherens junction assembly | 6.14e-57 |
| GO:0005766 | primary lysosome | 1.62e-56 |
| GO:0042582 | azurophil granule | 1.62e-56 |
| GO:0009156 | ribonucleoside monophosphate biosynthetic process | 1.95e-56 |
| GO:0046649 | lymphocyte activation | 3.00e-56 |
| GO:0009124 | nucleoside monophosphate biosynthetic process | 5.17e-55 |
| GO:0048515 | spermatid differentiation | 1.56e-54 |
| GO:0097193 | intrinsic apoptotic signaling pathway | 3.07e-54 |
| GO:0008013 | beta-catenin binding | 1.05e-53 |
| GO:0060042 | retina morphogenesis in camera-type eye | 3.12e-53 |
| GO:0008587 | imaginal disc-derived wing margin morphogenesis | 9.62e-53 |
| GO:0045747 | positive regulation of Notch signaling pathway | 1.48e-52 |
| GO:0002088 | lens development in camera-type eye | 2.11e-52 |
| GO:0048066 | developmental pigmentation | 3.93e-52 |
| GO:0003407 | neural retina development | 5.36e-52 |
| GO:0007224 | smoothened signaling pathway | 1.59e-51 |
| GO:0140013 | meiotic nuclear division | 1.61e-51 |
| GO:0097191 | extrinsic apoptotic signaling pathway | 6.71e-51 |
| GO:0090068 | positive regulation of cell cycle process | 3.09e-50 |
| GO:1903046 | meiotic cell cycle process | 1.72e-49 |
| GO:0016607 | nuclear speck | 5.70e-48 |
| GO:1901796 | regulation of signal transduction by p53 class mediator | 9.81e-48 |
| GO:0051321 | meiotic cell cycle | 3.05e-47 |
| GO:0007033 | vacuole organization | 1.05e-46 |
| GO:0051101 | regulation of DNA binding | 1.47e-46 |
| GO:0008589 | regulation of smoothened signaling pathway | 5.46e-46 |
| GO:0006412 | translation | 9.71e-46 |
| GO:0043043 | peptide biosynthetic process | 5.37e-44 |
| GO:0009152 | purine ribonucleotide biosynthetic process | 8.37e-44 |
| GO:0043473 | pigmentation | 2.43e-43 |
| GO:0006164 | purine nucleotide biosynthetic process | 3.07e-43 |
| GO:0002020 | protease binding | 6.75e-43 |
| GO:0016236 | macroautophagy | 9.35e-43 |
| GO:0034332 | adherens junction organization | 2.47e-42 |
| GO:0090263 | positive regulation of canonical Wnt signaling pathway | 2.96e-42 |
| GO:0055131 | C3HC4-type RING finger domain binding | 4.57e-42 |
| GO:0072522 | purine-containing compound biosynthetic process | 4.88e-42 |
| GO:0009260 | ribonucleotide biosynthetic process | 6.24e-42 |
| GO:0046390 | ribose phosphate biosynthetic process | 1.02e-41 |
| GO:0031249 | denatured protein binding | 2.66e-40 |
| GO:1902380 | positive regulation of endoribonuclease activity | 2.66e-40 |
| GO:0045766 | positive regulation of angiogenesis | 4.01e-40 |
| GO:0046620 | regulation of organ growth | 1.75e-39 |
| GO:0032287 | peripheral nervous system myelin maintenance | 3.28e-39 |
| GO:0097190 | apoptotic signaling pathway | 4.93e-39 |
| GO:0070370 | cellular heat acclimation | 7.22e-39 |
| GO:0070426 | positive regulation of nucleotide-binding oligomerization domain containing signaling pathway | 7.22e-39 |
| GO:0070434 | positive regulation of nucleotide-binding oligomerization domain containing 2 signaling pathway | 7.22e-39 |
| GO:0030177 | positive regulation of Wnt signaling pathway | 1.23e-38 |
| GO:0051099 | positive regulation of binding | 1.82e-38 |
| GO:0031116 | positive regulation of microtubule polymerization | 2.83e-38 |
| GO:0048593 | camera-type eye morphogenesis | 4.55e-38 |
| GO:1902946 | protein localization to early endosome | 6.99e-38 |
| GO:0006914 | autophagy | 6.99e-38 |
| GO:0061919 | process utilizing autophagic mechanism | 6.99e-38 |
| GO:1904018 | positive regulation of vasculature development | 1.50e-37 |
| GO:0010951 | negative regulation of endopeptidase activity | 5.79e-37 |
| GO:0031112 | positive regulation of microtubule polymerization or depolymerization | 8.24e-37 |
| GO:0060699 | regulation of endoribonuclease activity | 9.80e-37 |
| GO:0060700 | regulation of ribonuclease activity | 9.80e-37 |
| GO:0010466 | negative regulation of peptidase activity | 1.33e-36 |
| GO:0004867 | serine-type endopeptidase inhibitor activity | 5.54e-36 |
| GO:0035696 | monocyte extravasation | 7.09e-36 |
| GO:0070432 | regulation of nucleotide-binding oligomerization domain containing 2 signaling pathway | 7.35e-36 |
| GO:0090063 | positive regulation of microtubule nucleation | 4.55e-35 |
| GO:1903955 | positive regulation of protein targeting to mitochondrion | 4.55e-35 |
| GO:0043217 | myelin maintenance | 1.02e-34 |
| GO:0009165 | nucleotide biosynthetic process | 1.53e-34 |
| GO:0042026 | protein refolding | 2.35e-34 |
| GO:0070424 | regulation of nucleotide-binding oligomerization domain containing signaling pathway | 2.42e-34 |
| GO:1901293 | nucleoside phosphate biosynthetic process | 3.75e-34 |
| GO:0051131 | chaperone-mediated protein complex assembly | 4.77e-34 |
| GO:0045576 | mast cell activation | 1.73e-33 |
| GO:0090084 | negative regulation of inclusion body assembly | 4.82e-33 |
| GO:1903265 | positive regulation of tumor necrosis factor-mediated signaling pathway | 4.82e-33 |
| GO:0045296 | cadherin binding | 6.72e-33 |
| GO:0002199 | zona pellucida receptor complex | 1.87e-32 |
| GO:0008593 | regulation of Notch signaling pathway | 2.14e-32 |
| GO:0032075 | positive regulation of nuclease activity | 6.69e-32 |
| GO:0031113 | regulation of microtubule polymerization | 1.04e-31 |
| GO:0036010 | protein localization to endosome | 1.13e-31 |
| GO:0072672 | neutrophil extravasation | 1.27e-31 |
| GO:0022011 | myelination in peripheral nervous system | 3.71e-31 |
| GO:0032292 | peripheral nervous system axon ensheathment | 3.71e-31 |
| GO:0032757 | positive regulation of interleukin-8 production | 3.98e-31 |
| GO:1901029 | negative regulation of mitochondrial outer membrane permeabilization involved in apoptotic signaling pathway | 7.03e-31 |
| GO:0046629 | gamma-delta T cell activation | 7.15e-31 |
| GO:0010968 | regulation of microtubule nucleation | 2.09e-30 |
| GO:0045765 | regulation of angiogenesis | 2.11e-30 |
| GO:0034329 | cell junction assembly | 2.44e-30 |
| GO:0032558 | adenyl deoxyribonucleotide binding | 3.25e-30 |
| GO:0032564 | dATP binding | 3.25e-30 |
| GO:0046579 | positive regulation of Ras protein signal transduction | 7.92e-30 |
| GO:0014044 | Schwann cell development | 1.45e-29 |
| GO:0043015 | gamma-tubulin binding | 1.45e-29 |
| GO:0042826 | histone deacetylase binding | 1.85e-29 |
| GO:0097201 | negative regulation of transcription from RNA polymerase II promoter in response to stress | 4.18e-29 |
| GO:1903214 | regulation of protein targeting to mitochondrion | 4.18e-29 |
| GO:0051057 | positive regulation of small GTPase mediated signal transduction | 7.10e-29 |
| GO:0032554 | purine deoxyribonucleotide binding | 8.24e-29 |
| GO:0098532 | histone H3-K27 trimethylation | 8.24e-29 |
| GO:0090083 | regulation of inclusion body assembly | 1.05e-28 |
| GO:1902236 | negative regulation of endoplasmic reticulum stress-induced intrinsic apoptotic signaling pathway | 1.75e-28 |
| GO:0071169 | establishment of protein localization to chromatin | 3.42e-28 |
| GO:0032677 | regulation of interleukin-8 production | 5.25e-28 |
| GO:0032069 | regulation of nuclease activity | 5.94e-28 |
| GO:1901342 | regulation of vasculature development | 1.27e-27 |
| GO:0016584 | nucleosome positioning | 1.28e-27 |
| GO:0032552 | deoxyribonucleotide binding | 1.28e-27 |
| GO:0031101 | fin regeneration | 1.78e-27 |
| GO:0014037 | Schwann cell differentiation | 3.76e-27 |
| GO:0045861 | negative regulation of proteolysis | 4.55e-27 |
| GO:0034014 | response to triglyceride | 4.79e-27 |
| GO:0060054 | positive regulation of epithelial cell proliferation involved in wound healing | 5.99e-27 |
| GO:1901673 | regulation of mitotic spindle assembly | 6.45e-27 |
| GO:0000788 | nuclear nucleosome | 1.41e-26 |
| GO:0070199 | establishment of protein localization to chromosome | 1.41e-26 |
| GO:1903533 | regulation of protein targeting | 1.56e-26 |
| GO:0050839 | cell adhesion molecule binding | 1.68e-26 |
| GO:0060828 | regulation of canonical Wnt signaling pathway | 2.15e-26 |
| GO:0007339 | binding of sperm to zona pellucida | 2.79e-26 |
| GO:0044183 | protein folding chaperone | 5.61e-26 |
| GO:0033120 | positive regulation of RNA splicing | 7.92e-26 |
| GO:0033986 | response to methanol | 8.81e-26 |
| GO:1900003 | regulation of serine-type endopeptidase activity | 8.81e-26 |
| GO:1900004 | negative regulation of serine-type endopeptidase activity | 8.81e-26 |
| GO:1902571 | regulation of serine-type peptidase activity | 8.81e-26 |
| GO:1902572 | negative regulation of serine-type peptidase activity | 8.81e-26 |
| GO:0010286 | heat acclimation | 1.11e-25 |
| GO:0070734 | histone H3-K27 methylation | 1.19e-25 |
| GO:0052548 | regulation of endopeptidase activity | 2.04e-25 |
| GO:0035036 | sperm-egg recognition | 2.14e-25 |
| GO:0045216 | cell-cell junction organization | 2.74e-25 |
| GO:1900034 | regulation of cellular response to heat | 5.97e-25 |
| GO:0046687 | response to chromate | 8.53e-25 |
| GO:0031110 | regulation of microtubule polymerization or depolymerization | 9.25e-25 |
| GO:1902235 | regulation of endoplasmic reticulum stress-induced intrinsic apoptotic signaling pathway | 1.78e-24 |
| GO:0045721 | negative regulation of gluconeogenesis | 3.89e-24 |
| GO:1901099 | negative regulation of signal transduction in absence of ligand | 4.53e-24 |
| GO:2001240 | negative regulation of extrinsic apoptotic signaling pathway in absence of ligand | 4.53e-24 |
| GO:0051574 | positive regulation of histone H3-K9 methylation | 4.71e-24 |
| GO:0032273 | positive regulation of protein polymerization | 7.78e-24 |
| GO:0051059 | NF-kappaB binding | 7.96e-24 |
| GO:0080182 | histone H3-K4 trimethylation | 1.07e-23 |
| GO:0051346 | negative regulation of hydrolase activity | 1.40e-23 |
| GO:1903573 | negative regulation of response to endoplasmic reticulum stress | 2.13e-23 |
| GO:0052547 | regulation of peptidase activity | 2.52e-23 |
| GO:0016235 | aggresome | 3.90e-23 |
| GO:0090169 | regulation of spindle assembly | 4.02e-23 |
| GO:1903749 | positive regulation of establishment of protein localization to mitochondrion | 4.02e-23 |
| GO:0016208 | AMP binding | 5.06e-23 |
| GO:0048793 | pronephros development | 9.10e-23 |
| GO:0004866 | endopeptidase inhibitor activity | 1.91e-22 |
| GO:0034330 | cell junction organization | 2.21e-22 |
| GO:0051082 | unfolded protein binding | 2.26e-22 |
| GO:0030414 | peptidase inhibitor activity | 2.65e-22 |
| GO:0045648 | positive regulation of erythrocyte differentiation | 6.01e-22 |
| GO:0001961 | positive regulation of cytokine-mediated signaling pathway | 1.19e-21 |
| GO:1903747 | regulation of establishment of protein localization to mitochondrion | 1.40e-21 |
| GO:0061135 | endopeptidase regulator activity | 1.75e-21 |
| GO:0055038 | recycling endosome membrane | 2.28e-21 |
| GO:0061134 | peptidase regulator activity | 2.69e-21 |
| GO:0009988 | cell-cell recognition | 2.87e-21 |
| GO:0002931 | response to ischemia | 4.72e-21 |
| GO:0097718 | disordered domain specific binding | 1.02e-20 |
| GO:0060760 | positive regulation of response to cytokine stimulus | 1.53e-20 |
| GO:1901028 | regulation of mitochondrial outer membrane permeabilization involved in apoptotic signaling pathway | 2.27e-20 |
| GO:0030330 | DNA damage response, signal transduction by p53 class mediator | 2.71e-20 |
| GO:0045123 | cellular extravasation | 2.99e-20 |
| GO:0005524 | ATP binding | 3.00e-20 |
| GO:2001239 | regulation of extrinsic apoptotic signaling pathway in absence of ligand | 4.95e-20 |
| GO:0034605 | cellular response to heat | 7.57e-20 |
| GO:0031072 | heat shock protein binding | 7.78e-20 |
| GO:0051570 | regulation of histone H3-K9 methylation | 8.31e-20 |
| GO:0071168 | protein localization to chromatin | 1.38e-19 |
| GO:0006986 | response to unfolded protein | 2.03e-19 |
| GO:0010822 | positive regulation of mitochondrion organization | 2.52e-19 |
| GO:0018023 | peptidyl-lysine trimethylation | 3.68e-19 |
| GO:0040036 | regulation of fibroblast growth factor receptor signaling pathway | 4.90e-19 |
| GO:0045646 | regulation of erythrocyte differentiation | 4.90e-19 |
| GO:0071674 | mononuclear cell migration | 7.37e-19 |
| GO:0060236 | regulation of mitotic spindle organization | 8.70e-19 |
| GO:0043531 | ADP binding | 1.49e-18 |
| GO:0007375 | anterior midgut invagination | 3.03e-18 |
| GO:0016534 | NA | 3.03e-18 |
| GO:0035966 | response to topologically incorrect protein | 4.77e-18 |
| GO:1902905 | positive regulation of supramolecular fiber organization | 5.18e-18 |
| GO:0010823 | negative regulation of mitochondrion organization | 8.14e-18 |
| GO:0042552 | myelination | 9.80e-18 |
| GO:0090559 | regulation of membrane permeability | 1.46e-17 |
| GO:0042770 | signal transduction in response to DNA damage | 1.92e-17 |
| GO:0007009 | plasma membrane organization | 2.20e-17 |
| GO:0005719 | nuclear euchromatin | 2.65e-17 |
| GO:0032271 | regulation of protein polymerization | 2.70e-17 |
| GO:0016234 | inclusion body | 3.02e-17 |
| GO:1905897 | regulation of response to endoplasmic reticulum stress | 4.03e-17 |
| GO:0090224 | regulation of spindle organization | 4.69e-17 |
| GO:0051092 | positive regulation of NF-kappaB transcription factor activity | 5.29e-17 |
| GO:0048260 | positive regulation of receptor-mediated endocytosis | 7.78e-17 |
| GO:0051568 | histone H3-K4 methylation | 8.12e-17 |
| GO:0043618 | regulation of transcription from RNA polymerase II promoter in response to stress | 8.62e-17 |
| GO:0021586 | pons maturation | 8.83e-17 |
| GO:0021718 | superior olivary nucleus development | 8.83e-17 |
| GO:0021722 | superior olivary nucleus maturation | 8.83e-17 |
| GO:0030512 | negative regulation of transforming growth factor beta receptor signaling pathway | 1.11e-16 |
| GO:0051495 | positive regulation of cytoskeleton organization | 1.26e-16 |
| GO:1903845 | negative regulation of cellular response to transforming growth factor beta stimulus | 1.84e-16 |
| GO:0030593 | neutrophil chemotaxis | 2.53e-16 |
| GO:0035327 | transcriptionally active chromatin | 3.28e-16 |
| GO:0046902 | regulation of mitochondrial membrane permeability | 3.95e-16 |
| GO:0005720 | nuclear heterochromatin | 4.58e-16 |
| GO:0000786 | nucleosome | 6.35e-16 |
| GO:0031334 | positive regulation of protein-containing complex assembly | 6.71e-16 |
| GO:0001520 | outer dense fiber | 7.58e-16 |
| GO:0016176 | superoxide-generating NADPH oxidase activator activity | 7.58e-16 |
| GO:0031062 | positive regulation of histone methylation | 8.77e-16 |
| GO:0042246 | tissue regeneration | 1.06e-15 |
| GO:0008366 | axon ensheathment | 1.12e-15 |
| GO:0007157 | heterophilic cell-cell adhesion via plasma membrane cell adhesion molecules | 1.16e-15 |
| GO:0043620 | regulation of DNA-templated transcription in response to stress | 1.53e-15 |
| GO:0043154 | negative regulation of cysteine-type endopeptidase activity involved in apoptotic process | 1.60e-15 |
| GO:0007272 | ensheathment of neurons | 1.95e-15 |
| GO:1990266 | neutrophil migration | 3.60e-15 |
| GO:0046578 | regulation of Ras protein signal transduction | 3.68e-15 |
| GO:0030308 | negative regulation of cell growth | 4.17e-15 |
| GO:2000117 | negative regulation of cysteine-type endopeptidase activity | 4.28e-15 |
| GO:0043488 | regulation of mRNA stability | 4.76e-15 |
| GO:0031093 | platelet alpha granule lumen | 6.19e-15 |
| GO:0021782 | glial cell development | 6.54e-15 |
| GO:0044815 | DNA packaging complex | 7.24e-15 |
| GO:0005913 | NA | 7.98e-15 |
| GO:0071621 | granulocyte chemotaxis | 8.07e-15 |
| GO:0090316 | positive regulation of intracellular protein transport | 8.81e-15 |
| GO:0000791 | euchromatin | 1.27e-14 |
| GO:0031397 | negative regulation of protein ubiquitination | 1.60e-14 |
| GO:2001243 | negative regulation of intrinsic apoptotic signaling pathway | 1.79e-14 |
| GO:0000723 | telomere maintenance | 1.98e-14 |
| GO:0032200 | telomere organization | 2.44e-14 |
| GO:0043487 | regulation of RNA stability | 2.57e-14 |
| GO:0016533 | protein kinase 5 complex | 2.73e-14 |
| GO:0045807 | positive regulation of endocytosis | 3.95e-14 |
| GO:0006457 | protein folding | 4.39e-14 |
| GO:0007374 | posterior midgut invagination | 4.64e-14 |
| GO:0005814 | centriole | 6.69e-14 |
| GO:0045639 | positive regulation of myeloid cell differentiation | 7.41e-14 |
| GO:0001664 | G protein-coupled receptor binding | 7.82e-14 |
| GO:2001236 | regulation of extrinsic apoptotic signaling pathway | 8.73e-14 |
| GO:2001237 | negative regulation of extrinsic apoptotic signaling pathway | 8.92e-14 |
| GO:0048259 | regulation of receptor-mediated endocytosis | 9.06e-14 |
| GO:0010677 | negative regulation of cellular carbohydrate metabolic process | 9.40e-14 |
| GO:0046628 | positive regulation of insulin receptor signaling pathway | 9.88e-14 |
| GO:0097530 | granulocyte migration | 1.20e-13 |
| GO:1903321 | negative regulation of protein modification by small protein conjugation or removal | 1.29e-13 |
| GO:0043484 | regulation of RNA splicing | 1.56e-13 |
| GO:0031091 | platelet alpha granule | 1.59e-13 |
| GO:0006111 | regulation of gluconeogenesis | 1.74e-13 |
| GO:2001242 | regulation of intrinsic apoptotic signaling pathway | 2.23e-13 |
| GO:0061013 | regulation of mRNA catabolic process | 2.24e-13 |
| GO:0010821 | regulation of mitochondrion organization | 2.54e-13 |
| GO:0070507 | regulation of microtubule cytoskeleton organization | 5.55e-13 |
| GO:0071353 | cellular response to interleukin-4 | 5.57e-13 |
| GO:0045912 | negative regulation of carbohydrate metabolic process | 7.32e-13 |
| GO:0010288 | response to lead ion | 8.14e-13 |
| GO:0033116 | endoplasmic reticulum-Golgi intermediate compartment membrane | 8.14e-13 |
| GO:0017015 | regulation of transforming growth factor beta receptor signaling pathway | 1.09e-12 |
| GO:0051056 | regulation of small GTPase mediated signal transduction | 1.24e-12 |
| GO:0034620 | cellular response to unfolded protein | 1.27e-12 |
| GO:1903844 | regulation of cellular response to transforming growth factor beta stimulus | 1.48e-12 |
| GO:0007006 | mitochondrial membrane organization | 1.72e-12 |
| GO:0034502 | protein localization to chromosome | 1.93e-12 |
| GO:0035967 | cellular response to topologically incorrect protein | 1.94e-12 |
| GO:1900078 | positive regulation of cellular response to insulin stimulus | 1.95e-12 |
| GO:0099513 | polymeric cytoskeletal fiber | 2.18e-12 |
| GO:0043020 | NADPH oxidase complex | 2.25e-12 |
| GO:0030478 | actin cap | 2.88e-12 |
| GO:0043209 | myelin sheath | 4.49e-12 |
| GO:0034968 | histone lysine methylation | 5.30e-12 |
| GO:0032436 | positive regulation of proteasomal ubiquitin-dependent protein catabolic process | 5.39e-12 |
| GO:0032886 | regulation of microtubule-based process | 7.49e-12 |
| GO:0031060 | regulation of histone methylation | 7.81e-12 |
| GO:0071456 | cellular response to hypoxia | 7.96e-12 |
| GO:0051055 | negative regulation of lipid biosynthetic process | 9.23e-12 |
| GO:0090207 | regulation of triglyceride metabolic process | 9.23e-12 |
| GO:0015631 | tubulin binding | 1.09e-11 |
| GO:0021578 | hindbrain maturation | 1.11e-11 |
| GO:0021626 | central nervous system maturation | 1.11e-11 |
| GO:2000060 | positive regulation of ubiquitin-dependent protein catabolic process | 1.16e-11 |
| GO:0030261 | chromosome condensation | 1.37e-11 |
| GO:0042501 | serine phosphorylation of STAT protein | 1.49e-11 |
| GO:0097529 | myeloid leukocyte migration | 2.38e-11 |
| GO:0090101 | negative regulation of transmembrane receptor protein serine/threonine kinase signaling pathway | 2.87e-11 |
| GO:0043519 | regulation of myosin II filament organization | 3.25e-11 |
| GO:0007338 | single fertilization | 3.71e-11 |
| GO:0036294 | cellular response to decreased oxygen levels | 3.75e-11 |
| GO:0042063 | gliogenesis | 4.31e-11 |
| GO:0021819 | layer formation in cerebral cortex | 4.38e-11 |
| GO:0005874 | microtubule | 4.88e-11 |
| GO:0007422 | peripheral nervous system development | 8.75e-11 |
| GO:0003381 | epithelial cell morphogenesis involved in gastrulation | 9.11e-11 |
| GO:0003384 | apical constriction involved in gastrulation | 9.11e-11 |
| GO:0005826 | actomyosin contractile ring | 9.11e-11 |
| GO:0043281 | regulation of cysteine-type endopeptidase activity involved in apoptotic process | 1.06e-10 |
| GO:0016571 | histone methylation | 1.10e-10 |
| GO:0031490 | chromatin DNA binding | 1.10e-10 |
| GO:0018022 | peptidyl-lysine methylation | 1.30e-10 |
| GO:0055037 | recycling endosome | 1.46e-10 |
| GO:0007377 | germ-band extension | 1.47e-10 |
| GO:0090254 | cell elongation involved in imaginal disc-derived wing morphogenesis | 1.47e-10 |
| GO:0070670 | response to interleukin-4 | 1.64e-10 |
| GO:0048207 | vesicle targeting, rough ER to cis-Golgi | 1.80e-10 |
| GO:0048208 | COPII vesicle coating | 1.80e-10 |
| GO:0090181 | regulation of cholesterol metabolic process | 1.80e-10 |
| GO:0047485 | protein N-terminus binding | 1.86e-10 |
| GO:1901800 | positive regulation of proteasomal protein catabolic process | 1.86e-10 |
| GO:0030595 | leukocyte chemotaxis | 2.16e-10 |
| GO:0071453 | cellular response to oxygen levels | 2.84e-10 |
| GO:0046104 | thymidine metabolic process | 3.24e-10 |
| GO:0006901 | vesicle coating | 3.48e-10 |
| GO:0044450 | NA | 3.50e-10 |
| GO:0032434 | regulation of proteasomal ubiquitin-dependent protein catabolic process | 5.04e-10 |
| GO:0014068 | positive regulation of phosphatidylinositol 3-kinase signaling | 5.40e-10 |
| GO:0000792 | heterochromatin | 6.02e-10 |
| GO:0030134 | COPII-coated ER to Golgi transport vesicle | 6.50e-10 |
| GO:0005793 | endoplasmic reticulum-Golgi intermediate compartment | 7.43e-10 |
| GO:0045089 | positive regulation of innate immune response | 7.82e-10 |
| GO:0048199 | vesicle targeting, to, from or within Golgi | 7.96e-10 |
| GO:0090288 | negative regulation of cellular response to growth factor stimulus | 8.03e-10 |
| GO:0007370 | ventral furrow formation | 8.20e-10 |
| GO:0002576 | platelet degranulation | 8.87e-10 |
| GO:0005923 | bicellular tight junction | 8.87e-10 |
| GO:0006402 | mRNA catabolic process | 8.90e-10 |
| GO:0006212 | uracil catabolic process | 9.42e-10 |
| GO:0090114 | COPII-coated vesicle budding | 9.71e-10 |
| GO:0008037 | cell recognition | 1.08e-09 |
| GO:2000116 | regulation of cysteine-type endopeptidase activity | 1.08e-09 |
| GO:0030589 | pseudocleavage involved in syncytial blastoderm formation | 1.21e-09 |
| GO:0032388 | positive regulation of intracellular transport | 1.28e-09 |
| GO:0051091 | positive regulation of DNA-binding transcription factor activity | 1.28e-09 |
| GO:0009566 | fertilization | 1.45e-09 |
| GO:0031497 | chromatin assembly | 1.85e-09 |
| GO:0001819 | positive regulation of cytokine production | 1.91e-09 |
| GO:0033157 | regulation of intracellular protein transport | 2.09e-09 |
| GO:1903052 | positive regulation of proteolysis involved in cellular protein catabolic process | 2.27e-09 |
| GO:0006208 | pyrimidine nucleobase catabolic process | 2.37e-09 |
| GO:0019860 | uracil metabolic process | 2.37e-09 |
| GO:0031058 | positive regulation of histone modification | 2.43e-09 |
| GO:0015174 | basic amino acid transmembrane transporter activity | 2.73e-09 |
| GO:1990822 | basic amino acid transmembrane transport | 2.73e-09 |
| GO:0017137 | Rab GTPase binding | 3.04e-09 |
| GO:2000058 | regulation of ubiquitin-dependent protein catabolic process | 3.09e-09 |
| GO:0060263 | regulation of respiratory burst | 3.23e-09 |
| GO:0021548 | pons development | 3.95e-09 |
| GO:0004857 | enzyme inhibitor activity | 3.96e-09 |
| GO:0010001 | glial cell differentiation | 4.28e-09 |
| GO:0015802 | basic amino acid transport | 5.02e-09 |
| GO:0048194 | Golgi vesicle budding | 6.12e-09 |
| GO:0062014 | negative regulation of small molecule metabolic process | 6.18e-09 |
| GO:0046034 | ATP metabolic process | 6.28e-09 |
| GO:0006801 | superoxide metabolic process | 6.89e-09 |
| GO:0043255 | regulation of carbohydrate biosynthetic process | 7.04e-09 |
| GO:0030100 | regulation of endocytosis | 7.35e-09 |
| GO:0021801 | cerebral cortex radial glia guided migration | 8.46e-09 |
| GO:0022030 | telencephalon glial cell migration | 8.46e-09 |
| GO:0045088 | regulation of innate immune response | 9.00e-09 |
| GO:0006401 | RNA catabolic process | 1.18e-08 |
| GO:0009226 | nucleotide-sugar biosynthetic process | 1.21e-08 |
| GO:0010310 | regulation of hydrogen peroxide metabolic process | 1.26e-08 |
| GO:0008017 | microtubule binding | 1.26e-08 |
| GO:0030588 | pseudocleavage | 1.28e-08 |
| GO:0061702 | inflammasome complex | 1.82e-08 |
| GO:0005089 | Rho guanyl-nucleotide exchange factor activity | 2.05e-08 |
| GO:0005178 | integrin binding | 2.08e-08 |
| GO:0046108 | uridine metabolic process | 2.16e-08 |
| GO:1903708 | positive regulation of hemopoiesis | 2.22e-08 |
| GO:0007277 | pole cell development | 2.29e-08 |
| GO:0070938 | contractile ring | 2.29e-08 |
| GO:0042110 | T cell activation | 2.57e-08 |
| GO:0006333 | chromatin assembly or disassembly | 3.90e-08 |
| GO:0046113 | nucleobase catabolic process | 3.96e-08 |
| GO:0006487 | protein N-linked glycosylation | 4.12e-08 |
| GO:0045833 | negative regulation of lipid metabolic process | 4.28e-08 |
| GO:0003383 | apical constriction | 6.66e-08 |
| GO:0014066 | regulation of phosphatidylinositol 3-kinase signaling | 6.93e-08 |
| GO:1903364 | positive regulation of cellular protein catabolic process | 7.12e-08 |
| GO:0032993 | protein-DNA complex | 8.22e-08 |
| GO:1905269 | positive regulation of chromatin organization | 8.22e-08 |
| GO:0006479 | protein methylation | 9.11e-08 |
| GO:0008213 | protein alkylation | 9.11e-08 |
| GO:0043539 | protein serine/threonine kinase activator activity | 1.09e-07 |
| GO:0050691 | regulation of defense response to virus by host | 1.09e-07 |
| GO:0098742 | cell-cell adhesion via plasma-membrane adhesion molecules | 1.22e-07 |
| GO:0034728 | nucleosome organization | 1.24e-07 |
| GO:0061136 | regulation of proteasomal protein catabolic process | 1.30e-07 |
| GO:0045926 | negative regulation of growth | 1.65e-07 |
| GO:0019835 | cytolysis | 1.72e-07 |
| GO:0070160 | tight junction | 1.73e-07 |
| GO:0009826 | unidimensional cell growth | 1.73e-07 |
| GO:0046125 | pyrimidine deoxyribonucleoside metabolic process | 1.87e-07 |
| GO:0031349 | positive regulation of defense response | 1.88e-07 |
| GO:0016324 | apical plasma membrane | 2.43e-07 |
| GO:0046133 | pyrimidine ribonucleoside catabolic process | 2.93e-07 |
| GO:0034655 | nucleobase-containing compound catabolic process | 3.09e-07 |
| GO:0098609 | cell-cell adhesion | 3.09e-07 |
| GO:0010906 | regulation of glucose metabolic process | 3.21e-07 |
| GO:0002725 | negative regulation of T cell cytokine production | 3.35e-07 |
| GO:0005138 | interleukin-6 receptor binding | 3.35e-07 |
| GO:0072559 | NLRP3 inflammasome complex | 3.35e-07 |
| GO:2000551 | regulation of T-helper 2 cell cytokine production | 3.35e-07 |
| GO:0006900 | vesicle budding from membrane | 3.52e-07 |
| GO:1902554 | serine/threonine protein kinase complex | 3.65e-07 |
| GO:0031396 | regulation of protein ubiquitination | 3.67e-07 |
| GO:2001234 | negative regulation of apoptotic signaling pathway | 4.38e-07 |
| GO:0006903 | vesicle targeting | 4.48e-07 |
| GO:0001822 | kidney development | 4.60e-07 |
| GO:1903311 | regulation of mRNA metabolic process | 4.62e-07 |
| GO:0060326 | cell chemotaxis | 5.08e-07 |
| GO:0010761 | fibroblast migration | 5.41e-07 |
| GO:1903050 | regulation of proteolysis involved in cellular protein catabolic process | 5.63e-07 |
| GO:0048857 | neural nucleus development | 6.13e-07 |
| GO:0006839 | mitochondrial transport | 6.95e-07 |
| GO:0002323 | natural killer cell activation involved in immune response | 7.14e-07 |
| GO:0033139 | regulation of peptidyl-serine phosphorylation of STAT protein | 7.14e-07 |
| GO:2000662 | NA | 7.14e-07 |
| GO:0021799 | cerebral cortex radially oriented cell migration | 7.66e-07 |
| GO:0034599 | cellular response to oxidative stress | 9.15e-07 |
| GO:0031099 | regeneration | 9.77e-07 |
| GO:0016476 | regulation of embryonic cell shape | 1.10e-06 |
| GO:0035025 | positive regulation of Rho protein signal transduction | 1.10e-06 |
| GO:0007158 | neuron cell-cell adhesion | 1.35e-06 |
| GO:0008655 | pyrimidine-containing compound salvage | 1.37e-06 |
| GO:0043097 | pyrimidine nucleoside salvage | 1.37e-06 |
| GO:0008385 | IkappaB kinase complex | 1.39e-06 |
| GO:0045637 | regulation of myeloid cell differentiation | 1.56e-06 |
| GO:0005788 | endoplasmic reticulum lumen | 1.66e-06 |
| GO:0007213 | G protein-coupled acetylcholine receptor signaling pathway | 1.78e-06 |
| GO:0045471 | response to ethanol | 2.39e-06 |
| GO:2001181 | NA | 2.50e-06 |
| GO:0019218 | regulation of steroid metabolic process | 2.52e-06 |
| GO:0001703 | gastrulation with mouth forming first | 2.65e-06 |
| GO:0010004 | gastrulation involving germ band extension | 2.65e-06 |
| GO:0009120 | deoxyribonucleoside metabolic process | 2.65e-06 |
| GO:0060571 | morphogenesis of an epithelial fold | 3.12e-06 |
| GO:0046626 | regulation of insulin receptor signaling pathway | 3.20e-06 |
| GO:0005639 | integral component of nuclear inner membrane | 3.24e-06 |
| GO:0031229 | intrinsic component of nuclear inner membrane | 3.24e-06 |
| GO:0031056 | regulation of histone modification | 3.73e-06 |
| GO:1903320 | regulation of protein modification by small protein conjugation or removal | 4.10e-06 |
| GO:0002579 | positive regulation of antigen processing and presentation | 4.24e-06 |
| GO:0002710 | negative regulation of T cell mediated immunity | 4.24e-06 |
| GO:2000665 | NA | 4.24e-06 |
| GO:0044453 | NA | 4.72e-06 |
| GO:0043174 | nucleoside salvage | 4.82e-06 |
| GO:0035277 | spiracle morphogenesis, open tracheal system | 5.04e-06 |
| GO:0043014 | alpha-tubulin binding | 5.04e-06 |
| GO:0010675 | regulation of cellular carbohydrate metabolic process | 5.41e-06 |
| GO:1902115 | regulation of organelle assembly | 5.73e-06 |
| GO:1900076 | regulation of cellular response to insulin stimulus | 5.78e-06 |
| GO:1902911 | protein kinase complex | 6.10e-06 |
| GO:0095500 | acetylcholine receptor signaling pathway | 6.15e-06 |
| GO:1903831 | signal transduction involved in cellular response to ammonium ion | 6.15e-06 |
| GO:1905144 | response to acetylcholine | 6.15e-06 |
| GO:1905145 | cellular response to acetylcholine | 6.15e-06 |
| GO:0006006 | glucose metabolic process | 6.28e-06 |
| GO:0046135 | pyrimidine nucleoside catabolic process | 6.37e-06 |
| GO:0006954 | inflammatory response | 6.74e-06 |
| GO:0016887 | ATPase activity | 6.84e-06 |
| GO:0032611 | interleukin-1 beta production | 6.86e-06 |
| GO:0032612 | interleukin-1 production | 6.86e-06 |
| GO:2000484 | NA | 6.86e-06 |
| GO:0007088 | regulation of mitotic nuclear division | 6.91e-06 |
| GO:0005509 | calcium ion binding | 8.52e-06 |
| GO:0030473 | nuclear migration along microtubule | 9.36e-06 |
| GO:0032259 | methylation | 9.37e-06 |
| GO:0042623 | NA | 9.97e-06 |
| GO:0022617 | extracellular matrix disassembly | 1.04e-05 |
| GO:0044351 | macropinocytosis | 1.07e-05 |
| GO:0046597 | negative regulation of viral entry into host cell | 1.07e-05 |
| GO:0090197 | NA | 1.07e-05 |
| GO:1903829 | positive regulation of cellular protein localization | 1.15e-05 |
| GO:1903362 | regulation of cellular protein catabolic process | 1.20e-05 |
| GO:0010008 | endosome membrane | 1.23e-05 |
| GO:0070059 | intrinsic apoptotic signaling pathway in response to endoplasmic reticulum stress | 1.28e-05 |
| GO:2001179 | NA | 1.60e-05 |
| GO:0043296 | apical junction complex | 1.65e-05 |
| GO:0035235 | ionotropic glutamate receptor signaling pathway | 1.76e-05 |
| GO:0045121 | membrane raft | 1.78e-05 |
| GO:0098857 | membrane microdomain | 1.78e-05 |
| GO:0018107 | peptidyl-threonine phosphorylation | 1.84e-05 |
| GO:0009225 | nucleotide-sugar metabolic process | 1.91e-05 |
| GO:0090435 | protein localization to nuclear envelope | 2.29e-05 |
| GO:0090196 | NA | 2.33e-05 |
| GO:0051090 | regulation of DNA-binding transcription factor activity | 2.35e-05 |
| GO:0005525 | GTP binding | 2.60e-05 |
| GO:0050900 | leukocyte migration | 2.94e-05 |
| GO:0006323 | DNA packaging | 2.95e-05 |
| GO:0002577 | regulation of antigen processing and presentation | 3.30e-05 |
| GO:0032674 | regulation of interleukin-5 production | 3.30e-05 |
| GO:0070567 | cytidylyltransferase activity | 3.30e-05 |
| GO:0070700 | BMP receptor binding | 3.30e-05 |
| GO:2000482 | NA | 3.30e-05 |
| GO:0019673 | GDP-mannose metabolic process | 3.34e-05 |
| GO:0042454 | ribonucleoside catabolic process | 3.38e-05 |
| GO:0045862 | positive regulation of proteolysis | 3.45e-05 |
| GO:0030307 | positive regulation of cell growth | 3.62e-05 |
| GO:0098589 | membrane region | 3.66e-05 |
| GO:0018210 | peptidyl-threonine modification | 3.79e-05 |
| GO:0021795 | cerebral cortex cell migration | 3.83e-05 |
| GO:0046134 | pyrimidine nucleoside biosynthetic process | 4.16e-05 |
| GO:0090092 | regulation of transmembrane receptor protein serine/threonine kinase signaling pathway | 4.21e-05 |
| GO:0097153 | cysteine-type endopeptidase activity involved in apoptotic process | 4.58e-05 |
| GO:0050679 | positive regulation of epithelial cell proliferation | 4.61e-05 |
| GO:0030517 | negative regulation of axon extension | 4.62e-05 |
| GO:0002707 | negative regulation of lymphocyte mediated immunity | 5.07e-05 |
| GO:0045732 | positive regulation of protein catabolic process | 5.24e-05 |
| GO:0050688 | regulation of defense response to virus | 6.14e-05 |
| GO:0002829 | negative regulation of type 2 immune response | 6.22e-05 |
| GO:0032656 | regulation of interleukin-13 production | 6.22e-05 |
| GO:0009612 | response to mechanical stimulus | 6.64e-05 |
| GO:0032550 | purine ribonucleoside binding | 6.83e-05 |
| GO:0046131 | pyrimidine ribonucleoside metabolic process | 7.40e-05 |
| GO:0072593 | reactive oxygen species metabolic process | 7.57e-05 |
| GO:0032549 | ribonucleoside binding | 7.66e-05 |
| GO:2001252 | positive regulation of chromosome organization | 7.66e-05 |
| GO:0072321 | chaperone-mediated protein transport | 7.97e-05 |
| GO:0006888 | endoplasmic reticulum to Golgi vesicle-mediated transport | 7.98e-05 |
| GO:0090287 | regulation of cellular response to growth factor stimulus | 8.06e-05 |
| GO:0090314 | positive regulation of protein targeting to membrane | 8.31e-05 |
| GO:0005912 | adherens junction | 8.65e-05 |
| GO:0001883 | purine nucleoside binding | 9.06e-05 |
| GO:0030864 | cortical actin cytoskeleton | 9.49e-05 |
| GO:0002709 | regulation of T cell mediated immunity | 9.67e-05 |
| GO:0005521 | lamin binding | 9.77e-05 |
| GO:0043414 | macromolecule methylation | 0.000101236 |
| GO:0036126 | sperm flagellum | 0.000101508 |
| GO:0071824 | protein-DNA complex subunit organization | 0.000106939 |
| GO:0044440 | NA | 0.000107279 |
| GO:0042104 | positive regulation of activated T cell proliferation | 0.000108957 |
| GO:0097202 | activation of cysteine-type endopeptidase activity | 0.000108957 |
| GO:0016323 | basolateral plasma membrane | 0.000112709 |
| GO:0016857 | racemase and epimerase activity, acting on carbohydrates and derivatives | 0.000116065 |
| GO:0072529 | pyrimidine-containing compound catabolic process | 0.000125234 |
| GO:0046875 | ephrin receptor binding | 0.000129484 |
| GO:0006109 | regulation of carbohydrate metabolic process | 0.000133787 |
| GO:0035011 | melanotic encapsulation of foreign target | 0.000135518 |
| GO:0032561 | guanyl ribonucleotide binding | 0.000139969 |
| GO:0005523 | tropomyosin binding | 0.000141005 |
| GO:0032688 | negative regulation of interferon-beta production | 0.000141005 |
| GO:0032355 | response to estradiol | 0.000141775 |
| GO:0031625 | ubiquitin protein ligase binding | 0.000144053 |
| GO:0051607 | defense response to virus | 0.000144862 |
| GO:0048487 | beta-tubulin binding | 0.000149061 |
| GO:0019001 | guanyl nucleotide binding | 0.000163874 |
| GO:0005798 | Golgi-associated vesicle | 0.000164753 |
| GO:0051289 | protein homotetramerization | 0.000171166 |
| GO:0006206 | pyrimidine nucleobase metabolic process | 0.000173384 |
| GO:0019318 | hexose metabolic process | 0.000175744 |
| GO:0070161 | anchoring junction | 0.000176504 |
| GO:0001882 | nucleoside binding | 0.000181757 |
| GO:0097729 | 9+2 motile cilium | 0.000210283 |
| GO:0090313 | regulation of protein targeting to membrane | 0.000223166 |
| GO:0002719 | negative regulation of cytokine production involved in immune response | 0.000227236 |
| GO:0002724 | regulation of T cell cytokine production | 0.000227236 |
| GO:0070696 | transmembrane receptor protein serine/threonine kinase binding | 0.000227236 |
| GO:0009164 | nucleoside catabolic process | 0.000235803 |
| GO:0044389 | ubiquitin-like protein ligase binding | 0.000249206 |
| GO:1902275 | regulation of chromatin organization | 0.000258704 |
| GO:0002347 | response to tumor cell | 0.000264105 |
| GO:0008656 | cysteine-type endopeptidase activator activity involved in apoptotic process | 0.000283589 |
| GO:0046596 | regulation of viral entry into host cell | 0.000283589 |
| GO:0007623 | circadian rhythm | 0.000285653 |
| GO:0061001 | regulation of dendritic spine morphogenesis | 0.000287835 |
| GO:0022029 | telencephalon cell migration | 0.000299177 |
| GO:0016854 | racemase and epimerase activity | 0.000303479 |
| GO:0003382 | epithelial cell morphogenesis | 0.000304161 |
| GO:0017048 | Rho GTPase binding | 0.000315985 |
| GO:0051879 | Hsp90 protein binding | 0.000325562 |
| GO:0021549 | cerebellum development | 0.000387972 |
| GO:0021885 | forebrain cell migration | 0.000398935 |
| GO:0006547 | histidine metabolic process | 0.000403556 |
| GO:0006548 | histidine catabolic process | 0.000403556 |
| GO:0052803 | imidazole-containing compound metabolic process | 0.000403556 |
| GO:0052805 | imidazole-containing compound catabolic process | 0.000403556 |
| GO:0007215 | glutamate receptor signaling pathway | 0.000413313 |
| GO:0002828 | regulation of type 2 immune response | 0.000428805 |
| GO:0042100 | B cell proliferation | 0.000428805 |
| GO:0002819 | regulation of adaptive immune response | 0.000442907 |
| GO:0046890 | regulation of lipid biosynthetic process | 0.000458918 |
| GO:1903901 | negative regulation of viral life cycle | 0.000463989 |
| GO:0035010 | encapsulation of foreign target | 0.000465316 |
| GO:0002704 | negative regulation of leukocyte mediated immunity | 0.000475131 |
| GO:0050707 | regulation of cytokine secretion | 0.000506224 |
| GO:0035006 | melanization defense response | 0.000514552 |
| GO:0006907 | pinocytosis | 0.000520314 |
| GO:0000079 | regulation of cyclin-dependent protein serine/threonine kinase activity | 0.000574084 |
| GO:0006486 | protein glycosylation | 0.000577868 |
| GO:0043413 | macromolecule glycosylation | 0.000577868 |
| GO:0072594 | establishment of protein localization to organelle | 0.000608252 |
| GO:0006213 | pyrimidine nucleoside metabolic process | 0.000613936 |
| GO:0043028 | cysteine-type endopeptidase regulator activity involved in apoptotic process | 0.000613936 |
| GO:0043094 | cellular metabolic compound salvage | 0.000613936 |
| GO:1901658 | glycosyl compound catabolic process | 0.000613936 |
| GO:0001773 | myeloid dendritic cell activation | 0.000626313 |
| GO:2000778 | NA | 0.000626313 |
| GO:0071103 | DNA conformation change | 0.000657502 |
| GO:0032729 | positive regulation of interferon-gamma production | 0.000694678 |
| GO:0035255 | ionotropic glutamate receptor binding | 0.000720386 |
| GO:0050716 | NA | 0.00074831 |
| GO:0050718 | NA | 0.00074831 |
| GO:0090200 | positive regulation of release of cytochrome c from mitochondria | 0.00074831 |
| GO:0007266 | Rho protein signal transduction | 0.000817809 |
| GO:0045179 | apical cortex | 0.000817809 |
| GO:0016763 | transferase activity, transferring pentosyl groups | 0.000881793 |
| GO:0016505 | peptidase activator activity involved in apoptotic process | 0.000887881 |
| GO:0032733 | positive regulation of interleukin-10 production | 0.000887881 |
| GO:0022037 | metencephalon development | 0.000909333 |
| GO:0046425 | regulation of receptor signaling pathway via JAK-STAT | 0.000909333 |
| GO:1904029 | regulation of cyclin-dependent protein kinase activity | 0.000953742 |
| GO:1904892 | regulation of receptor signaling pathway via STAT | 0.000962628 |
| GO:0002823 | negative regulation of adaptive immune response based on somatic recombination of immune receptors built from immunoglobulin superfamily domains | 0.001046664 |
| GO:0048511 | rhythmic process | 0.001058401 |
| GO:0060998 | regulation of dendritic spine development | 0.001119029 |
| GO:1903332 | regulation of protein folding | 0.001125665 |
| GO:1905936 | regulation of germ cell proliferation | 0.001125665 |
| GO:0051015 | actin filament binding | 0.001138695 |
| GO:0002820 | negative regulation of adaptive immune response | 0.001226361 |
| GO:0030101 | natural killer cell activation | 0.001226361 |
| GO:0046006 | regulation of activated T cell proliferation | 0.001226361 |
| GO:0042176 | regulation of protein catabolic process | 0.001244611 |
| GO:0030295 | protein kinase activator activity | 0.001270545 |
| GO:0070085 | glycosylation | 0.00132466 |
| GO:0035023 | regulation of Rho protein signal transduction | 0.001363103 |
| GO:0072528 | pyrimidine-containing compound biosynthetic process | 0.001376625 |
| GO:0005996 | monosaccharide metabolic process | 0.001390688 |
| GO:0002230 | positive regulation of defense response to virus by host | 0.001428728 |
| GO:0015171 | amino acid transmembrane transporter activity | 0.001520673 |
| GO:0006582 | melanin metabolic process | 0.00152347 |
| GO:0019209 | kinase activator activity | 0.001564973 |
| GO:0033612 | receptor serine/threonine kinase binding | 0.001655576 |
| GO:0060337 | type I interferon signaling pathway | 0.001655576 |
| GO:0071357 | cellular response to type I interferon | 0.001655576 |
| GO:0050771 | negative regulation of axonogenesis | 0.001790695 |
| GO:0030863 | cortical cytoskeleton | 0.001790856 |
| GO:1900244 | positive regulation of synaptic vesicle endocytosis | 0.001848337 |
| GO:0034656 | nucleobase-containing small molecule catabolic process | 0.001875501 |
| GO:0002701 | negative regulation of production of molecular mediator of immune response | 0.001908761 |
| GO:2000406 | positive regulation of T cell migration | 0.001908761 |
| GO:0031532 | actin cytoskeleton reorganization | 0.002042852 |
| GO:0009112 | nucleobase metabolic process | 0.002070192 |
| GO:0050706 | NA | 0.002190184 |
| GO:0030135 | coated vesicle | 0.002366863 |
| GO:0016328 | lateral plasma membrane | 0.002634106 |
| GO:0006281 | DNA repair | 0.002641981 |
| GO:0006220 | pyrimidine nucleotide metabolic process | 0.002644703 |
| GO:0003333 | amino acid transmembrane transport | 0.002678366 |
| GO:0008347 | glial cell migration | 0.002678366 |
| GO:0016049 | cell growth | 0.0027565 |
| GO:0032496 | response to lipopolysaccharide | 0.002772094 |
| GO:0032731 | positive regulation of interleukin-1 beta production | 0.002845526 |
| GO:0034340 | response to type I interferon | 0.002845526 |
| GO:0006998 | nuclear envelope organization | 0.002873387 |
| GO:0005759 | mitochondrial matrix | 0.002980457 |
| GO:0051193 | NA | 0.003038509 |
| GO:0043330 | response to exogenous dsRNA | 0.003223416 |
| GO:0050704 | NA | 0.003223416 |
| GO:1990204 | oxidoreductase complex | 0.003263096 |
| GO:0018205 | peptidyl-lysine modification | 0.003527859 |
| GO:0017080 | sodium channel regulator activity | 0.00356877 |
| GO:1903423 | positive regulation of synaptic vesicle recycling | 0.00356877 |
| GO:0005637 | nuclear inner membrane | 0.003608656 |
| GO:0032653 | regulation of interleukin-10 production | 0.003637472 |
| GO:0051085 | chaperone cofactor-dependent protein refolding | 0.00379288 |
| GO:1903900 | regulation of viral life cycle | 0.004233768 |
| GO:0031685 | adenosine receptor binding | 0.004265014 |
| GO:0050542 | icosanoid binding | 0.004265014 |
| GO:0002237 | response to molecule of bacterial origin | 0.004373126 |
| GO:0031638 | zymogen activation | 0.004582252 |
| GO:0032732 | positive regulation of interleukin-1 production | 0.004582252 |
| GO:0010755 | regulation of plasminogen activation | 0.004611554 |
| GO:0048525 | negative regulation of viral process | 0.004891926 |
| GO:0032153 | cell division site | 0.00500421 |
| GO:0032155 | NA | 0.00500421 |
| GO:2000404 | regulation of T cell migration | 0.005117087 |
| GO:0021766 | hippocampus development | 0.005333679 |
| GO:0031514 | motile cilium | 0.005670754 |
| GO:0016191 | synaptic vesicle uncoating | 0.005713061 |
| GO:0072318 | clathrin coat disassembly | 0.005713061 |
| GO:1903961 | positive regulation of anion transmembrane transport | 0.005713061 |
| GO:0002706 | regulation of lymphocyte mediated immunity | 0.00598618 |
| GO:0034124 | regulation of MyD88-dependent toll-like receptor signaling pathway | 0.006587962 |
| GO:0099175 | regulation of postsynapse organization | 0.006906682 |
| GO:0033227 | dsRNA transport | 0.00704443 |
| GO:0072319 | vesicle uncoating | 0.007421205 |
| GO:0035254 | glutamate receptor binding | 0.007870199 |
| GO:0071158 | positive regulation of cell cycle arrest | 0.007870199 |
| GO:0007413 | axonal fasciculation | 0.008388755 |
| GO:0071242 | cellular response to ammonium ion | 0.008388755 |
| GO:0106030 | neuron projection fasciculation | 0.008388755 |
| GO:0032649 | regulation of interferon-gamma production | 0.008492965 |
| GO:0032722 | positive regulation of chemokine production | 0.008497885 |
| GO:0042531 | positive regulation of tyrosine phosphorylation of STAT protein | 0.008497885 |
| GO:2000403 | positive regulation of lymphocyte migration | 0.008497885 |
| GO:0009100 | glycoprotein metabolic process | 0.008910226 |
| GO:1900242 | regulation of synaptic vesicle endocytosis | 0.008933721 |
| GO:0045581 | negative regulation of T cell differentiation | 0.009329592 |
| GO:0051918 | negative regulation of fibrinolysis | 0.009399524 |
| GO:0061635 | regulation of protein complex stability | 0.009399524 |
| GO:0002821 | positive regulation of adaptive immune response | 0.010100785 |
| GO:0048013 | ephrin receptor signaling pathway | 0.010726141 |
| GO:0050678 | regulation of epithelial cell proliferation | 0.010767668 |
| GO:0051262 | protein tetramerization | 0.010909672 |
| GO:0010955 | negative regulation of protein processing | 0.011164433 |
| GO:0032480 | negative regulation of type I interferon production | 0.011164433 |
| GO:1903318 | negative regulation of protein maturation | 0.011164433 |
| GO:0043525 | positive regulation of neuron apoptotic process | 0.011380116 |
| GO:0044829 | positive regulation by host of viral genome replication | 0.011656031 |
| GO:0099738 | cell cortex region | 0.011713603 |
| GO:0090199 | regulation of release of cytochrome c from mitochondria | 0.012171401 |
| GO:0007349 | cellularization | 0.012820267 |
| GO:0002286 | T cell activation involved in immune response | 0.013240561 |
| GO:0043124 | negative regulation of I-kappaB kinase/NF-kappaB signaling | 0.013240561 |
| GO:0070252 | actin-mediated cell contraction | 0.013402763 |
| GO:0006458 | 'de novo' protein folding | 0.013705678 |
| GO:0045178 | basal part of cell | 0.014005257 |
| GO:0005890 | sodium:potassium-exchanging ATPase complex | 0.01419684 |
| GO:0061200 | clathrin-sculpted gamma-aminobutyric acid transport vesicle | 0.01419684 |
| GO:0061202 | clathrin-sculpted gamma-aminobutyric acid transport vesicle membrane | 0.01419684 |
| GO:0050715 | positive regulation of cytokine secretion | 0.014297473 |
| GO:0032648 | regulation of interferon-beta production | 0.015572664 |
| GO:0050710 | negative regulation of cytokine secretion | 0.015572664 |
| GO:0042135 | neurotransmitter catabolic process | 0.015634814 |
| GO:0051084 | 'de novo' posttranslational protein folding | 0.015634814 |
| GO:0050770 | regulation of axonogenesis | 0.015750778 |

**Supplementary Table S10. Comparison of the quality of the two genomes.**

| **Term** | **This study** | **NCBI (VGP version)** |
| --- | --- | --- |
| Genome size (Mb) | 834 | 589 |
| Number of chromosomes | 26 | 26 |
| Contig N50 (Mb) | 21.44 | 7.1 |
| Scaffold N50 (Mb) | 30.38 | 25.66 |
| Number of telomeres | 46 | 35 |
| Number of gap-free chromosomes | 17 | 0 |
| Number of gaps | 18 | 223 |
| BUSCO score (%) | 97.30 | 97.10 |

Note: VGP (Vertebrate Genomes Project) version is from NCBI (GCA_013358815.1).

**
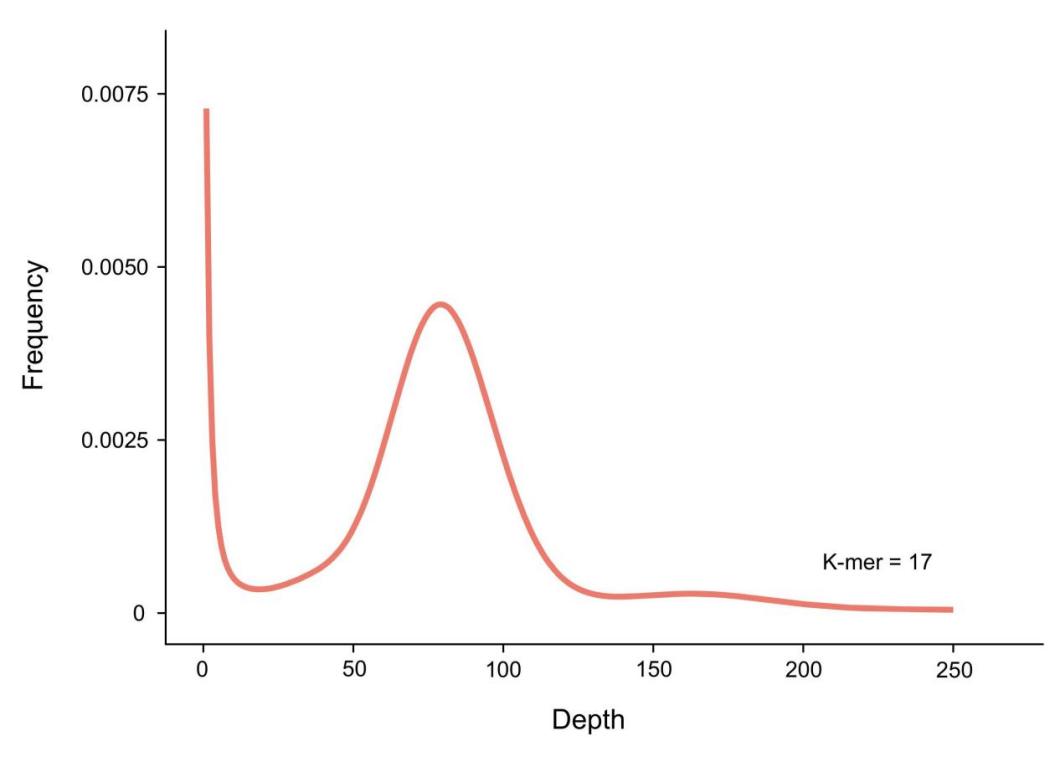
**

**Supplementary Fig. S1. Genomic characteristics of *Electrophorus electricus*.** The X-axis shows thek-mer depth, and the Y-axis shows the frequency of the k-mer for a given depth. The evaluated genome size of *E. electricus* is approximately 789.61 Mb.

**
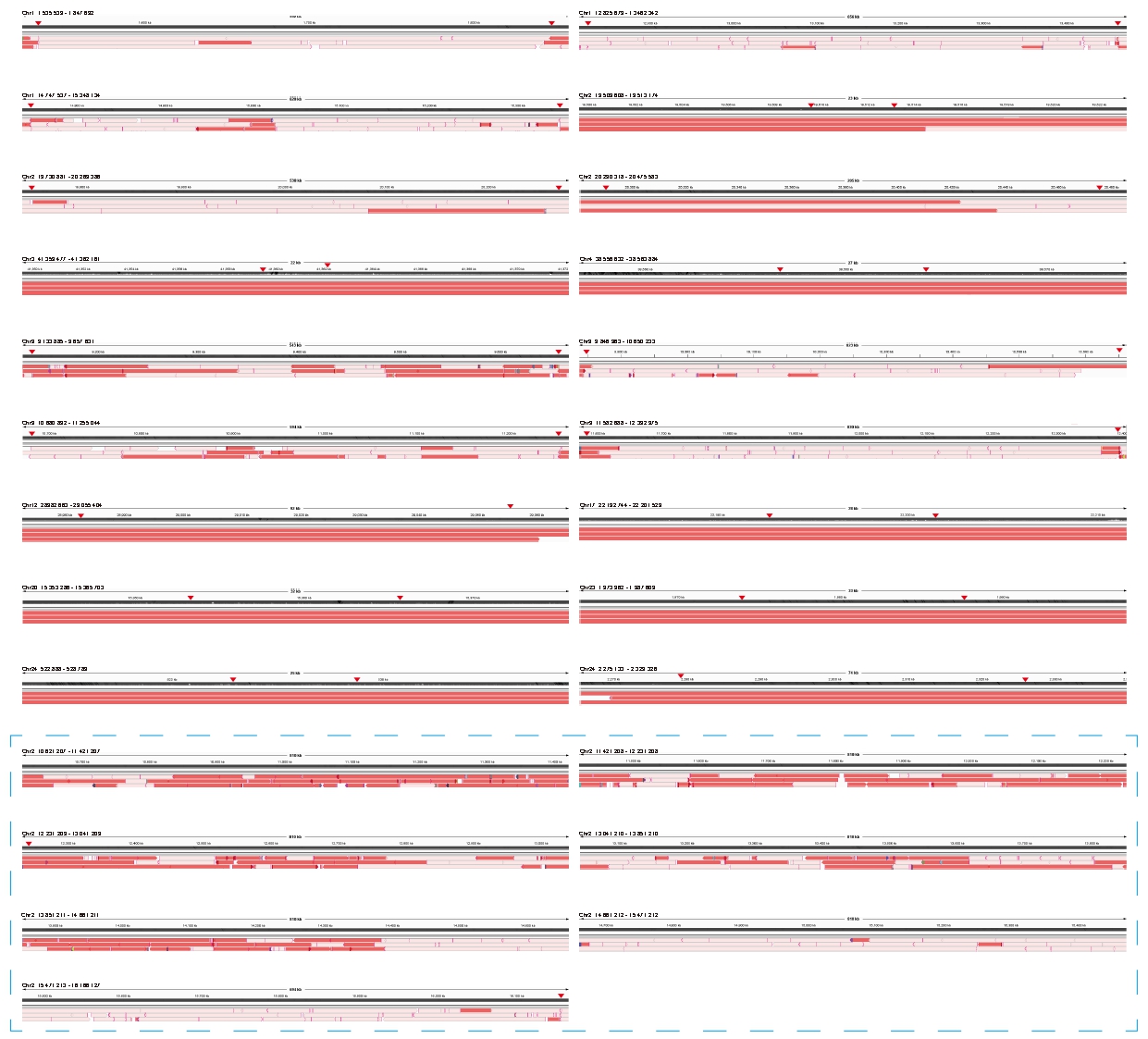
**

**Supplementary Fig. S2. Sequence mapping during genome gap filling.** IGV software was used to display the 10 Kb upstream and downstream regions of the gap in the ONT alignment data. The red triangles represent the position information of the gap.

**
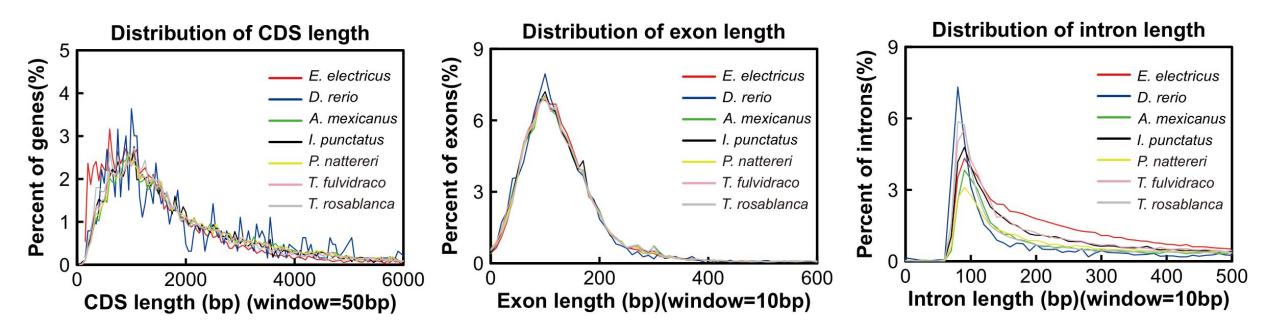
**

**Supplementary Fig. S3. Comparative analysis of CDS, exon, and intron length distributions across species.**


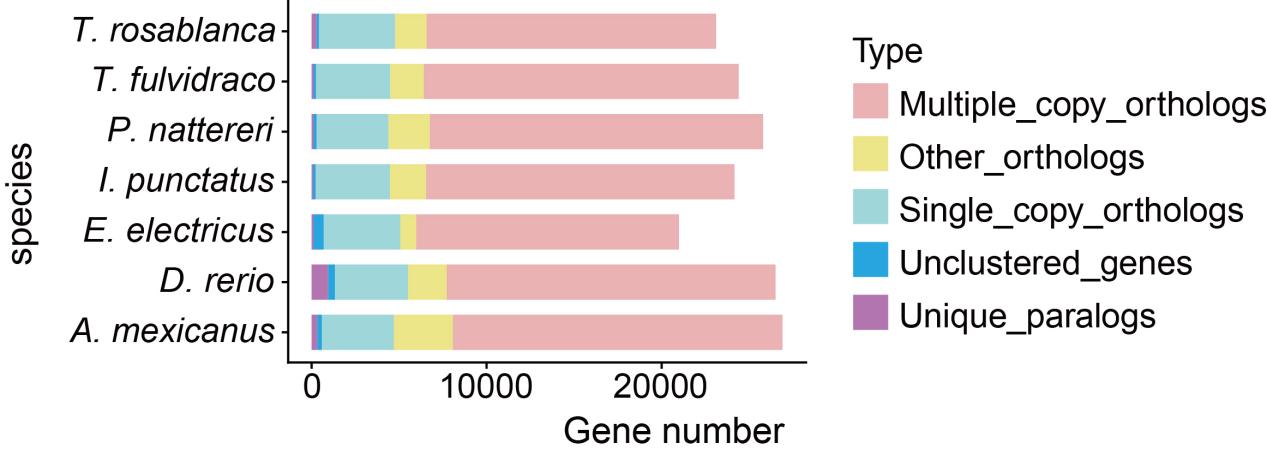


**Supplementary Fig. S4. Identification of orthologous genes among the species.**


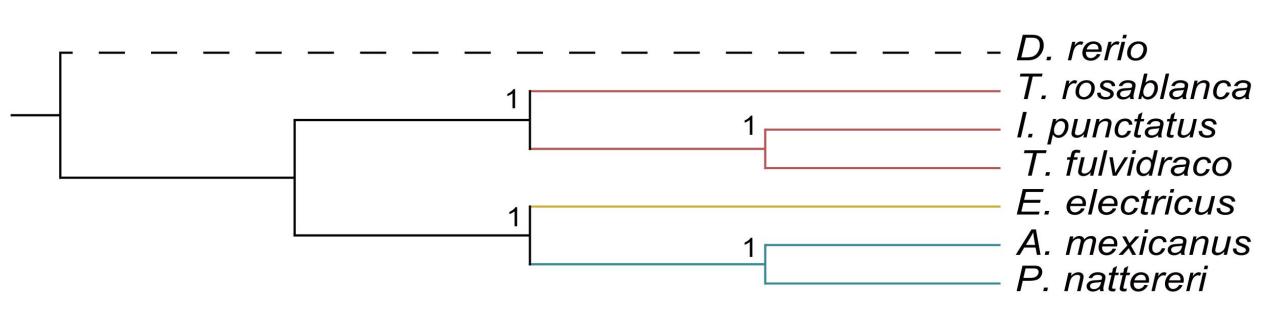


**Supplementary Fig. S5. Species tree construction with ASTRAL using RAxML-derived gene trees.** The number at each node indicates the local posterior probability.

**
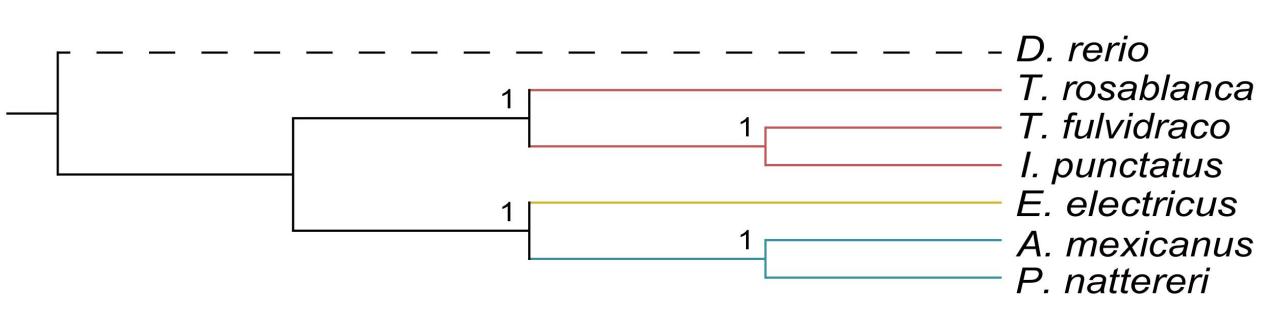
**

**Supplementary Fig. S6. Species tree construction with ASTRAL using IQ-TREE-derived gene trees.** The number at each node indicates the local posterior probability.


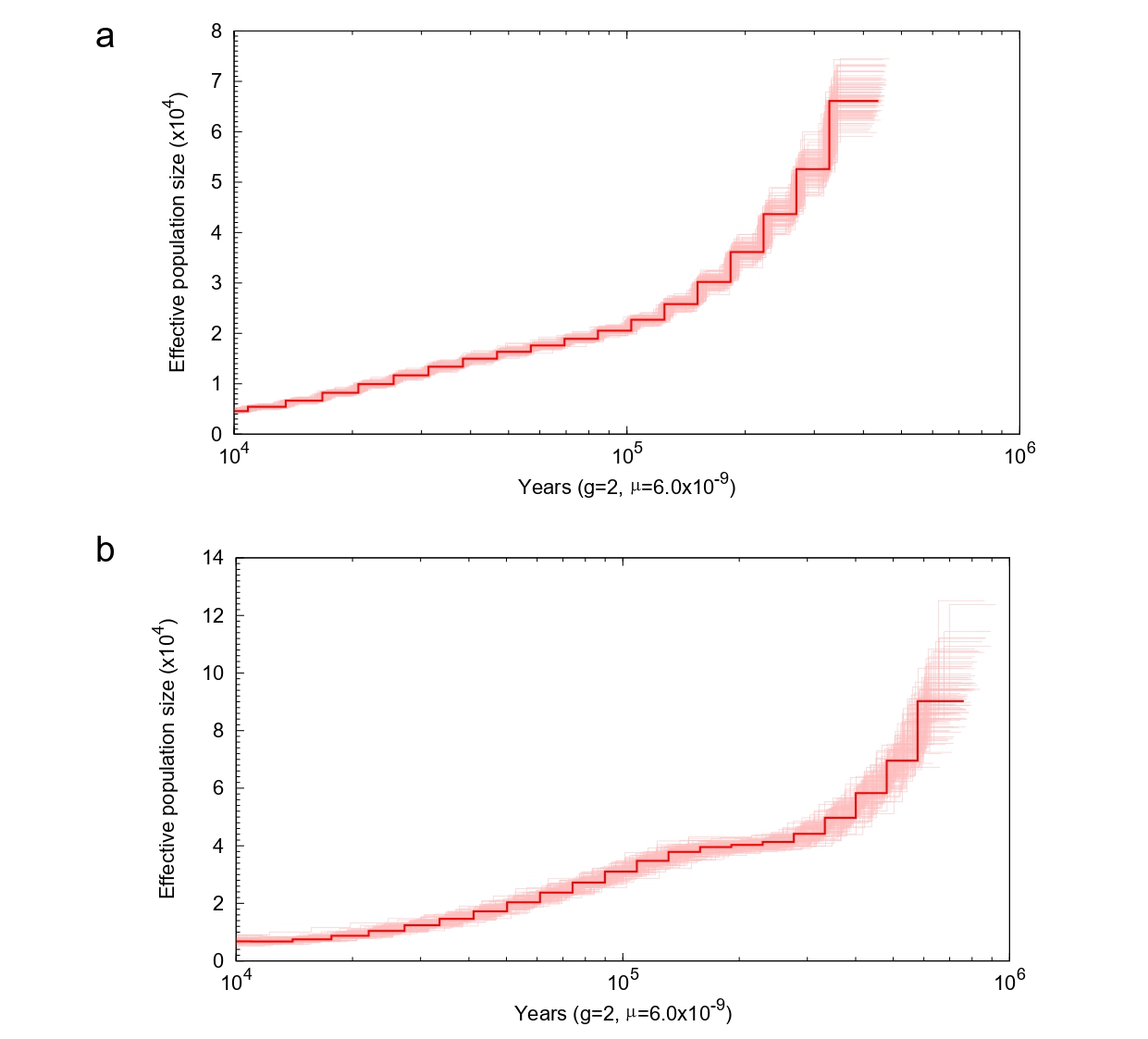


**Supplementary Fig. S7. Population history was analyzed using two additional individuals of *E. electricus*.** The x-axis represents past years, and the y-axis represents the effective population size of the species; “g” is the generation time, and “μ” is the mutation rate of species. **a.** The population history was calculated by mapping the sequencing data (SRR1302052) to its corresponding genome assembly. **b.** The population history was calculated by mapping the sequencing data (SRR10752306; SRR10752307) o its corresponding genome assembly.
