# Supplementary material for: Telomere-to-telomere genome assembly of Electrophorus electricus provides insights into the evolution of electric eels
Source: Gigascience. 2025 Apr 1;14:giaf024. doi: 10.1093/gigascience/giaf024 (PMC11959694; doi:10.1093/gigascience/giaf024)
Supplement: giaf024_GIGA-D-24-00300_Revision_1 [file giaf024_giga-d-24-00300_revision_1.pdf]

GigaScience

Telomere-to-telomere genome assembly of Electrophorus electricus provides insights into the evolution of electric eels

--Manuscript Draft--

Manuscript Number:	GIGA-D-24-00300R1
Full Title:	Telomere-to-telomere genome assembly of Electrophorus electricus provides insights into the evolution of electric eels
Article Type:	Data Note
Funding Information:	National Natural Science Foundation of China (31900312) — Prof. Yongxin Li
Abstract:	Background: Electric eels evolved remarkable electric organs that enable them to instantaneously discharge hundreds of volts for predation, defense, and communication. However, the absence of a high-quality reference genome has extremely constrained the studies of electric eels in various aspects.<br>Results: Using high-depth, multi-platform sequencing data, we successfully assembled the first telomere-to-telomere high-quality reference genome of Electrophorus electricus, which has a genome size of 833.43 Mb and comprises 26 chromosomes. Multiple evaluations, including N50 statistics (30.38 Mb), BUSCO scores (97.30%), and mapping ratio of short-insert sequencing data (99.91%) demonstrate the high contiguity and completeness of the electric eel genome assembly we obtained. Genome annotation predicted 396.63 Mb repetitive sequences and 20,992 protein-coding genes. Furthermore, evolutionary analyses indicate that Gymnotiformes, which the electric eel belongs to, has a closer relationship with Characiformes than Siluriformes, and diverged from Characiformes at 103.20 million years ago. Pairwise sequentially Markovian coalescent analysis found that the electric eel had a stable population size during the Chibanian stage in Pleistocene, but its population dramatically decreased from the subsequent period still now. Furthermore, many regulatory factors related to neurotransmitters and classical signaling pathways during embryonic development were significantly expanded, which may provide insights into the potential genetic underpinnings of the exceptional ability of electric eels to discharge high-voltage electricity.<br>Conclusions: This study not only provided the first high-quality telomere-to-telomere reference genome of the electric eel, but also offers insights into the potential genetic mechanisms underlying the exceptional ability of electric eels to discharge high-voltage electricity.
Corresponding Author:	Yongxin Li, Ph.D.<br><br>CHINA
Corresponding Author Secondary Information:	
Corresponding Author's Institution:	
Corresponding Author's Secondary Institution:	
First Author:	Zan Qi
First Author Secondary Information:	
Order of Authors:	Zan Qi<br>Qun Liu<br>Haorong Li<br>Yaolei Zhang<br>Ziwei Yu<br>Wenkai Luo

Powered by Editorial Manager® and ProduXion Manager® from Aries Systems Corporation

	Kun Wang
	Yuxin Zhang
	Shoupeng Pan
	Chao Wang
	Hui Jiang
	Qiang Qiu
	Wen Wang
	Guangyi Fan
	Yongxin Li, Ph.D.
**Order of Authors Secondary Information:**	
**Response to Reviewers:**	Reviewer #1:<br>1. Qi et al. described a high-quality of E. electricus with many chromosomes reaching the T2T level. While this genome assembly can serve as an interesting resource for a fish T2T genome, very few novel insights have been revealed by the analysis.<br>Response: Thank you for recognizing the quality of our genome. The main goal of this work is to provide a higher-quality reference genome for the electric eel within the field, so that other scientists in the field can join us in our efforts to study the key scientific questions of electric eel. Therefore, we submitted this manuscript as a Data Note to this journal. For several key scientific questions, we have conducted systematic research using multiple sequencing data and experiment validation in 2-3 other works that we are currently preparing. Thank you for pointing out this issue.<br><br>2. There are at least three additional E. electricus genome assemblies available in NCBI. I am surprised that the authors mentioned none of them, nor comparing them with their assembly. Note the VGP E. electricus also has a high-quality with two haploid genomes assembled. Curiously, the genome sizes vary substantially among the assemblies: the VGP assembly has a ~600 Mb genome size, but the assembly from this study is larger than 800 Mb. Why?<br>Response: Thank you for your valuable suggestion. In this revision, we have added a description of the electric eel VGP genome in the Introduction and incorporated results comparing the quality of the electric eel VGP genome here in the Results section. Regarding the genome size estimation, we used two methods, which have been utilized in numerous high-quality publications, to estimate it, all yielding results close to 800 Mb (see Supplementary Table S2 and Supplementary Fig. S1), indicating that the electric eel's genome size is indeed approximately 800 Mb. This also corresponds well with our actual genome assembly results. Thank you once again for your invaluable suggestion.<br><br>3. There are also various RNA-seq datasets available, but the authors did not try to analysis the RNA-seq data. This manuscript provides only genomic description, thus very limited power in addressing the questions they brought up in the Introduction.<br>Response: Thank you for your suggestion. As we mentioned above, the main goal of this work is to provide a higher-quality reference genome for the electric eel within the field, so that other scientists in the field can join us in our efforts to study the key scientific questions of electric eel. Therefore, we submitted this manuscript as a Data Note to this journal. For several key scientific questions, we have conducted systematic research using many data (including the RNA-seq data) in 2-3 other works that we are currently preparing. Once again, thank you for your valuable suggestion.<br><br>4. TGS-gapcloser is known to cause false-positives in closing the gaps. The authors need to make sure they have manually inspected all gaps that were filled with TGS-gapcloser.<br>Response: TGS-GapCloser is currently a widely used software for filling gaps, as evidenced in studies on the Yangtze finless porpoise and East Asian finless porpoise (10.1093/gigascience/giae067), Hawaiian crickets (10.1038/s41467-024-49344-4), and snakes (10.1126/science.adj7026). As you mentioned, it may occasionally produce false positives when closing gaps. Therefore, we carefully re-examined these filled gaps using ONT ultra-long sequencing data. The results showed that all gaps filled by

TGS-GapCloser were supported by ONT sequences, indicating that the gaps we filled using the software were indeed correct. Thank you for your suggestion.

Minor comments:
5. L45, it's not fully accurate to describe the fish as the oldest vertebrate group.
Response: Thank you for pointing out this issue. We changed the description with "As one of the oldest vertebrate groups".

6. L77, there are also fish T2T genomes published, including the zig-zag eel.
Response: In this revision, we have included the zig-zag eel. Thank you for pointing out this issue.

7. L128, hifiasm produces two haploid genomes. Did the author randemly pick one, or simply use the primary contigs?
Response: Yes, we used the primary contigs. Thank you.

8. L131, I do not suggest to do pilon polishing for HiFi assembly.
Response: As you mentioned, generally, it is not necessary to perform the polishing step when using only HiFi reads for contig-level assembly. However, to obtain a high-contiguity genome assembly, we combined PacBio HiFi reads and ONT ultra-long reads for contig assembly. As we all know, ultra-long reads typically have a relatively higher single-base error rate compared to HiFi reads. Therefore, we conducted Pilon polishing after the contig-level assembly, a practice that has been adopted in several high-quality publications. Thank you for your suggestion.

9. L236, in general, many analyses involve custom Perl scripts. All those scripts need to be uploaded to a public repository.
Response: As you suggested, we have uploaded the key Perl scripts that we wrote to the GigaDB in this revision. For other scripts that embedded in public software, we have provided detailed descriptions of the key parameters in the Methods section. Thank you for your suggestion.

10. L294, "the lack of a high-quality reference genome…" this is not true. At the VGP assembly is of high quality.
Response: We have changed the description with "Therefore, a genome of the electric eel with higher contiguity and completeness is urgently needed." Thank you.

11. L298, the k-mer distribution plot looks very strange, without an obvious homozygous peak. I suggest to use a larger k-mer.
Response: Sorry for our inaccurate description. In fact, the k-mer distribution curve appears quite normal, featuring a very prominent main peak (homozygous peak) at a kmer depth of 83. We have corrected the writing in this revision. Thank you for bringing this issue to our attention.

12. L311, continuity should be contiguity? I do not see the main Tables.
Response: Thanks for your suggestion. In this revision, we changed the word "continuity" with "contiguity". Regarding the main Tables, we have re-examined them in the merged PDF downloaded from the GigaScience website in this revision, and confirmed that all the main Tables are displayed normally in the manuscript. Regarding the Supplementary file (including supplementary tables and figures, especially the Supplementary Table S3), you could download it directly at the last page of the merged PDF file.

13. L346, having assembled the telomeres doesn't mean the genome assembly is telomere-to-telomere. In fact, there are still a few gaps in some chromosomes. The authors could at best describe some of the chromosomes T2T.
Response: Yes, we agree with the issues you raised. Therefore, in this revision, we revised this sentence to "Finally, we identified 46 telomeres and 26 centromeres in the chromosome-level genome, indicating that we have successfully obtained a high-quality reference genome for E. electricus, with many chromosomes achieving the telomere-to-telomere assembly". Thanks for your suggestion.

14. The Discussion part is mostly a summary of the results, without providing useful insights or interpretations.

	Response: Yes, we fully agree with the issues you raised. Therefore, in this revision, we have systematically and carefully revised and re-organized the Discussion section of the paper. Thank you for your valuable suggestion.<br><br>Reviewer #2:<br>Minor:<br>1."An individual electric eel (E. electricus) used in this study was procured from the aquatic pet market." - this is relevant. Is there a way to define the origin of the individual sampled?<br>Response: Based on existing records, the primary habitat of the electric eel is in the Amazon Basin of South America. However, the sample mentioned in this paper was obtained from the aquarium pet market in China. Therefore, in this revision, we have clearly stated in the Introduction section that its main distribution area is the Amazon River Basin, and specified in the Sampling section of the Methods that the sample was acquired from the aquarium pet market in China. Thank you for your valuable suggestion.<br><br>2. "Our analysis identified 425 gene families that have undergone significant expansion" - it would be interesting to detail some genes specifically with phylogenetic analysis to validate that these expansions are artifacts.<br>Response: Thanks for your suggestion. We constructed the phylogenetic tree based on single copy homologous genes, and then inferred the divergence time among species based on the fossil data from TimeTree database. Based on the fossil-calibrated phylogenetic tree, we identified 425 expanded gene families in the electric eel genome. Subsequently, we extracted several genes for validation and confirmed to have indeed undergone expansion. Thanks again for your valuable suggestion.
**Additional Information:**	
**Question**	**Response**
Are you submitting this manuscript to a special series or article collection?	No
**Experimental design and statistics**<br><br>Full details of the experimental design and statistical methods used should be given in the Methods section, as detailed in our Minimum Standards Reporting Checklist. Information essential to interpreting the data presented should be made available in the figure legends.<br><br>Have you included all the information requested in your manuscript?	Yes
**Resources**<br><br>A description of all resources used, including antibodies, cell lines, animals and software tools, with enough information to allow them to be uniquely identified, should be included in the Methods section. Authors are strongly encouraged to cite Research Resource	Yes

Identifiers (RRIDs) for antibodies, model organisms and tools, where possible.<br><br>Have you included the information requested as detailed in our Minimum Standards Reporting Checklist?	
**Availability of data and materials**<br><br>All datasets and code on which the conclusions of the paper rely must be either included in your submission or deposited in publicly available repositories (where available and ethically appropriate), referencing such data using a unique identifier in the references and in the “Availability of Data and Materials” section of your manuscript.<br><br>Have you have met the above requirement as detailed in our Minimum Standards Reporting Checklist?	Yes

Manuscript_revision Click here to access/download;Manuscript;Manuscript_revision.docx

Telomere-to-telomere genome assembly of *Electrophorus electricus* provides insights into the evolution of electric eels

Zan Qi[1,†], Qun Liu[2,†], Haorong Li[1,†], Yaolei Zhang[2], Ziwei Yu[1], Wenkai Luo[1], Kun Wang[1], Yuxin Zhang[1], Shoupeng Pan[1], Chao Wang[1], Hui Jiang[3], Qiang Qiu[1], Wen Wang[1], Guangyi Fan[2,*], Yongxin Li[1,*]

1.School of Ecology and Environment, Northwestern Polytechnical University, Xi'an 710072, China

2.BGI-Qingdao, BGI-Shenzhen, Qingdao 266555, China

3.College of Life Sciences, Hainan Normal University, Haikou 571158, China.

[†]These authors contributed equally to this work.

[*]Corresponding authors: fanguangyi@genomics.cn (G. F.); yxli28science@sina.com (Y. L.).

Abstract

Background: Electric eels evolved remarkable electric organs that enable them to instantaneously discharge hundreds of volts for predation, defense, and communication. However, the absence of a high-quality reference genome has extremely constrained the studies of electric eels in various aspects.

Results: Using high-depth, multi-platform sequencing data, we successfully assembled the first telomere-to-telomere high-quality reference genome of *Electrophorus electricus*, which has a genome size of 833.43 Mb and comprises 26 chromosomes. Multiple evaluations, including N50 statistics (30.38 Mb), BUSCO scores (97.30%), and mapping ratio of short-insert sequencing data (99.91%) demonstrate the high contiguity and completeness of the electric eel genome assembly we obtained. Genome annotation predicted 396.63 Mb repetitive sequences and 20,992 protein-coding genes. Furthermore, evolutionary analyses indicate that Gymnotiformes, which the electric eel belongs to, has a closer relationship with Characiformes than Siluriformes, and diverged from Characiformes at 103.20 million years ago. Pairwise sequentially Markovian coalescent analysis found that the electric eel had a stable population size during the Chibanian stage in Pleistocene, but its population

dramatically decreased from the subsequent period still now. Furthermore, many regulatory factors related to neurotransmitters and classical signaling pathways during embryonic development were significantly expanded, which may provide insights into the potential genetic underpinnings of the exceptional ability of electric eels to discharge high-voltage electricity.

Conclusions: This study not only provided the first high-quality telomere-to-telomere reference genome of the electric eel, but also offers insights into the potential genetic mechanisms underlying the exceptional ability of electric eels to discharge high-voltage electricity.

Keywords: Electric eel, *Electrophorus electricus*, Telomere-to telomere, Genome assembly; Genome annotation; Evolution

Introduction

The natural principles of "Law of the Jungle" and "Survival of the Fittest" underscore the paramount importance of animals' abilities in predation and defense. As one of the oldest vertebrate groups, fishes, despite their rich biodiversity, largely adopt extremely conservative feeding and defense strategies that rely primarily on biting. However, after a long evolutionary process, strong electric fishes have abandoned traditional biting methods and instead use high-voltage electric shocks as their predation and defense strategy [1,2]. Nowadays, there are at least three main living groups of strongly electric fishes on Earth, including electric eel, electric catfish, and electric ray [1]. Among them, electric eels are the ones that boast the strongest electric shock capacity, mainly distributed in the Amazon River basin [2]. Previous studies have indicated that the ability of strongly electric fishes to instantly release high-voltage electricity is mainly attributed to their evolved new organ: electric organs [1]. Interestingly, besides the one strong electric organ that is shared by all three groups (known as the Main electric organ in electric eels; Main EO), electric eels have also evolved two additional weak electric organs: Hunter's electric organ (Hunter's EO) and Sach's electric organ (Sach's EO), which are mainly used to sense the surrounding environment and for communication [3]. Anatomical and electrophysiological studies have revealed that discharge

cells, specifically known as electrocytes, constitute a substantial part of the bodies of strongly electric fishes [1,4]. Their unique serial battery-like arrangement of electrocytes within their electric organs (EOs) is the underlying factor enabling these strongly electric fish to instantaneously discharge hundreds of volts of electricity [1]. For that reason, the body length of strongly electric fishes determines the voltage they can generate [4]. According previous records, the adult electric eel can instantaneously discharge approximately 600-800 volts of high-voltage electricity [2]. Therefore, it becomes particularly crucial to conduct comprehensive and systematic studies on the remarkable innovative characteristics of electric eels, especially exploring the composition of their electric organs and the mechanisms of their high-voltage discharge.

In recent years, the rapid development of genome sequencing technology has significantly expedited the study progress across various life science disciplines. Excitingly, the emergence of long-read sequencing technologies, notably Oxford Nanopore Technologies (ONT) and Pacific Biosciences (PacBio), has presented an opportunity to assemble genomes up to the telomere-to-telomere level. Still now, several important species have achieved telomere-to-telomere level assembly, and many complex questions have been solved with the help of the high-quality reference genomes [5]. However, only a limited number of animal species, such as human, rodent, chicken and zig-zag eel [6-9], have been reported to have achieved telomere-to-telomere level assembly. During its long evolutionary process, the electric eel has developed numerous fascinating and unique biological traits [1,3,4]. However, despite the availability of publicly accessible electric eel genome assemblies on NCBI (e.g., GCA_013358815.1), the contiguity and completeness of these genomes are often insufficient to meet the standards of high-precision and systematic comparative genomics research.

In this study, combining multiple sequencing data, we successfully assembled the first telomere-to-telomere high-quality reference genome of electric eel (*Electrophorus electricus*), with a genome size of 833.43 Mb and comprising 26 chromosomes. Multiple evaluations, including N50 statistics, BUSCO scores, and the mapping ratio of short-insert sequencing data, indicate the high contiguity and completeness of the electric eel genome assembly we obtained. Genome annotation identified 396.63 Mb repetitive sequences and 20,992 protein-coding genes. Evolutionary analyses indicate that Gymnotiformes, which the electric

eel belongs to, has a closer relationship with Characiformes than Siluriformes, and diverged from Characiformes at 95 million years ago. Pairwise sequentially Markovian coalescent analysis found that the electric eel had a stable population size during the Chibanian stage in Pleistocene, but its population dramatically decreased from the subsequent period. Furthermore, many regulatory factors related to neurotransmitters and classical signaling pathways during embryonic development were significantly expanded, which may provide insights into the potential genetic underpinnings of the exceptional ability of electric eels to discharge high-voltage electricity. This study presents the first high-quality telomere-to-telomere reference genome of electric eel, marking a significant milestone and opening up valuable opportunities for future comprehensive studies of the exceptional characteristics of strongly electric fishes.

Methods

Sampling, library construction, and sequencing

An individual electric eel (*E. electricus*) used in this study was procured from the aquatic pet market in China. Fresh tissues were dissected and subsequently sent to the biological company of Benagen (Wuhan, China) and Novogene (Beijing, China) for a range of genomic analyses. These included DNA extraction, library construction, and whole-genome sequencing, utilizing various sequencing technologies. Specifically, ultra-long genome sequencing was conducted using the Oxford Nanopore Technologies (ONT) platform, while HiFi sequencing employed the Pacific Biosciences (PacBio) platform. Additionally, Hi-C sequencing and short-insert sequencing were performed on the Illumina platform. All experimental operations with animals adhered to relevant standards of animal ethics and welfare of Northwestern Polytechnical University.

Quality control of raw sequencing data

For the short-insert reads generated from the Illumina platform, all low-quality reads/bases, duplicated reads, and adapter sequences were filtered out using Perl scripts. For Nanopore long reads, we calculated the mean quality score for each read, retaining only those that met the criteria of have a mean quality score of ≥ 7 and a length of ≥ 1 Kb). For PacBio long

reads, CCS (v6.0.0) was used to remove low-quality reads, applying the parameters of "-min-passes 3 -min-length 10 -min-rq 0.99".

Estimation of genome size

To investigate the genome characteristics of *E. electricus*, a *k-mer*-based approach was implemented utilizing the cleaned short-insert sequencing reads obtained from the Illumina platform. The genome size (G) can be estimated using the formula: $G = TN_{17\text{-mer}}/PFD_{17\text{-mer}}$, where $TN_{17\text{-mer}}$ denotes the total number of 17-mers and $PFD_{17\text{-mer}}$ represents the peak frequency depth of the 17-mers. Specifically, the 17-mers were counted using KmerFreq (v1.0) (https://github.com/fanagislab/kmerfreq) with the parameters "-k 17" and then these data were used to estimate the genome size by running the GenomeAnalysis.pl script. In addition, the genome size has also been evaluated by GCE (v1.0.2) [10] using 17-mers.

Genome assembly

To achieve a high-quality genome assembly of *E. electricus*, a multi-step assembly strategy was employed. 1) The contig-level genome was assembled using Hifiasm (v0.19.5-r592) [11] based on both HiFi reads and ultra-long sequencing reads, with the default parameters except for setting the "-D" option to 10. 2) Potential base errors generated during the sequencing process in the contig-level genome were corrected using Pilon (v1.22) [12] with default parameters, based on the short-insert sequencing reads. 3) The contigs of the corrected genome assembly were extended using Lrscaf (v1.1.10) [13] with default parameters except "-t mm", based on the ultra-long sequencing reads. 4) The extended contig-level genome was anchored into chromosomes based on the analysis of Hi-C sequencing data using Juicer (v1.6) [14] and 3D *de novo* assembly (v170123) [15] workflow with the parameters of "-m haploid -i 15000 -r 2". 5) The ultra-long sequencing reads generated from the ONT platform were assembled into a contig-level genome using NextDenovo (v2.5.2) [16], with the parameters of "read_type = ont, seed_cutoff = 109,337, read_cutoff = 1k, minimap2_options_cns = -x ava-ont -t 15 -k17 -w17". 6) Potential base errors generated during the sequencing process in the contig-level genome were corrected using NextPolish (v1.4.1) [17], based on HiFi reads and clean short reads, with the parameters of "sgs

options=-max_depth 100, HiFi options=-max_depth 150, HiFi minimap2 options=-x map PB". 7) The gaps in the chromosome-level genome assembly were filled using TGS-gapcloser (v1.2.1) [18], based on the corrected ultra-long contig-level assembly, with the parameters of “--min_nread 1 --min_match 2000 --minmap_arg '-x asm5'”. 8) Potential base errors in the gap-closed genome assembly were further corrected using Pilon (v1.22) [12] with default parameters, based on the short-insert sequencing reads.

Quality evaluation of genome assembly

Multiple strategies were employed to evaluate the quality of the genome assembly. 1) The completeness of conserved core genes in the actinopterygii database was analyzed for the genome using BUSCO (v5.4.5) [19]. 2) The cleaned short-insert sequencing reads generated on the Illumina platform were aligned to the genome with BWA (v0.7.17) [20] using the parameters of "bwa mem -M", and the proportion of properly mapped reads was determined using the *flagstat* function of SAMTools (v1.9) [21]. 3) The contiguity of the genome was evaluated using the N50 score, which was calculated with a custom Perl script.

Annotation of repetitive sequences

To identify the repetitive sequences in the *E. electricus* genome, including tandem repeats and transposable elements (TEs), we integrated a homology-based prediction using the Repbase library and a *de novo* prediction based on self-sequence alignment and repetitive sequence features. First, tandem repeats were annotated using Tandem Repeat Finder (v4.10) [22] with the parameters of “Match = 2, Mismatch = 7, Delta = 7, PM = 80, PI = 10, Minscore = 50, MaxPeriod = 2000 -d -h”. Second, TEs were predicted on both DNA and protein levels. On the DNA level, RepeatModeler software (v2.0.1, https://www.repeatmasker.org/RepeatModeler) was used to construct the *de novo* repeat library. RepeatMasker (v4.0.5) [23] was then run separately against the *de novo* library and the repbase library to identify repetitive sequences with parameters of “-nolow -no_is -norna”. Third, on the protein level, RepeatProteinMask (v1.36) was used to search TEs in its protein database with the parameters of “-noLowSimple -pvalue 0.0001”. Finally, the annotation results generated from different annotation strategies were were integrated to produce the

final annotation of repetitive sequences. The telomere and centromere regions were predicted according to the quarTeT (v1.1.8) [24].

Annotation of protein-coding genes

Multiple strategies, including the *de novo*-based prediction, homology-based prediction, and transcript-based prediction, were employed for annotating the protein-coding genes of *E. electricus* genome. 1) For *de novo*-based prediction, BRAKER3 [25] was employed with default parameters based on the assembled transcripts. 2) For homology-based prediction, protein sequences from ten species, including *Clarias gariepinus* (GCF_024256425.1), *Hemibagrus wyckioides* (GCF_019097595.1), *Ictalurus punctatus* (GCF_001660625.3), *Mus musculus* (GCF_000001635.27), *Pangasianodon hypophthalmus* (GCF_027358585.1), *Silurus meridionalis* (GCF_014805685.1), *Tachysurus fulvidraco* (GCF_022655615.1), *Tachysurus vachellii* (GCF_030014155.1) and *Danio rerio* (GCF_000002035.6), were downloaded from NCBI database. All downloaded genes were aligned to the genome using BLAST (v2.6.0) [26] with the parameters of "e-value 1e-5 -p tblastn -m 8". Genewise (v2.2.0) [27] was used to identify the longest coding regions and/or highest score in each gene locus to support the presence of a homologous gene with the parameters of "-tfor -pseudo -pretty -sum -gff -genesf". 3) For transcript-based prediction, the coding regions were first *de novo* assembled utilizing the Hisat2 (v2.2.1) and StringTie (v2.1.4) workflow [28,29], both of which were employed with default parameters using our previous RNA-seq data (PRJNA592729). Subsequently, TransDecoder (v5.5.0, http://transdecoder.sourceforge.net) was employed to predict transcripts. These transcripts were then mapped onto the reference genomes using BLAT (v36) [30] and the gene structure was predicted by GeneWise (v2.2.0) [27] with default parameters. Finally, the results generated from these three strategies were integrated into a final gene set using EvidenceModeler (v.1.1.1) [31] with the parameters of "--segmentSize 5000000 --overlapSize 50000".

Functional annotation of protein-coding genes

To enhance the understanding of the predicted genes, all protein-coding genes were aligned against multiple databases for functional annotation. These databases include Gene Ontology

(GO: http://geneontology.org), the Integrated Resource of Protein Domains and Functional Sites (InterPro: https://www.ebi.ac.uk/interpro), the Kyoto Encyclopedia of Genes and Genomes (KEGG: https://www.kegg.jp), SwissProt (www.uniprot.org), TrEMBL (www.uniprot.org), and the non-redundant protein database (NR: https://ftp.ncbi.nlm.nih.gov/blast/db/). The alignment to the InterPro database was performed using InterProScan (v5.45-80.0) [32] with the parameters "-dp -f tsv -iprlookup -goterms". For the other annotation processes, BLAST (v2.6.0) [26] was utilized with the parameters "-b 100 -v 100 -p blastp -e 1e-05 -F F". For each gene, the annotation term with the highest score was retained as the final functional annotation.

Identification of orthologous genes

Orthologous genes among seven species, including *D. rerio* (GCF_000002035.6), *Ictalurus punctatus* (GCF_001660625.3), *Pygocentrus nattereri* (GCF_015220715.1), *Tachysurus fulvidraco* (GCF_022655615.1), *Astyanax mexicanus* (GCF_023375975.1), *Trichomycterus rosablanca* (GCF_030014385.1), and *E. electricus*, were identified for the comparative genomic analyses. First, the longest transcript for each gene was solely retained among these species with custom Perl script. Second, the reciprocal best BLAST hit of each gene pairs was employed using BLAST [26] with the parameters of "-evalue 1e-5 -outfmt 6". Third, pairwise orthologous relationships were identified among these species using OrthoMCL (v2.0.9) [33] with the default parameters.

Inference of phylogenetic relationships

The protein sequences of the single-copy orthologous genes, identified among the seven species, were aligned using MUSCLE (v3.8.31) [34] with default parameters. Then, using *D. rerio* as the outgroup species, we constructed phylogenetic trees for each gene using RAxML (v8.2.10) [35] with the parameters of "-f a -m PROTGAMMAAUTO -p 12345 -T 30 -x 12345 -N 100" and IQ-TREE (v2.2.0) [36] with the parameters of "-m JTT+C60+F -msub nuclear -B 1000 -alrt 1000 --seqtype AA", respectively. Finally, the species tree was inferred by ASTRAL (v5.7.1) [37] with default parameters based on the constructed gene trees.

Inferrence of divergence time

To accurately estimate the divergence times among species, we employed an approach that integrated the phylogenetic tree, 4dTVs (fourfold degenerate synonymous sites) data extracted from identified single-copy orthologous genes, and fossil-calibrated information sourced from the TIMETREE database (http://www.timetree.org). This multifaceted dataset was then utilized within the MCMCtree model, implemented in PAML (v4.4) [38], to infer the divergence times.

Relative evolutionary rate of species

To compare the relative evolutionary rates between *E. electricus* and other fish species, we first concatenated the sequences of single-copy orthologous genes into a supergene for each species. Subsequently, we performed multiple sequence alignment of these supergenes using MUSCLE (v3.8.31) [34] with default parameters. Finally, we analyzed the relative evolutionary rates of these species using the LINTRE program (v1.1) [39], designating *E. electricus* as the reference species and *D. rerio* as the outgroup species.

Dynamic change of population history

To obtain a comprehensive understanding of the population status of *E. electricus*, we conducted an analysis of the dynamic changes in its population history spanning the recent past. First, the short-insert sequencing data was mapped to the reference genome using BWA (v0.7.17) [20] with the command 'bwa mem -M'. Second, SAMtools (v1.13) [21] converted the aligned results to *bam* format with 'view -bS', sorted the resulting *bam* file using 'samtools sort', and created an index for the sorted file with 'samtools index'. Third, SNPs were detected using BCFtools (v1.16) [40] through the sequential commands 'bcftools mpileup -d 150 -q 20 -Q 20' for pileup generation and 'bcftools call -c' for variant calling. Following detection, the format of the resulting *VCF* file was refined using the *vcfutils.pl* script from BCFtools (v1.16) [40]. Based on these results, a final PSMC (v0.6.5-r67) analysis [41] was conducted with 100 bootstrap replicates, utilizing the parameters "-N 25 -r 5 -p '4+25*2+4+6'". The nucleotide substitution rate for this species, measured as substitutions per site per million years, was estimated using fourfold degenerate sites and fossil information via the r8s software (v1.2)

[42]. The per-generation mutation rate was then estimated by multiplying the per-nucleotide substitution rate by the generation time. Finally, these results were scaled to absolute time and population size using the generation time and estimated per-generation mutation rate. This was accomplished by first running *psmc2history.pl* with the default parameters, and then using the acquired values for the -*g* (generation time) and -μ (mutation rate per generation) parameters with *history2ms.pl* to perform the scaling.

Expansion and contraction of gene family

Based on the results of gene families identified by OrthoMCL (v2.0.9) [33] and the divergence-timed phylogenetic tree derived from PAML (v4.4) [38], we employed the random birth-and-death model in CAFE (v4.2.1) [43] to investigate the dynamics of gene family expansions and contractions. If the copy number of the gene family in the detected branch lineage was higher/lower than that of its most recent common ancestral branch, then the gene family was defined as being substantially expanded/contracted in the detected lineage.

Functional enrichment

To carry out functional enrichment analysis, such as the GO enrichment, we adopted a workflow that integrated EggNOG mapper software (v2.1.12) [44] and clusterProfiler (v4.6.2) [45]. Functional annotation of the whole gene set of *E. electricus* was conducted using the EggNOG mapper software (v2.1.12), leveraging the EggNOG database (v5.0). From these annotations, unique identifier numbers for each gene in the GO database were extracted. Subsequently, two files were prepared: an interest gene list and a total gene list, both adhering to the specified format. The interest gene list contained the gene IDs of genes of interest, while the total gene list included all gene IDs along with their corresponding GO IDs. Functional enrichment analysis was then performed using the enrichGO functions within the clusterProfiler R package (v4.6.2) [45].

Results

High-quality reference genome assembly of electric eel

The evolution of electric organs in electric eels, featuring a main electric organ (Main EO)

and two additional weak electric organs (Hunter's EO and Sach's EO), represents one of their most innovative and distinctive characteristics (**Fig. 1**). Therefore, a genome of the electric eel with higher contiguity and completeness is urgently needed. To investigate the genomic characteristics of *Electrophorus electricus* (electric eel), we generated a substantial amount of short-insert sequencing reads (97.17 Gb) using the Illumina platform (**Supplementary Table S1**). Our K-mer analysis identified a prominent main peak (homozygous peak) at a kmer depth of 83, and we calculated the genome size of *E. electricus* to be close to 800 Mb (**Supplementary Table S2 and Supplementary Fig. S1**). To facilitate the assembly of a high-quality reference genome of *E. electricus*, we further generated diverse sequencing data from multiple platforms, including Oxford Nanopore ultra-long reads (46.31 Gb), PacBio HiFi reads (59.65 Gb), and Illumina Hi-C reads (225.59 Gb) (**Supplementary Table S1**). Considering the respective advantages of these two types of long-read sequencing data, we assembled the contig-level genome using Hifiasm [11] by simultaneously utilizing both types of data (HiFi reads and ultra-long reads), resulting in an 817.93 Mb genome assembly with an N50 length of 21.44 Mb and average contig length of 1.56 Mb (**Supplementary Table S3**). Furthermore, we refined the assembly by correcting potential base errors using short-insert sequencing data and further extended the sequence length with ultra-long reads, which remarkably improved the contiguity of *E. electricus* genome (N50 length: 21.44 Mb; average contig length: 1.72 Mb, **Supplementary Table S3**). To further achieve a chromosome-level genome assembly, we anchored the extended contig-level genome into chromosomes using 3D-DNA software (v170123) [15], based on high-depth Hi-C sequencing reads (**Supplementary Table S1**). This resulted in a genome assembly of 823.20 Mb, with 26 chromosomes were successfully assembled (**Supplementary Tables S3 and S4**). Subsequently, we filled the gaps in the chromosome-level genome assembly by utilizing the polished contig-level assembly, which was generated solely from ultra-long reads. Finally, to remove potential base sequencing errors, we further polished the genome assembly after closing the gaps, based on Illumina short reads, resulting in a final 833.43 Mb genome of *E. electricus* (**Supplementary Table S4**). To comprehensively evaluate the quality of the *E. electricus* genome, we employed multiple evaluation strategies, including N50 length (30.38 Mb; **Table 1**), BUSCO scores (97.30%; **Table 2**), and the mapping ratio of short-insert

sequencing reads (99.91%; **Supplementary Table S5**). These results indicate that we successfully obtained a high-contiguity and high-integrity genome assembly for the electric eel (**Table 1**).

Genome annotation of electric eel

To comprehensively understand the genome composition, such as repetitive sequences and protein-coding genes, we performed the genome annotation based on multiple strategies. After that, a total of 396.63 Mb of repetitive sequences were predicted, accounting for 47.59% of the *E. electricus* genome (**Supplementary Table S6**). Specifically, transposable element (TE) statistics reveal that DNA transposons are the most abundant type, accounting for 12.03% of the genome assembly with a total size of 100.26 Mb. Subsequently, long interspersed nuclear elements (LINEs) comprise 11.67% of the genome, totaling 97.26 Mb. Long terminal repeats (LTRs) occupy 4.65% of the genome, with a size of 38.76 Mb, while short interspersed nuclear elements (SINEs) constitute only 0.99%, amounting to 8.22 Mb in total (**Supplementary Table S7**). Furthermore, using three different annotation strategies, we successfully predicted 20,992 protein-coding genes, and 97.19% of the predicted genes were successfully annotated in public databases (**Supplementary Table S8**). Besides, the quality of the predicted genes is comparable to that of the model animal zebrafish in various aspects, including CDS length, exon length, and intron length (**Supplementary Fig. 4**), indicating that a high-quality protein-coding gene set has been obtained. The distributions of the genomic elements, including the protein-coding genes, tandem repeats (TRs), LTRs, LINEs, SINEs, DNA elements, and GC content, were shown in the circos plot (**Fig. 2**). Finally, we identified 46 telomeres and 26 centromeres in the chromosome-level genome, indicating that we have successfully obtained a high-quality reference genome for *E. electricus*, with many chromosomes achieving the telomere-to-telomere assembly (**Fig. 3**).

Reconstruction the evolutionary histories of electric eel

To enhance our understanding of electric eels, we first conducted a reciprocal BLAST hit analysis utilizing OrthoMCL (v2.0.9) [33], resulting in the identification of 3,548 single-copy orthologous genes shared between the electric eel and six other closely related fish species (*D.*

rerio, *I. punctatus*, *P. nattereri*, *T. fulvidraco*, *A. mexicanus*, and *T. rosablanca*), five of which belong to the superorder Characiphysae, similar to the electric eel (**Supplementary Fig. S3**). Previous studies had suggested that Gymnotiformes has a closer relationship with Siluriformes than with Characiformes [1]. However, it remained unclear whether this phylogenetic relationship could be confirmed on a whole-genomic scale analysis. To address this question, we analyzed the phylogenetic relationships among the seven species, with zebrafish as the outgroup species, using multiple methods, such as the species trees constructed based on different models (homogeneous model, non-homogeneous model). All results showed that the electric eel was clustered with *A. mexicanus* and *P. nattereri* in one branch (**Fig. 4; Supplementary Figs. S4 and S5**), indicating that Gymnotiformes has a closer relationship with Characiformes than with Siluriformes. Moreover, using the extracted fourfold degenerate sites from the single-copy orthologous genes, we employed the divergence time analysis and the result showed that the electric eel diverged from the ancestor of *A. mexicanus* and *P. nattereri* approximately 95.00 million years ago (Mya), which falls within the Upper Epoch of Cretaceous period (**Fig. 4**). Relative evolutionary rate analysis of species showed that electric eel has a faster evolutionary rate to the two Characiformes species (*A. mexicanus* and *P. nattereri*), whereas slower than the three Siluriformes species (*T. rosablanca*, *T. fulvidraco*, and *I. punctatus*), suggesting that electric eel faced a relative strong adaptive pressure than the two Characiformes species (**Fig. 5**). Interestingly, we also inferred the effective population size of *E. electricus* and found that the electric eel maintained a relatively stable population size until about 0.15 Mya, but there has been a sharp decline since then (**Fig. 6**).

Gene family expansion contributed to the unique traits of electric eel

To uncover the potential genetic basis for the unique traits of the electric eel, particularly the evolution of its electric organs, we conducted the gene family analysis. Our analysis identified 425 gene families that have undergone significant expansion in the genome of the electric eel (**Fig. 4**). Furthermore, we conducted functional enrichment analysis on these expanded gene families, and the results showed that the expanded gene families were primarily involved in the regulations of many core signaling pathways, such as Wnt (positive regulation of

canonical Wnt signaling pathway, $P = 2.96 \times 10^{-42}$; positive regulation of Wnt signaling pathway, $P = 1.23 \times 10^{-38}$; regulation of canonical Wnt signaling pathway, $P = 2.15 \times 10^{-26}$), SMO (positive regulation of smoothened signaling pathway, $P = 1.37 \times 10^{-63}$; smoothened signaling pathway, $P = 1.59 \times 10^{-51}$; regulation of smoothened signaling pathway, $P = 5.46 \times 10^{-46}$), and Notch signaling (positive regulation of Notch signaling pathway, $P = 1.48 \times 10^{-52}$; regulation of Notch signaling pathway, $P = 2.14 \times 10^{-32}$) (**Supplementary Table S9**). As we known, all these signalings are critical pathways during embryonic/organ development [46-48]. The expansion of regulatory/core genes in these pathways may provide more possibilities to evolve the new organs, especially the three electric organs. Interestingly, genes involved in the neurotransmitter catabolic process are also expanded in the *E. electricus* genome ($P = 1.56 \times 10^{-2}$) (**Supplementary Table S9**). Previous studies have suggested that when electric eel is stimulated by neurotransmitters, it will simultaneously open a large number of ion channels, leading to the formation of membrane potential difference and the release of high-voltage electricity [49,50]. The expansion of regulatory factors for neurotransmitters may significantly contribute to the electric eels' ability to rapidly respond and discharge high-voltage electricity when they need to attack or defend instantly. Taken together, our results provide insights into the potential genetic underpinnings of the exceptional ability of electric eels to discharge high-voltage electricity.

Discussion

Electric eels evolved substantial electric organs enable them to instantaneously discharge high-voltage electricity for predation, defense, and communication [1,51]. This study combined PacBio HiFi reads, Nanopore ultra-long reads, Illumina Hi-C reads, Illumina short-insert sequencing reads to assemble the telomere-to-telomere genome of electric eel. Compared to the existing electric eel genome on NCBI (GCA_013358815.1), the genome assembly generated in this study has a longer contig N50 and higher BUSCO score than the NCBI version **(Supplementary Table S10)**, indicating that our genome has higher contiguity and completeness. Furthermore, we revealed that the electric eel clusters with *A. mexicanus* and *P. nattereri*, suggesting a closer evolutionary relationship between Gymnotiformes and Characiformes than with Siluriformes, whereas the electric eel diverged from the common

ancestor of Characiformes approximately 95.00 Mya. However, the *E. electricus* exhibits a faster evolutionary rate compared to two Characiformes species, yet lags behind three Siluriformes species, suggesting that electric eel has faced stronger adaptive pressures than the two Characiformes species. Previous studies have rarely focused on the current status of electric eel populations, which is why our knowledge about the size and other information of electric eel populations is very limited. We analyzed the population history of *E. electricus* using the PSMC strategy and found that the population size of *E. electricus* was relatively stable in the Chibanian stage period of Pleistocene, but after that, the population decreased sharply, suggesting that the population has been subjected to significant impacts from potential factors in recent years, such as habitat destruction and human interference. Therefore, we hope for the immediate implementation of practical conservation measures. This includes conducting thorough research to understand the causes of the decline in electric eel populations, implementing protection strategies to safeguard their habitats, and raising public awareness about the crucial importance of preserving electric eel populations for biodiversity and ecosystem health. Besides, it is noteworthy that several studies have shown that the expansion of certain key gene families or the increase in gene copy number can significantly enhance specific functions or traits of species [52-54]. The remarkable long-distance electric discharge attack ability evolved by electric eels provide them with an absolute advantage for survival. To investigate whether the copy number of coding genes of electric eels underwent remarkable changes, we analyzed the gene families and found many regulatory factors related to neurotransmitters and classical signaling pathways during embryonic development were significantly expanded, which may contribute to the electric eels' ability to rapidly respond and discharge high-voltage electricity when they need to attack or defend instantly. Taken together, our study not only produced the first high-quality telomere-to-telomere reference genome for the electric eel, but also offers insights into the potential genetic mechanisms underlying the exceptional ability of electric eels to discharge high-voltage electricity.

Additional Files

Supplementary Table S1. Statistics of the sequencing data.

Supplementary Table S2. Estimation of genome size using GCE software.

Supplementary Table S3. Statistics of the intermediate results of genome assemblies.

Supplementary Table S4. Final assembly of the *E. electricus* genome.

Supplementary Table S5. Mapping ratio of the short reads to the genome assembly.

Supplementary Table S6. Statistics of the predicted repetitive sequences in *E. electricus* genome.

Supplementary Table S7. Statistics of transposable elements of *E. electricus* genome.

Supplementary Table S8. Functional annotation of the protein-coding genes in *E. electricus* genome.

Supplementary Table S9. GO enrichment analysis of the expanded gene families.

Supplementary Table S10. Comparison of the quality of the two genomes.

Supplementary Fig. S1. Genomic characteristics of *Electrophorus electricus*. The X-axis shows the k-mer depth, and the Y-axis shows the frequency of the k-mer for a given depth.

Supplementary Fig. S2. Comparative analysis of CDS, exon, and intron length distributions across species.

Supplementary Fig. S3. Identification of orthologous genes among the species.

Supplementary Fig. S4. Species tree construction with ASTRAL using RAxML-derived gene trees. The number at each node indicates the local posterior probability.

Supplementary Fig. S5. Species tree construction with ASTRAL using IQ-TREE-derived gene trees. The number at each node indicates the local posterior probability.

Abbreviations

BUSCO: Benchmarking Universal Single-Copy Orthologs; BLAST: Basic Local Alignment Search Tool; CDS: coding sequence; Gb: gigabase; Mb: megabase.

Competing Interests

The authors declared no competing interests.

Funding

This project was supported by the National Natural Science Foundation of China (31900312).

Data Availability

Genome assembly and sequencing data (including ONT long-reads, HiFi long-reads, Hi-C reads, and short-insert reads) have been uploaded to the China National GeneBank database (CNP0005951) and the National Center for Biotechnology Information (GCA_041902795.1). The genome annotation files are accessible through the DRYAD database (https://doi.org/10.5061/dryad.sqv9s4nc3). All additional supporting data are available in the GigaScience database [55].

Author Contributions

Y. L. and G. F. conceived and supervised the project. H. J. collected the samples. Z. Q., Q. L., H. L., Y. Z., S. P., and W. L. performed the bioinformatics analyses. W. W., Q. Q., K. W., and C. W. help the analyses and discussed the results. Z.Q. wrote the manuscript. Z. Q. and Y. L. revised the manuscript. All authors have read and approved the final manuscript.

References

1. Gallant J R, Traeger L L, Volkening J D et al. NONHUMAN GENETICS Genomic basis for the convergent evolution of electric organs. Science 2014;344(6191):1522-1525. http://doi:10.1126/science.1254432.
2. de Santana C D, Crampton W G R, Dillman C B et al. Unexpected species diversity in electric eels with a description of the strongest living bioelectricity generator. Nat Commun 2019;10(1):4000. http://doi:10.1038/s41467-019-11690-z.
3. Traeger L L, Sabat G, Barrett-Wilt G A et al. A tail of two voltages: Proteomic comparison of the three electric organs of the electric eel. Sci Adv 2017;3(7):e1700523. http://doi:10.1126/sciadv.1700523.
4. Schwassmann H O, Assunção M I & Kirschbaum F. Ontogeny of the electric organs in the electric eel, Electrophorus electricus: physiological, histological, and fine structural investigations. Brain Behav Evol 2014;84(4):288-302. http://doi:10.1159/000367884.
5. Li H & Durbin R. Genome assembly in the telomere-to-telomere era. Nat Rev Genet 2024. http://doi:10.1038/s41576-024-00718-w.
6. Nurk S, Koren S, Rhie A et al. The complete sequence of a human genome. Science 2022;376(6588):44-53. http://doi:10.1126/science.abj6987.
7. Brekke T D, Papadopulos A S T, Julià E et al. A New Chromosome-Assigned Mongolian Gerbil Genome Allows Characterization of Complete Centromeres and a Fully Heterochromatic Chromosome. Mol Biol Evol 2023;40(5). http://doi:10.1093/molbev/msad115.
8. Huang Z, Xu Z, Bai H et al. Evolutionary analysis of a complete chicken genome.

Proc Natl Acad Sci U S A 2023;120(8):e2216641120. http://doi:10.1073/pnas.2216641120.

9. Xue L, Gao Y, Wu M et al. Telomere-to-telomere assembly of a fish Y chromosome reveals the origin of a young sex chromosome pair. Genome Biol 2021;22(1):203. http://doi:10.1186/s13059-021-02430-y.
10. Liu B, Shi Y, Yuan J et al. Estimation of genomic characteristics by analyzing k-mer frequency in de novo genome projects. Quantitative Biology 2013;35(s 1–3):62-67.
11. Cheng H, Jarvis E D, Fedrigo O et al. Haplotype-resolved assembly of diploid genomes without parental data. Nat Biotechnol 2022;40(9):1332-1335. http://doi:10.1038/s41587-022-01261-x.
12. Walker B J, Abeel T, Shea T et al. Pilon: an integrated tool for comprehensive microbial variant detection and genome assembly improvement. PLoS One 2014;9(11):e112963. http://doi:10.1371/journal.pone.0112963.
13. Qin M, Wu S, Li A et al. LRScaf: improving draft genomes using long noisy reads. BMC Genomics 2019;20(1):955. http://doi:10.1186/s12864-019-6337-2.
14. Durand N C, Shamim M S, Machol I et al. Juicer Provides a One-Click System for Analyzing Loop-Resolution Hi-C Experiments. Cell Syst 2016;3(1):95-98. http://doi:10.1016/j.cels.2016.07.002.
15. Dudchenko O, Batra S S, Omer A D et al. De novo assembly of the Aedes aegypti genome using Hi-C yields chromosome-length scaffolds. Science 2017;356(6333):92-95. http://doi:10.1126/science.aal3327.
16. Hu J, Wang Z, Sun Z et al. NextDenovo: an efficient error correction and accurate assembly tool for noisy long reads. Genome Biol 2024;25(1):107. http://doi:10.1186/s13059-024-03252-4.
17. Hu J, Fan J, Sun Z et al. NextPolish: a fast and efficient genome polishing tool for long-read assembly. Bioinformatics 2020;36(7):2253-2255. http://doi:10.1093/bioinformatics/btz891.
18. Xu M, Guo L, Gu S et al. TGS-GapCloser: A fast and accurate gap closer for large genomes with low coverage of error-prone long reads. Gigascience 2020;9(9). http://doi:10.1093/gigascience/giaa094.
19. Simão F A, Waterhouse R M, Ioannidis P et al. BUSCO: assessing genome assembly and annotation completeness with single-copy orthologs. Bioinformatics 2015;31(19):3210-3212. http://doi:10.1093/bioinformatics/btv351.
20. Li H & Durbin R. Fast and accurate short read alignment with Burrows-Wheeler transform. Bioinformatics 2009;25(14):1754-1760. http://doi:10.1093/bioinformatics/btp324.
21. Li H, Handsaker B, Wysoker A et al. The Sequence Alignment/Map format and SAMtools. Bioinformatics 2009;25(16):2078-2079. http://doi:10.1093/bioinformatics/btp352.
22. Benson G. Tandem repeats finder: a program to analyze DNA sequences. Nucleic Acids Res 1999;27(2):573-580. http://doi:10.1093/nar/27.2.573.
23. Tarailo-Graovac M & Chen N. Using RepeatMasker to identify repetitive elements in genomic sequences. Curr Protoc Bioinformatics 2009;Chapter 4:4.10.11-14.10.14. http://doi:10.1002/0471250953.bi0410s25.

24. Lin Y, Ye C, Li X et al. quarTeT: a telomere-to-telomere toolkit for gap-free genome assembly and centromeric repeat identification. Hortic Res 2023;10(8):uhad127. http://doi:10.1093/hr/uhad127.
25. Gabriel L, Brůna T, Hoff K J et al. BRAKER3: Fully automated genome annotation using RNA-seq and protein evidence with GeneMark-ETP, AUGUSTUS and TSEBRA. bioRxiv 2024. http://doi:10.1101/2023.06.10.544449.
26. Altschul S F, Gish W, Miller W et al. Basic local alignment search tool. J Mol Biol 1990;215(3):403-410. http://doi:10.1016/s0022-2836(05)80360-2.
27. Birney E, Clamp M & Durbin R. GeneWise and Genomewise. Genome Res 2004;14(5):988-995. http://doi:10.1101/gr.1865504.
28. Kim D, Paggi J M, Park C et al. Graph-based genome alignment and genotyping with HISAT2 and HISAT-genotype. Nat Biotechnol 2019;37(8):907-915. http://doi:10.1038/s41587-019-0201-4.
29. Shumate A, Wong B, Pertea G et al. Improved transcriptome assembly using a hybrid of long and short reads with StringTie. PLoS Comput Biol 2022;18(6):e1009730. http://doi:10.1371/journal.pcbi.1009730.
30. Kent W J. BLAT--the BLAST-like alignment tool. Genome Res 2002;12(4):656-664. http://doi:10.1101/gr.229202.
31. Haas B J, Salzberg S L, Zhu W et al. Automated eukaryotic gene structure annotation using EVidenceModeler and the Program to Assemble Spliced Alignments. Genome Biol 2008;9(1):R7. http://doi:10.1186/gb-2008-9-1-r7.
32. Jones P, Binns D, Chang H Y et al. InterProScan 5: genome-scale protein function classification. Bioinformatics 2014;30(9):1236-1240. http://doi:10.1093/bioinformatics/btu031.
33. Li L, Stoeckert C J, Jr. & Roos D S. OrthoMCL: identification of ortholog groups for eukaryotic genomes. Genome Res 2003;13(9):2178-2189. http://doi:10.1101/gr.1224503.
34. Edgar R C. MUSCLE: multiple sequence alignment with high accuracy and high throughput. Nucleic Acids Res 2004;32(5):1792-1797. http://doi:10.1093/nar/gkh340.
35. Stamatakis A. RAxML version 8: a tool for phylogenetic analysis and post-analysis of large phylogenies. Bioinformatics 2014;30(9):1312-1313. http://doi:10.1093/bioinformatics/btu033.
36. Minh B Q, Schmidt H A, Chernomor O et al. IQ-TREE 2: New Models and Efficient Methods for Phylogenetic Inference in the Genomic Era. Mol Biol Evol 2020;37(5):1530-1534. http://doi:10.1093/molbev/msaa015.
37. Mirarab S, Reaz R, Bayzid M S et al. ASTRAL: genome-scale coalescent-based species tree estimation. Bioinformatics 2014;30(17):i541-548. http://doi:10.1093/bioinformatics/btu462.
38. Yang Z. PAML 4: phylogenetic analysis by maximum likelihood. Mol Biol Evol 2007;24(8):1586-1591. http://doi:10.1093/molbev/msm088.
39. Takezaki N, Rzhetsky A & Nei M. Phylogenetic test of the molecular clock and linearized trees. Mol Biol Evol 1995;12(5):823-833. http://doi:10.1093/oxfordjournals.molbev.a040259.
40. Narasimhan V, Danecek P, Scally A et al. BCFtools/RoH: a hidden Markov model

approach for detecting autozygosity from next-generation sequencing data. Bioinformatics 2016;32(11):1749-1751. http://doi:10.1093/bioinformatics/btw044.

41. Li H & Durbin R. Inference of human population history from individual whole-genome sequences. Nature 2011;475(7357):493-496. http://doi:10.1038/nature10231.

42. Sanderson M J. r8s: inferring absolute rates of molecular evolution and divergence times in the absence of a molecular clock. Bioinformatics 2003;19(2):301-302. http://doi:10.1093/bioinformatics/19.2.301.

43. De Bie T, Cristianini N, Demuth J P et al. CAFE: a computational tool for the study of gene family evolution. Bioinformatics 2006;22(10):1269-1271. http://doi:10.1093/bioinformatics/btl097.

44. Cantalapiedra C P, Hernández-Plaza A, Letunic I et al. eggNOG-mapper v2: Functional Annotation, Orthology Assignments, and Domain Prediction at the Metagenomic Scale. Mol Biol Evol 2021;38(12):5825-5829. http://doi:10.1093/molbev/msab293.

45. Yu G, Wang L G, Han Y et al. clusterProfiler: an R package for comparing biological themes among gene clusters. Omics 2012;16(5):284-287. http://doi:10.1089/omi.2011.0118.

46. Taciak B, Pruszynska I, Kiraga L et al. Wnt signaling pathway in development and cancer. J Physiol Pharmacol 2018;69(2). http://doi:10.26402/jpp.2018.2.07.

47. Pandit T & Ogden S K. Contributions of Noncanonical Smoothened Signaling During Embryonic Development. J Dev Biol 2017;5(4). http://doi:10.3390/jdb5040011.

48. Zhou B, Lin W, Long Y et al. Notch signaling pathway: architecture, disease, and therapeutics. Signal Transduct Target Ther 2022;7(1):95. http://doi:10.1038/s41392-022-00934-y.

49. Gotter A L, Kaetzel M A & Dedman J R. Electrophorus electricus as a model system for the study of membrane excitability. Comp Biochem Physiol A Mol Integr Physiol 1998;119(1):225-241. http://doi:10.1016/s1095-6433(97)00414-5.

50. Levinson S R, Duch D S, Urban B W et al. The sodium channel from Electrophorus electricus. Annals of the New York Academy of Sciences 1986;479:162-178.

51. Wang Y & Yang L. Genomic Evidence for Convergent Molecular Adaptation in Electric Fishes. Genome Biol Evol 2021;13(3). http://doi:10.1093/gbe/evab038.

52. Schartl M, Woltering J M, Irisarri I et al. The genomes of all lungfish inform on genome expansion and tetrapod evolution. Nature 2024;634(8032):96-103. http://doi:10.1038/s41586-024-07830-1.

53. Wang Y, Li X Y, Xu W J et al. Comparative genome anatomy reveals evolutionary insights into a unique amphitriploid fish. Nat Ecol Evol 2022;6(9):1354-1366. http://doi:10.1038/s41559-022-01813-z.

54. Chen L, Qiu Q, Jiang Y et al. Large-scale ruminant genome sequencing provides insights into their evolution and distinct traits. Science 2019;364(6446). http://doi:10.1126/science.aav6202.

55. Qi Z, Liu Q, Li H, et al. Supporting data for “Telomere-to-telomere genome assembly of Electrophorus electricus provides insights into the evolution of electric

eels.” GigaScience Database 2024.

Table 1. Statistics of the *E.electricus* genome.

Term	Size/Number
Genome size (bp)	833,427,914
Number of chromosomes	26
Number of contigs	479
Number of scaffolds	460
Contig N50 (bp)	21,439,367
Scaffold N50 (bp)	30,383,234
Number of telomeres	46
GC percent (%)	41.5

Table 2. BUSCO assessment of the *E.electricus* genome.

Term	Number	Percentage (%)
Complete BUSCOs(C)	3,541	97.3
Complete and single-copy BUSCOs (S)	3,448	94.7
Complete and duplicated BUSCOs (D)	93	2.6
Fragmented BUSCOs (F)	34	0.9
Missing BUSCOs (M)	65	1.8
Total BUSCO groups searched	3,640	100

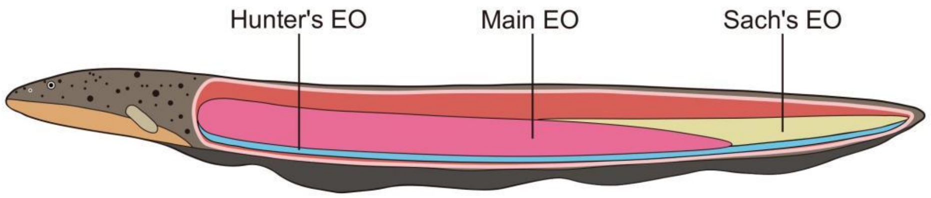

Fig. 1. Schematic diagram of the electric eel (*E. electricus*). Only the three electric organs were marked.

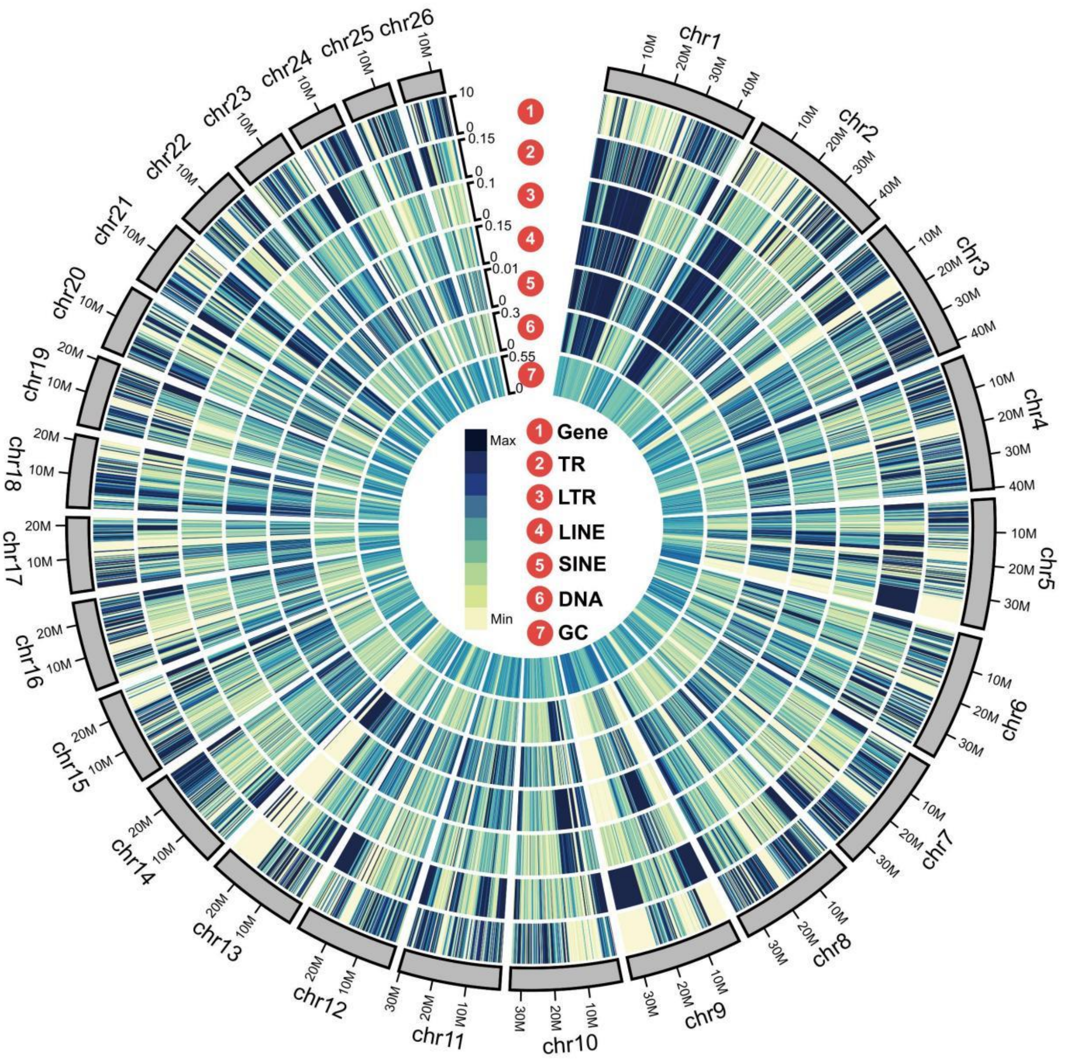

Fig. 2. Distributions of the genomic elements in *E. electricus*. In the circos plot, the outermost layer displays the distribution of protein-coding genes, followed by tandem repeats (TRs), long tandem repeats (LTRs), long/short interspersed nuclear elements (LINEs/SINEs), DNA elements, and finally, the GC content at the innermost layer. The color bar indicates the number/percent of each genomic element within the plot. As the color darkens, it signifies an increase in the percentage or number of that particular genomic element.

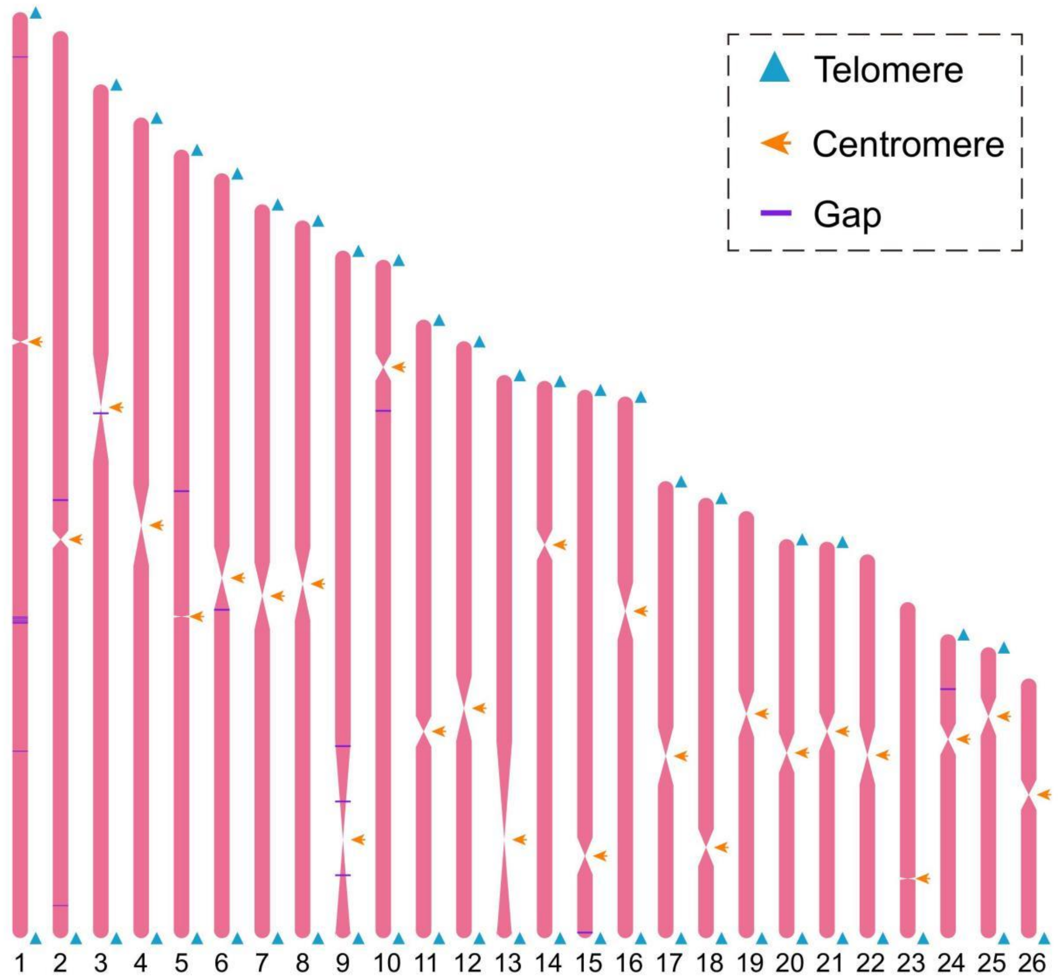

Fig. 3. Distributions of telomeres, centromeres, and gaps in the genome of *E. electricus*.

The specific meaning of each symbol is indicated in the figure.

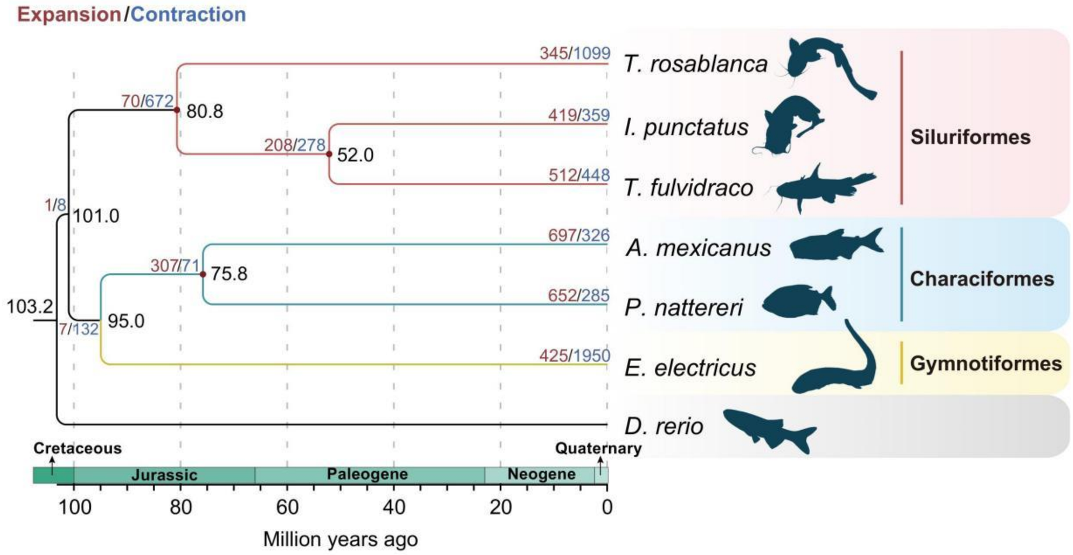

Fig. 4. Phylogenetic relationship and divergence time among the three orders. The red

dots at the nodes represent where fossil records were used for the calibration of divergence time. The black number at each node represents the divergence time between the two branches (Mya). The red and blue numbers at each node/species represent the number of expanded and contracted gene families, respectively. The coordinate axis below the phylogenetic tree shows the divergence time scale.

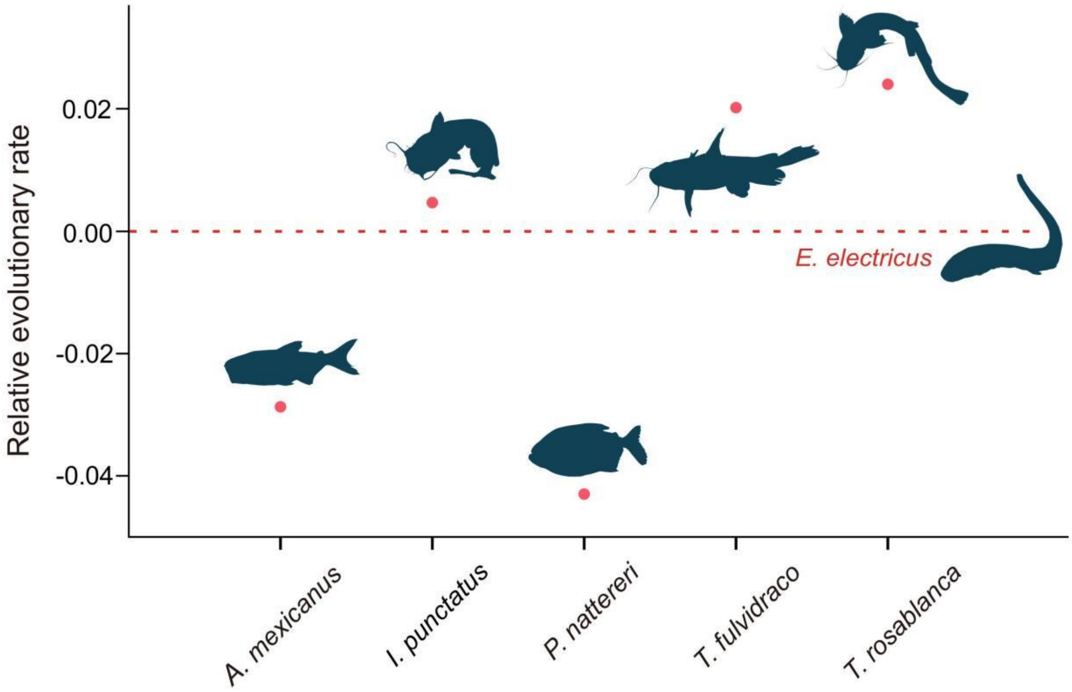

Fig. 5. Relative evolutionary rates of species. The analysis was performed using the single-copy orthologous genes with *E. electricus* as the reference species and zebrafish as the outgroup species. The y-axis shows the relative evolutionary rates of the species, and the black dots show the specific relative evolutionary rates for each species.

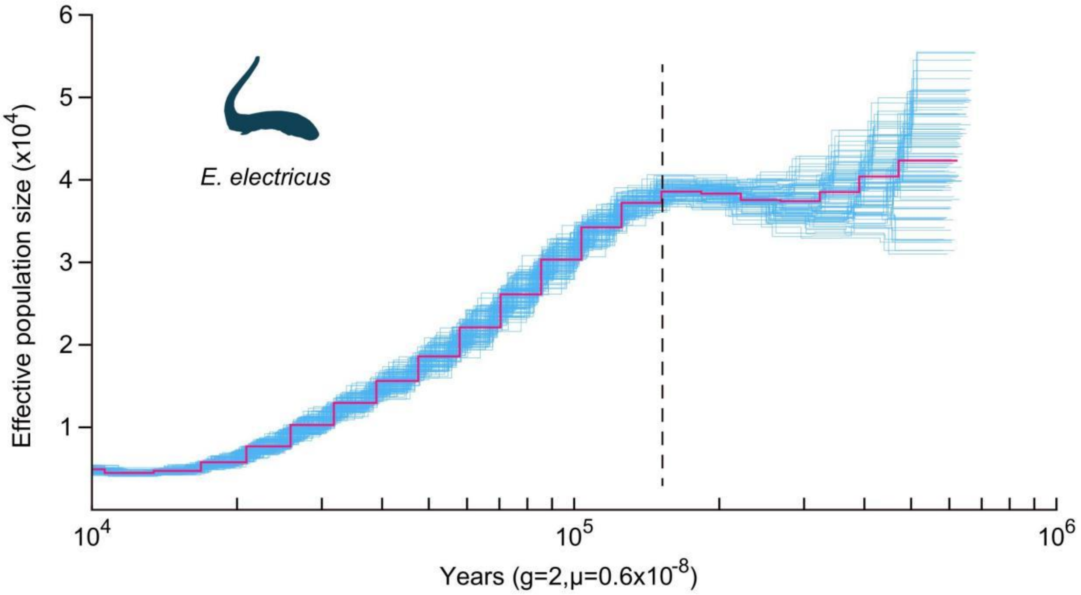

Fig. 6. Population history of *E. electricus*. The x-axis represents past years, and the y-axis represents the effective population size of the species; "g" is the generation time, and "μ" is the mutation rate of species.

Fig. 1

Click here to access/download;Figure;Fig. 1.pdf

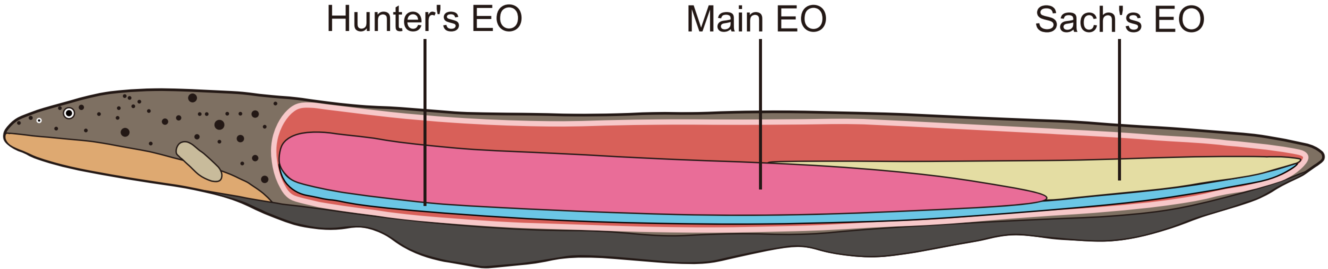

Fig. 2

Click here to access/download;Figure;Fig. 2.pdf

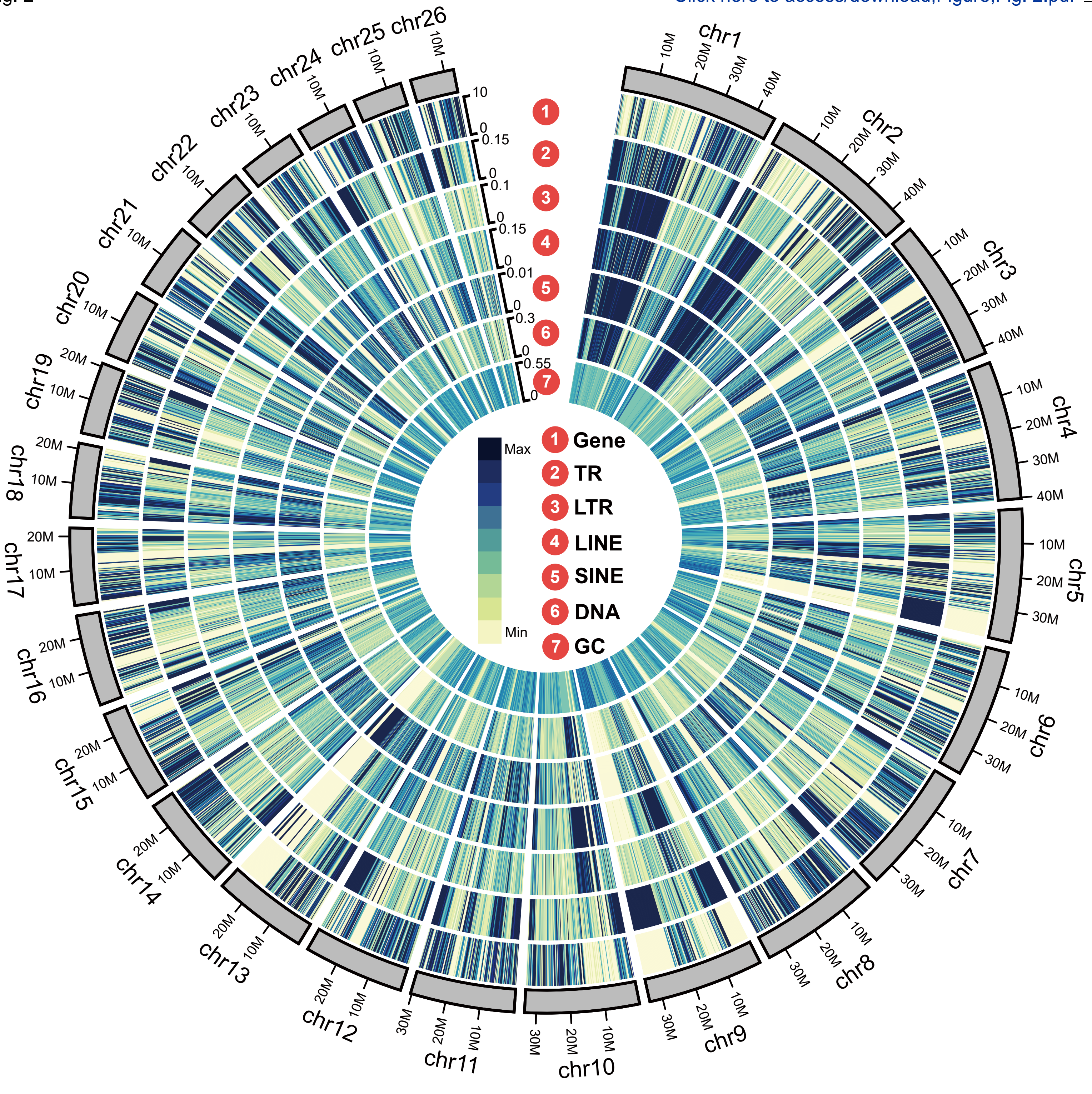

Fig. 3

Click here to access/download;Figure;Fig. 3.pdf

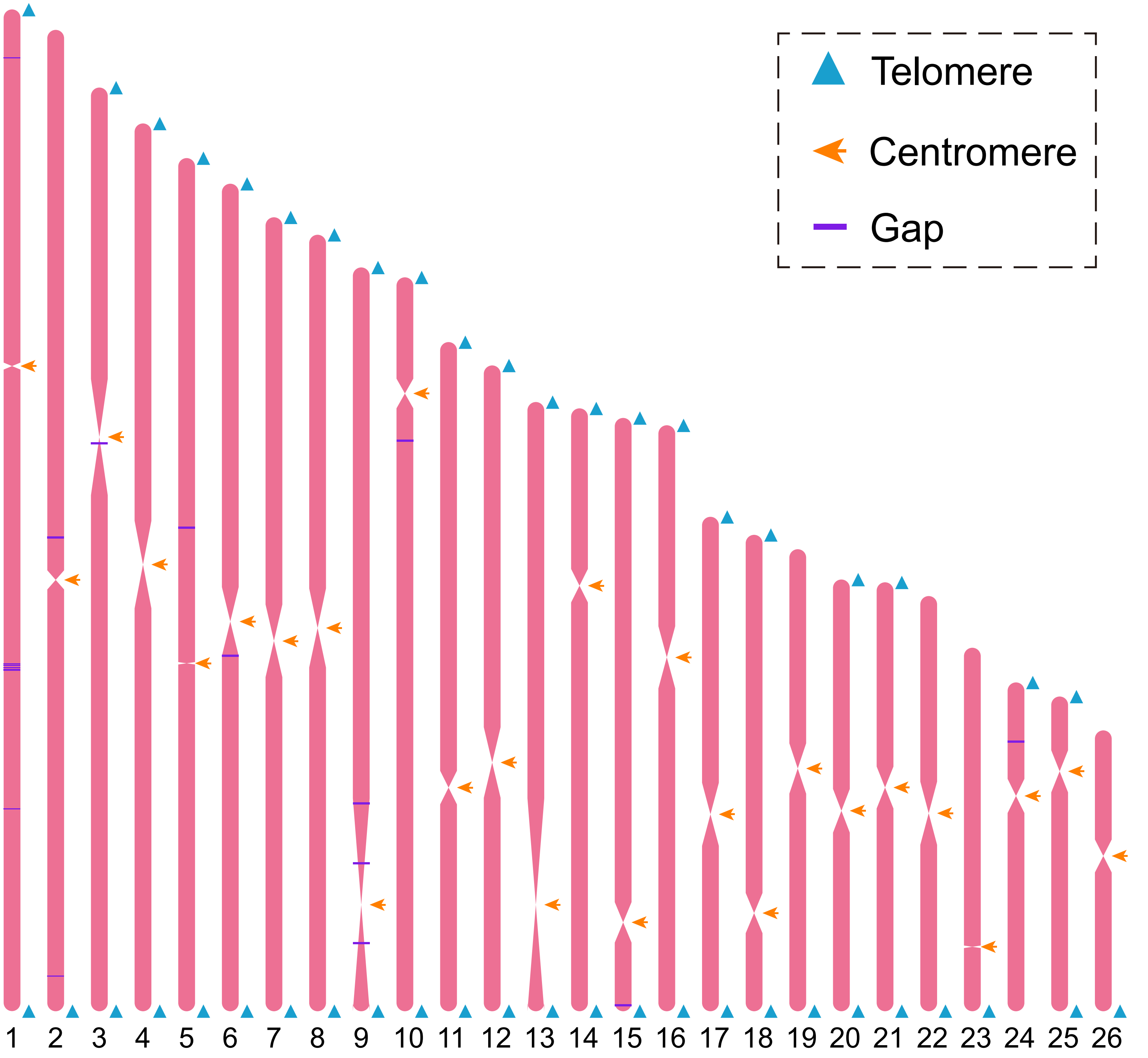

Fig. 4

Click here to access/download;Figure;Fig. 4.pdf

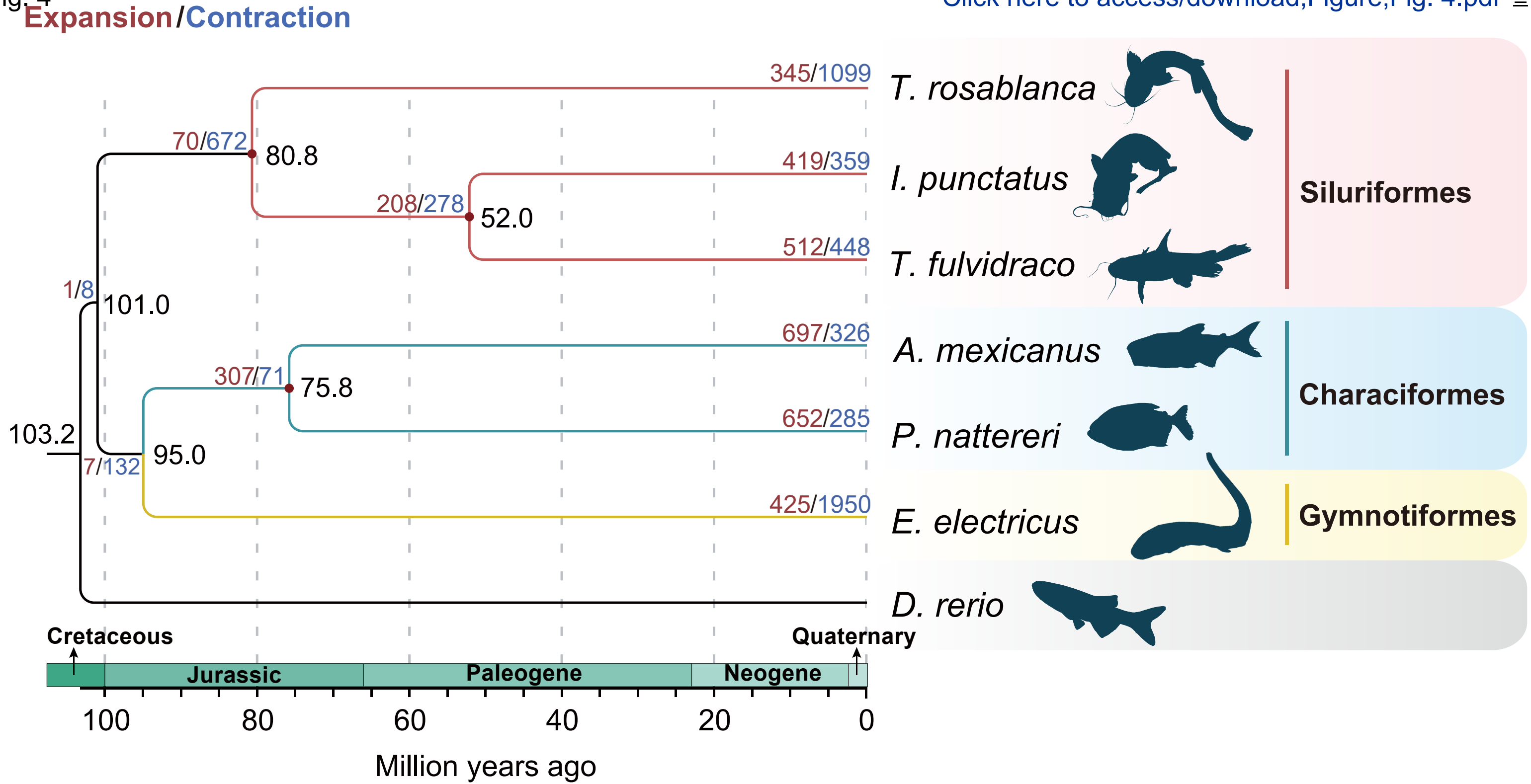

Fig. 5

Click here to access/download;Figure;Fig. 5.pdf

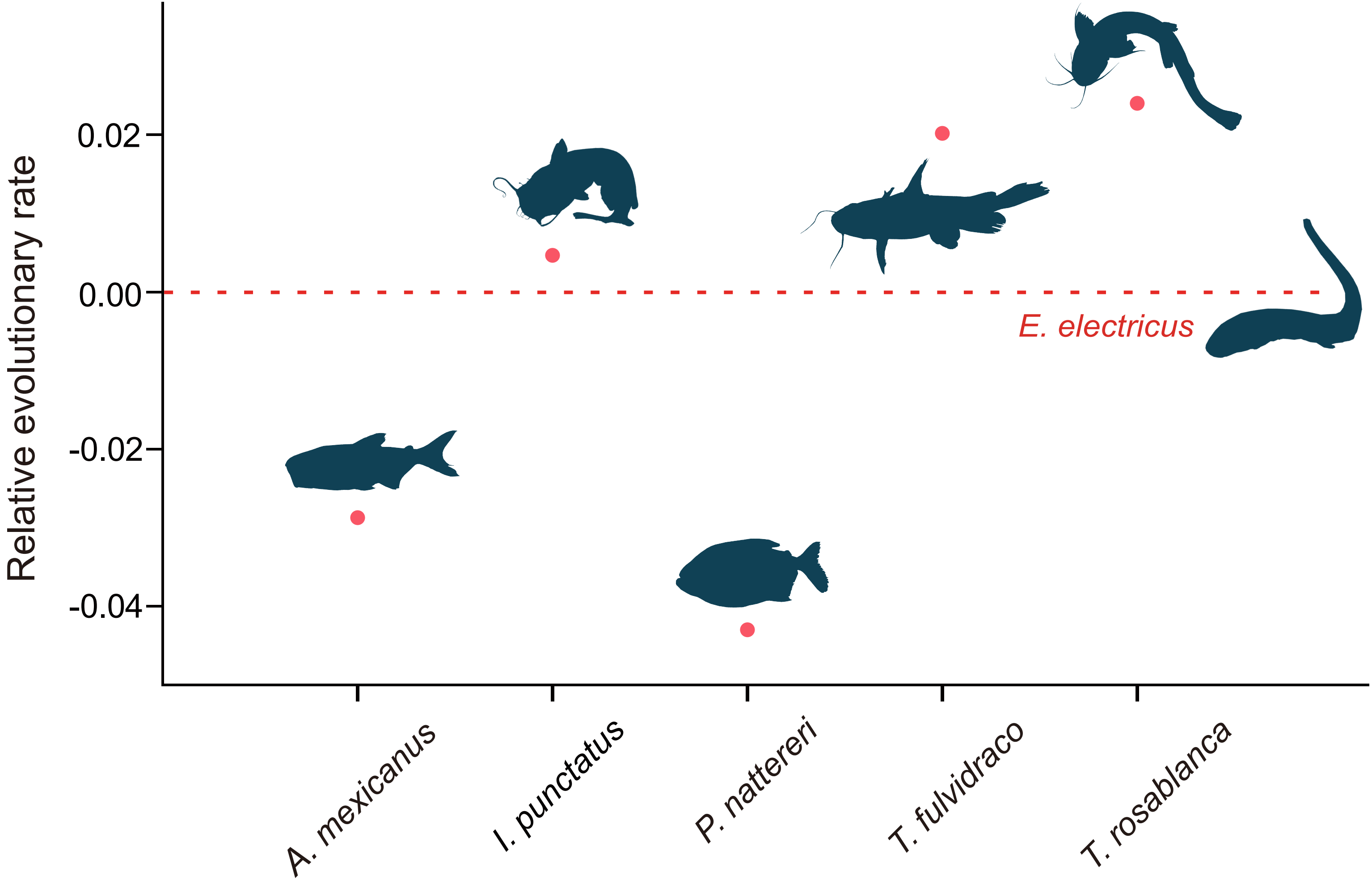

Fig. 6

Click here to access/download;Figure;Fig. 6.pdf

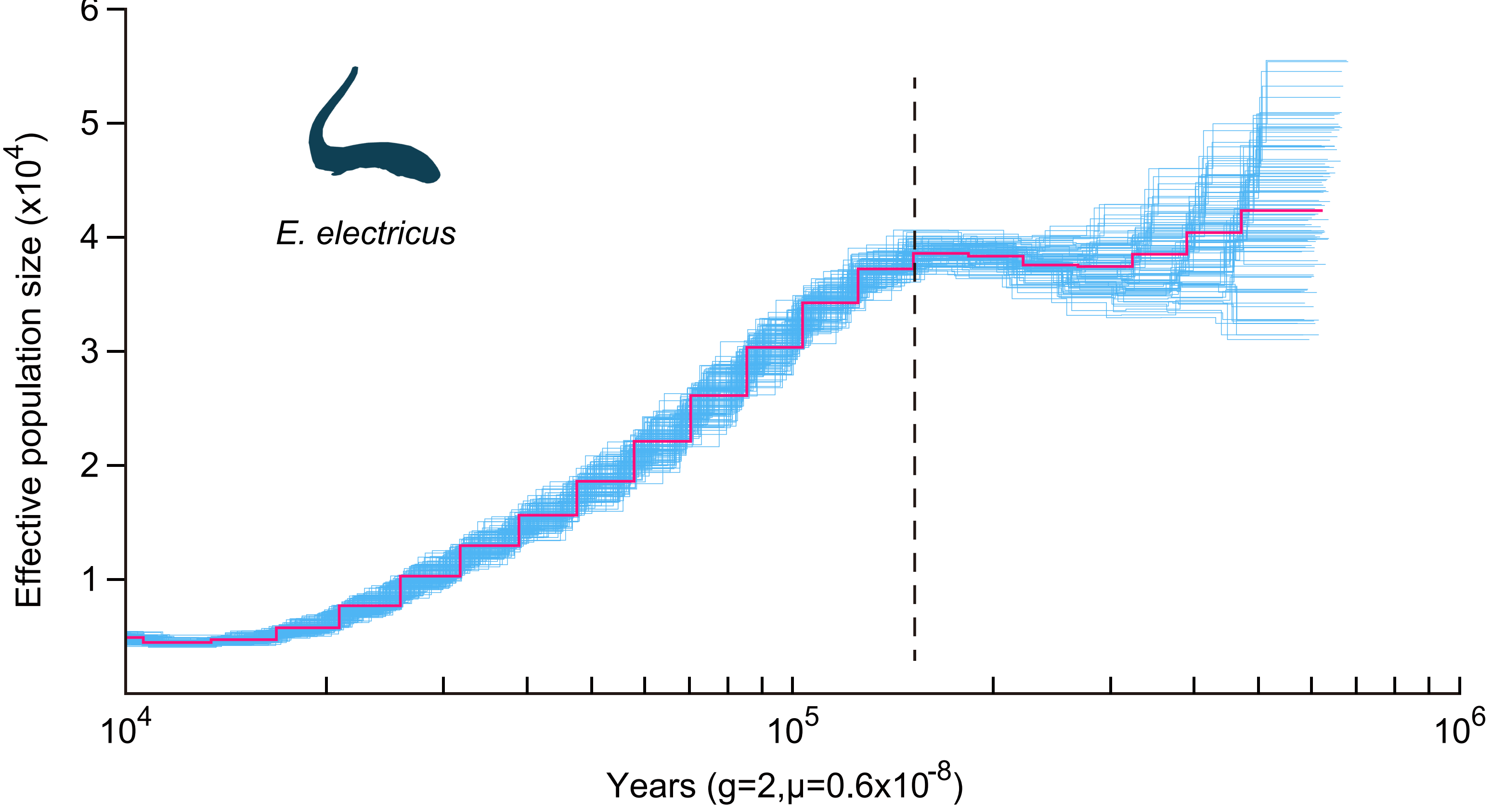

Supplementary file

Click here to access/download
Supplementary Material
Supplementary file_revision.docx
