# Supplementary material for: Telomere-to-telomere genome assembly of Electrophorus electricus provides insights into the evolution of electric eels
Source: Gigascience. 2025 Apr 1;14:giaf024. doi: 10.1093/gigascience/giaf024 (PMC11959694; doi:10.1093/gigascience/giaf024)
Supplement: giaf024_Response_to_Reviewer_Comments_Original_Submission [file giaf024_response_to_reviewer_comments_original_submission.pdf]

Author's Response To Reviewer Comments

Reviewer #1:
1. Qi et al. described a high-quality of E. electricus with many chromosomes reaching the T2T level. While this genome assembly can serve as an interesting resource for a fish T2T genome, very few novel insights have been revealed by the analysis.
Response: Thank you for recognizing the quality of our genome. The main goal of this work is to provide a higher-quality reference genome for the electric eel within the field, so that other scientists in the field can join us in our efforts to study the key scientific questions of electric eel. Therefore, we submitted this manuscript as a Data Note to this journal. For several key scientific questions, we have conducted systematic research using multiple sequencing data and experiment validation in 2-3 other works that we are currently preparing. Thank you for pointing out this issue.

2. There are at least three additional E. electricus genome assemblies available in NCBI. I am surprised that the authors mentioned none of them, nor comparing them with their assembly. Note the VGP E. electricus also has a high-quality with two haploid genomes assembled. Curiously, the genome sizes vary substantially among the assemblies: the VGP assembly has a ~600 Mb genome size, but the assembly from this study is larger than 800 Mb. Why?
Response: Thank you for your valuable suggestion. In this revision, we have added a description of the electric eel VGP genome in the Introduction and incorporated results comparing the quality of the electric eel VGP genome here in the Results section. Regarding the genome size estimation, we used two methods, which have been utilized in numerous high-quality publications, to estimate it, all yielding results close to 800 Mb (see Supplementary Table S2 and Supplementary Fig. S1), indicating that the electric eel's genome size is indeed approximately 800 Mb. This also corresponds well with our actual genome assembly results. Thank you once again for your invaluable suggestion.

3. There are also various RNA-seq datasets available, but the authors did not try to analysis the RNA-seq data. This manuscript provides only genomic description, thus very limited power in addressing the questions they brought up in the Introduction.
Response: Thank you for your suggestion. As we mentioned above, the main goal of this work is to provide a higher-quality reference genome for the electric eel within the field, so that other scientists in the field can join us in our efforts to study the key scientific questions of electric eel. Therefore, we submitted this manuscript as a Data Note to this journal. For several key scientific questions, we have conducted systematic research using many data (including the RNA-seq data) in 2-3 other works that we are currently preparing. Once again, thank you for your valuable suggestion.

4. TGS-gapcloser is known to cause false-positives in closing the gaps. The authors need to make sure they have manually inspected all gaps that were filled with TGS-gapcloser.
Response: TGS-GapCloser is currently a widely used software for filling gaps, as evidenced in studies on the Yangtze finless porpoise and East Asian finless porpoise (10.1093/gigascience/giae067), Hawaiian crickets (10.1038/s41467-024-49344-4), and snakes (10.1126/science.adj7026). As you mentioned, it may occasionally produce false positives when closing gaps. Therefore, we carefully re-examined these filled gaps using ONT ultra-long sequencing data. The results showed that all gaps filled by TGS-GapCloser were supported by ONT sequences, indicating that the gaps we filled using the software were indeed correct. Thank you for your suggestion.

Minor comments:
5. L45, it's not fully accurate to describe the fish as the oldest vertebrate group.
Response: Thank you for pointing out this issue. We changed the description with “As one of the oldest vertebrate groups”.

6. L77, there are also fish T2T genomes published, including the zig-zag eel.
Response: In this revision, we have included the zig-zag eel. Thank you for pointing out this issue.

7. L128, hifiasm produces two haploid genomes. Did the author randemly pick one, or simply use the

primary contigs?
Response: Yes, we used the primary contigs. Thank you.

8. L131, I do not suggest to do pilon polishing for HiFi assembly.
Response: As you mentioned, generally, it is not necessary to perform the polishing step when using only HiFi reads for contig-level assembly. However, to obtain a high-contiguity genome assembly, we combined PacBio HiFi reads and ONT ultra-long reads for contig assembly. As we all know, ultra-long reads typically have a relatively higher single-base error rate compared to HiFi reads. Therefore, we conducted Pilon polishing after the contig-level assembly, a practice that has been adopted in several high-quality publications. Thank you for your suggestion.

9. L236, in general, many analyses involve custom Perl scripts. All those scripts need to be uploaded to a public repository.
Response: As you suggested, we have uploaded the key Perl scripts that we wrote to the GigaDB in this revision. For other scripts that embedded in public software, we have provided detailed descriptions of the key parameters in the Methods section. Thank you for your suggestion.

10. L294, "the lack of a high-quality reference genome…" this is not true. At the VGP assembly is of high quality.
Response: We have changed the description with “Therefore, a genome of the electric eel with higher contiguity and completeness is urgently needed.” Thank you.

11. L298, the k-mer distribution plot looks very strange, without an obvious homozygous peak. I suggest to use a larger k-mer.
Response: Sorry for our inaccurate description. In fact, the k-mer distribution curve appears quite normal, featuring a very prominent main peak (homozygous peak) at a kmer depth of 83. We have corrected the writing in this revision. Thank you for bringing this issue to our attention.

12. L311, continuity should be contiguity? I do not see the main Tables.
Response: Thanks for your suggestion. In this revision, we changed the word “continuity” with “contiguity”. Regarding the main Tables, we have re-examined them in the merged PDF downloaded from the GigaScience website in this revision, and confirmed that all the main Tables are displayed normally in the manuscript. Regarding the Supplementary file (including supplementary tables and figures, especially the Supplementary Table S3), you could download it directly at the last page of the merged PDF file.

13. L346, having assembled the telomeres doesn't mean the genome assembly is telomere-to-telomere. In fact, there are still a few gaps in some chromosomes. The authors could at best describe some of the chromosomes T2T.
Response: Yes, we agree with the issues you raised. Therefore, in this revision, we revised this sentence to “Finally, we identified 46 telomeres and 26 centromeres in the chromosome-level genome, indicating that we have successfully obtained a high-quality reference genome for E. electricus, with many chromosomes achieving the telomere-to-telomere assembly”. Thanks for your suggestion.

14. The Discussion part is mostly a summary of the results, without providing useful insights or interpretations.
Response: Yes, we fully agree with the issues you raised. Therefore, in this revision, we have systematically and carefully revised and re-organized the Discussion section of the paper. Thank you for your valuable suggestion.

Reviewer #2:
Minor:
1."An individual electric eel (E. electricus) used in this study was procured from the aquatic pet market." - this is relevant. Is there a way to define the origin of the individual sampled?
Response: Based on existing records, the primary habitat of the electric eel is in the Amazon Basin of South America. However, the sample mentioned in this paper was obtained from the aquarium pet market in China. Therefore, in this revision, we have clearly stated in the Introduction section that its main distribution area is the Amazon River Basin, and specified in the Sampling section of the Methods that the

sample was acquired from the aquarium pet market in China. Thank you for your valuable suggestion.

2. "Our analysis identified 425 gene families that have undergone significant expansion" - it would be interesting to detail some genes specifically with phylogenetic analysis to validate that these expansions are artifacts.
Response: Thanks for your suggestion. We constructed the phylogenetic tree based on single copy homologous genes, and then inferred the divergence time among species based on the fossil data from TimeTree database. Based on the fossil-calibrated phylogenetic tree, we identified 425 expanded gene families in the electric eel genome. Subsequently, we extracted several genes for validation and confirmed to have indeed undergone expansion. Thanks again for your valuable suggestion.
