# Supplementary material for: Telomere-to-telomere genome assembly of Electrophorus electricus provides insights into the evolution of electric eels
Source: Gigascience. 2025 Apr 1;14:giaf024. doi: 10.1093/gigascience/giaf024 (PMC11959694; doi:10.1093/gigascience/giaf024)
Supplement: giaf024_Response_to_Reviewer_Comments_Revision_1 [file giaf024_response_to_reviewer_comments_revision_1.pdf]

Author's Response To Reviewer Comments

Reviewer #1: The authors wrote in the abstract conclusion that "...offers insights into the potential genetic mechanisms underlying the exceptional ability of electric eels to discharge high-voltage electricity", but I do not see any insights revealed by this manuscript. As the authors admitted that "The main goal of this work is to provide a higher-quality reference genome for the electric eel" and "we submitted this manuscript as a Data Note to this journal", I suggest to remove the statement in the abstract.
Response: Thank you for your suggestion. We have removed this statement and replaced with “This study not only provided the first high-quality telomere-to-telomere reference genome of E. electricus, but also greatly enhanced our understanding of electric eels.”.

The authors also responsed that "The results showed that all gaps filled by TGS-GapCloser were supported by ONT sequences, indicating that the gaps we filled using the software were indeed correct.". Please upload those results in the supplementary figures.
Response: Thank you for your suggestion. We have added the results in the Supplementary file in this revision.

The figure 6 looks suspicious. I suggest to add more individuals to confirm this trend.
Response: Thank you for your suggestion. We downloaded the genome assembly and sequencing data from two additional individuals and analyzed the population history with the same method in this revision. All the results consistently show a sharply decreased trend over the past few hundred thousand years. Therefore, in this revision, we revised the statement with “Interestingly, we also inferred the effective population size of E. electricus and found a sharply decreased trend over the past few hundred thousand years (Fig. 6), which is consistent with the results acquired from two additional individuals (Supplementary Fig. S7).”.
