# Supplementary material for: Telomere-to-telomere genome assembly of Electrophorus electricus provides insights into the evolution of electric eels
Source: Gigascience. 2025 Apr 1;14:giaf024. doi: 10.1093/gigascience/giaf024 (PMC11959694; doi:10.1093/gigascience/giaf024)
Supplement: giaf024_Reviewer_1_Report_Original_Submission — Luohao Xu -- 9/7/2024 [file giaf024_reviewer_1_report_original_submission.pdf]

Reviewer Report

Title: Telomere-to-telomere genome assembly of Electrophorus electricus provides insights into the evolution of electric eels

Version: Original Submission **Date:** 9/7/2024

Reviewer name: Luohao Xu

Reviewer Comments to Author:

Qi et al. described a high-quality of E. electricus with many chromosomes reaching the T2T level. While this genome assembly can serve as an interesting resource for a fish T2T genome, very few novel insights have been revealed by the analysis.There are at least three additional E. electricus genome assemblies available in NCBI. I am surprised that the authors mentioned none of them, nor comparing them with their assembly. Note the VGP E. electricus also has a high-quality with two haploid genomes assembled. Curiously, the genome sizes vary substantially among the assemblies: the VGP assembly has a ~600 Mb genome size, but the assembly from this study is larger than 800 Mb. Why?There are also various RNA-seq datasets available, but the authors did not try to analysis the RNA-seq data. This manuscript provides only genomic description, thus very limited power in addressing the questions they brought up in the Introduction.TGS-gapcloser is known to cause false-positives in closing the gaps. The authors need to make sure they have manually inspected all gaps that were filled with TGS-gapcloser.Minor comments:L45, it's not fully accurate to describe the fish as the oldest vertebrate group.L77, there are also fish T2T genomes published, including the zig-zag eel.L128, hifiasm produces two haploid genomes. Did the author randemly pick one, or simply use the primary contigs?L131, I do not suggest to do pilon polishing for HiFi assembly.L236, in general, many analyses involve custom Perl scripts. All those scripts need to be uploaded to a public repository.L294, "the lack of a high-quality reference genome…" this is not true. At the VGP assembly is of high quality.L298, the k-mer distribution plot looks very strange, without an obvious homozygous peak. I suggest to use a larger k-mer.L311, continuity should be contiguity?I do not see the main Tables.L346, having assembled the telomeres doesn't mean the genome assembly is telomere-to-telomere. In fact, there are still a few gaps in some chromosomes. The authors could at best describe some of the chromosomes T2T.The Discussion part is mostly a summary of the results, without providing useful insights or interpretations.

Level of Interest

Please indicate how interesting you found the manuscript: Choose an item.

Quality of Written English

Please indicate the quality of language in the manuscript: Choose an item.

Declaration of Competing Interests

Please complete a declaration of competing interests, considering the following questions:

- Have you in the past five years received reimbursements, fees, funding, or salary from an organisation that may in any way gain or lose financially from the publication of this manuscript, either now or in the future?
- Do you hold any stocks or shares in an organisation that may in any way gain or lose financially from the publication of this manuscript, either now or in the future?
- Do you hold or are you currently applying for any patents relating to the content of the manuscript?
- Have you received reimbursements, fees, funding, or salary from an organization that holds or has applied for patents relating to the content of the manuscript?
- Do you have any other financial competing interests?
- Do you have any non-financial competing interests in relation to this paper?

If you can answer no to all of the above, write 'I declare that I have no competing interests' below. If your reply is yes to any, please give details below.

'I declare that I have no competing interests

I agree to the open peer review policy of the journal. I understand that my name will be included on my report to the authors and, if the manuscript is accepted for publication, my named report including any attachments I upload will be posted on the website along with the authors' responses. I agree for my report to be made available under an Open Access Creative Commons CC-BY license (http://creativecommons.org/licenses/by/4.0/). I understand that any comments which I do not wish to be included in my named report can be included as confidential comments to the editors, which will not be published.

Choose an item.

To further support our reviewers, we have joined with Publons, where you can gain additional credit to further highlight your hard work (see: https://publons.com/journal/530/gigascience). On publication of this paper, your review will be automatically added to Publons, you can then choose whether or not to claim your Publons credit. I understand this statement.

Yes Choose an item.
